# Peptide Design for Targeting BTB Domain Homodimerization of BACH2: Complementary *In Silico* and *In Vitro* Approaches

**DOI:** 10.1021/acsomega.6c01122

**Published:** 2026-04-17

**Authors:** Efe Acar, Hüveyda Başağa, Emel Timuçin, Ahmet Can Timuçin

**Affiliations:** † Department of Molecular Biology and Genetics, Graduate School of Natural and Applied Sciences, 162328 Acibadem Mehmet Ali Aydinlar University, İstanbul 34752, Türkiye; ‡ Department of Molecular Biology, Genetics and Bioengineering, Faculty of Engineering and Natural Sciences, 52991 Sabancı University, İstanbul 34956, Türkiye; § Department of Molecular Biology and Genetics, Faculty of Science, Gebze Technical University, Kocaeli 41400, Türkiye; ∥ Department of Molecular Biology and Genetics, Faculty of Engineering and Natural Sciences, 162328 Acibadem Mehmet Ali Aydinlar University, İstanbul 34752, Türkiye

## Abstract

Potential activation of NRF2 via targeting its nuclear suppressor BACH2 represents an unexplored field, forming a gap yet to be addressed. Besides, considering the pathological role of BACH2 in tumor immunosuppression, an attempt to target this repressor was deemed necessary. Despite this background, a peptide-based modulator that could interfere with the BACH2 BTB domain has not been reported. Accordingly, using *in silico* and *in vitro* approaches, a BACH2 inhibitory peptide was identified via determining its affinity for BACH2 and demonstrating its negative impact on homodimerization. Specifically, a preliminary study was conducted to generate a peptide library that could target BACH2 BTB homodimerization. Initially, members of this peptide library were tested in terms of their capacity to bind the dimerization surface of the BACH2 BTB domain using docking programs. Second, selected BACH2 BTB domain-peptide complexes were analyzed for persistence of binding through 500 ns molecular dynamics simulations, and trajectories were also subjected to estimation of relative binding free energy levels. Following the computational approach, selected peptides, peptide 13 and control peptide 1, were ranked according to their dissociation constants to full-length BACH2 *in vitro* using supernatant depletion assay, with peptide 13 showing a trend toward higher affinity. Additionally, the inhibitory effect of peptides on homodimerization of the BACH2 BTB domain was investigated through coimmunoprecipitation studies, revealing peptide 13 as a potential disruptor of homodimerization. Based on these observations, peptide 13 has been identified as a putative BACH2 negative regulator. Furthermore, limitations and future directions are also discussed.

## Graphical abstract

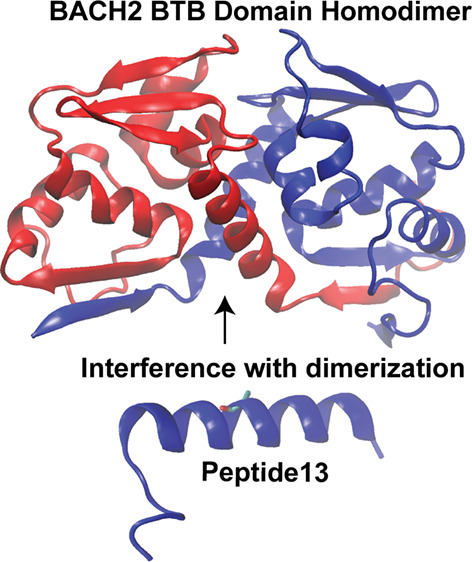

## 1 Introduction

The NRF2 (nuclear factor erythroid 2-related factor 2) transcription factor, encoded by the *NFE2L2* gene, belongs to the cap’n’collar transcription factor family.^1^ At its C-terminal region, NRF2 contains a basic leucine zipper (bZip) domain, which enables it to form heterodimers with other bZip family members, including the small MAF proteins MAFK, MAFG, and MAFF.^1–3^ These heterodimeric complexes regulate approximately 250 human genes containing antioxidant response elements and are central to the control of homeostatic processes related to inflammation, redox metabolism, and proteostasis.^1,4–7^ In light of these functions, NRF2 has been identified as a promising pharmacological target in clinical pathologies involving oxidative stress and inflammation, including neurodegenerative, vascular, metabolic, and cancer-related diseases.^1,8,9^ From a canonical perspective, NRF2 activation is predicted to support homeostasis in many chronic diseases and to confer beneficial therapeutic effects.^1^ The notion that activity of NRF2 in cytoplasm is regulated via a E3 ligase adaptor KEAP1 is well established and this mechanism has been used to activate NRF2 via several different molecule classes including KEAP1 inhibitors such as electrophiles and protein–protein interaction modulators.^1^ Nonetheless, it is also a solid fact that NRF2 has nuclear suppressors including BACH2 (transcription regulator protein BACH2 (BTB and CNC homologue 2)) of which still lack a modulator for inhibition. Thus, an attempt to design a peptide based BACH2 negative modulator was deemed as a rational approach, which prompted this study.

The BACH family transcription factors, BACH1 and BACH2, are classified within the bZip protein family and function as transcriptional repressors by forming heterodimeric interactions with small MAF proteins.^10,11^ Within the nucleus, BACH1 and BACH2 interact with MAF proteins and act as nuclear suppressors of NRF2, the established transcription factor protecting the cellular system against oxidative stress.^12–17^ While BACH1 is ubiquitously expressed across all cell types,^10^ with particularly high expression in hematopoietic cells,^18^ BACH2 expression is restricted to B cells, T cells, alveolar macrophages, and neural cells.^19^ This restricted expression pattern distinguishes BACH2 as a more selective therapeutic target compared with BACH1, making it a valid candidate for cell-type-specific targeting.

Both BACH1 and BACH2 contain an N-terminal BTB domain, which mediates homo- and heterodimer formation with itself or other factors.^11,20–22^ The BTB domain of BACH1 has been previously shown to be required for cytoplasmic-to-nuclear translocation.^21^ In that study, Kanezaki et al. demonstrated a truncated BACH1 isoform containing only the BTB domain that can interact with the full-length BACH1 BTB domain, thereby mediating BACH1 cytoplasmic-nuclear translocation.^21^ Parallel to these observations, a similar homodimerization mechanism has been suggested for BACH2 cytoplasmic-nuclear translocation.^22^ Moreover, a Leu24Pro mutation in the BACH2 BTB domain was also revealed to prevent interaction with full-length wild-type BACH2, most likely by disrupting the hydrophobic dimerization surface and leading to misfolding.^22^ Furthermore, analogous to BACH1, a truncated BACH2 isoform containing only the BTB domain has also been previously reported.^23^ Consistently, PDB (Protein Data Bank) entry for the BACH2 BTB domain confirms its homodimeric structural organization, as well.^24^ All of the background studies strongly underscore that the BTB domain homodimerization is necessary for BACH2 activity; thus, although indirectly, targeting this interaction might in turn represent a path to support NRF2 activation.

Apart from the recognized role as a transcriptional repressor of NRF2, BACH2 also takes part in immunological tolerance, immune memory, and immunosuppression.^22,25^ In light of these functions, specific mutations in BACH2 have been associated with autoimmune diseases such as asthma,^26^ multiple sclerosis,^27^ Crohn’s disease,^28^ and Hashimoto’s thyroiditis.^29^ These associations have been further supported by evidence from BACH2-deficient mice, which exhibit elevated eosinophil, macrophage, and effector CD4^+^ T cell populations in the lungs and secondary lymphoid organs.^30,31^ At the cellular level, the presence of BACH2 has been shown to be indispensable for the differentiation of both thymic and peripheral regulatory T cell populations of which its deletion results in severe inflammatory manifestations, highlighting its essential role in immune homeostasis.^25,30^ Of particular importance, regulatory T cells, whose differentiation depends on BACH2, was presented to restrict the activity of effector CD4^+^ and CD8^+^ T cell populations, thereby contributing to immunosuppression.^32^ In this context, BACH2 deficiency has been reported to suppress tumor growth, a phenomenon attributed to the increased infiltration of effector CD4^+^ and CD8^+^ T cells into tumors.^33^ Furthermore, it was demonstrated that such antitumor effects arising from BACH2 deficiency were also accompanied by a concomitant reduction in intratumoral regulatory T cell populations.^33^ At the molecular level, BACH2 is also known to be suppressor of IL2 receptor signaling, which is known to be required for Foxp3 expression, maintenance and suppressor functions at different stages of regulatory T cell populations.^34,35^ In line, BACH2 was shown to be required for stem-like CD8^+^ T cells during chronic viral infection.^36^ More recently, BACH2 activity was also linked to HIV-1 persistence in memory CD4^+^ cell,^37^ as well as, to beta cell failure in type 2 diabetes models.^38^ Together, these previous observations suggest that targeting BACH2 could represent an alternative immunomodulatory therapeutic strategy, potentially enhancing tumor recognition by the immune system.

With respect to BACH inhibition, all evidence to date currently lacks targeting toward BTB domain. In a previously reported attempt to inhibit BACH1, HPP-4382, a compound described by Attucks et al.,^39^ was presented to bind to the metalloporphyrin-binding bZip domain of BACH1, thereby inhibiting its activity and facilitating NRF2 access to its target genes. In this context, a recent study provided novel insights into drug development efforts targeting the immunosuppressive role of BACH2.^40^ In that work, a stable cell-based bioluminescence assay was established, in which tetracycline-induced BACH2 expression was shown to suppress the activation of a PMA (phorbol 12-myristate 13-acetate)/ionomycin-dependent interferon gamma +18k enhancer luciferase reporter.^40^ Using this system, the histone deacetylase 3 (HDAC-3) inhibitor RGFP966 was identified as a BACH2 inhibitor.^40^ This compound was, in fact, used as a positive control because BACH2-mediated repression of BLIMP-1 (B lymphocyte-induced maturation protein-1) in B cells has been demonstrated to be HDAC-3 dependent.^40^ Despite these attempts, it was clear that rational design of a peptide that can interfere with homodimerization of BACH2 BTB domain could bring novel insights into both indirect NRF2 activation and possible prevention of BACH2 mediated immunosuppression.

In the hope of expanding this background, here we report *in silico* and *in vitro* identification of a peptide capable of targeting BACH2 BTB domain homodimerization. In order to achieve this outcome, first a preliminary computational study was conducted. Through this initial study, preliminary peptide definition, its sequence optimization and generation of a peptide library based on this sequence optimization were completed. After the initial step, members of the peptide library were subjected to computational structure prediction and through global docking, some these peptides were selected for extensive 500 ns molecular dynamics (MD) simulations. By MM-PBSA (Molecular Mechanics-Poisson–Boltzmann Surface Area continuum solvation) based relative binding free energy (RBFE) analyses, peptide 13 was selected and was subjected to in depth MD analyses together with the control peptide. Since extensive MD analyses supported MM-PBSA results, peptide 13 and control peptide was fed into *in vitro* analyses in which a supernatant depletion assay (SDA) was performed for each peptide to reveal each of their dissociation constant (*K*_d_). At the final step, to confirm the functionality, the coimmmunoprecipitation method was employed to decipher the impact of peptides in BACH2 homodimerization. Overall, here for the first time in literature, we are presenting complementary computational and *in vitro* evidence on a peptide capable of disrupting the BACH2 BTB domain homodimerization.

## 2 Materials and Methods

### 2.1 Protein Structure and Peptide Prediction

BACH2 BTB domain homodimer (PDB ID: 3OHU) was used as the reference structure in the study^24^ The most important feature of this structure was the absence of intermonomeric disulfide bonds present in PDB ID: 3OHV and, thus its accessibility for peptide design. Initial preliminary peptide prediction was completed using Peptiderive program.^41–43^ For prediction of the peptide, the same peptide sequence was predicted regardless of whether chain A or B was chosen as the receptor or partner from BACH2 BTB domain homodimer (PDB ID: 3OHU). Based on this observation, chain A was used as the receptor in all docking attempts. The predicted peptide sequence was retrieved from chain B.

### 2.2 FoldX-Based Mutational Optimization

The semiempirically validated FoldX 5.0 program^44,45^ calculates the free energy of unfolding as a ΔΔ*G* value by comparing between wild type (Δ*G*_wt_) and mutant Δ*G*_mt_) structures. The ΔΔ*G* value indicates the stability that the mutant amino acid can confer to the structure, where values above +0.5 kcal/mol indicate structural destabilization and values below −0.5 kcal/mol indicate increased structural stability. When this value remains between −0.5 and +0.5 kcal/mol, it is considered statistically insignificant. For execution of the FoldX program, the homodimeric BACH2 BTB domain structure (PDB ID: 3OHU) was first repaired using the *RepairPDB* command for analysis. Following this correction process, the ΔΔ*G* value estimation for interaction energy was computed using the *pssm* command.

### 2.3 ClusPro-Based Global Docking of Peptides

The ClusPro program^46^ was used for global docking search of the peptides to target BACH2 BTB domain. This program completes the docking process in 3 fundamental steps. First, billions of conformations are sampled through FFT (Fast Fourier Transform)-based rigid body docking. Since ClusPro is based on FFT, it can determine the structural region where binding is most likely to occur without a priori knowledge, and this feature was used to predict the regions where peptides would prefer binding. Second, the largest cluster is determined from among the 1000 lowest energy structures through clustering based on the root-mean-square deviation. Finally, the structures are refined through energy minimization. During implementation step of this program, BACH2 BTB domain monomer chain (PDB ID: 3OHU) and 16 peptides whose structures were predicted with PEP-FOLD 3.5,^47–49^ were used. To increase the reliability of the ClusPro algorithm, each peptide ligand and target protein monomer were individually simulated for 20 ns through MD simulations in a solvated and ionized environment before docking runs. The structure showing the closest RMSD (root mean square displacement) to the average structure was taken from the trajectories of the simulations exhibiting RMSD-based conformational stability. This selected structure was subjected to docking analysis within the ClusPro program. For selecting peptides after ClusPro, PLIP program was used to assess weak interaction network between peptides and the target.^50^

### 2.4 HADDOCK-Based Local Docking of Peptides

The complexes determined through global docking were reconstructed using the HADDOCK 2.2 ^51,52^ local docking program, thereby ensuring that MD simulations were run with more accurate conformations. For execution of the HADDOCK program, ClusPro based local docking revealed amino acids responsible for the interactions were defined as active, while other amino acids in the BACH2 BTB domain, BACH1 BTB domain, and BCL6 BTB domain structures located outside the 6.5 Å vicinity of the actives were defined as passive. During the HADDOCK run, 1000 structures were generated for rigid body docking, 200 structures for semiflexible refinement, and 200 structures for explicit solvent refinement. All results were clustered with a 7.5 Å limit based on the RMSD. Following clustering, poses were selected according to HADDOCK score and cluster size. The generated complexes were fed into the MD simulation protocol.

### 2.5 MD Simulations and Analyses

Prior to simulations, following the retrieval of structures from local docking, PROPKA program within the PDB2PQR was used to determine the protonation states of histidines in target proteins and peptides.^53^ For simulations, all complexes were first solvated in water and neutralized with 150 mM NaCl. The generated systems were used with the NAMD program within CHARMM36 parameters.^54–57^ Water molecules within the systems were modeled according to the TIP3P model.^58^ In all simulations, an isothermal–isobaric (*NpT*) ensemble was used with periodic boundary conditions. Long-range Coulomb interactions were calculated by using the particle mesh Ewald algorithm. A distance cutoff of 17 Å was used for calculating nonbonded electrostatic interactions and van der Waals interactions. Pressure was maintained at 1 atm and temperature at 310 K using the Langevin piston Nosé–Hoover method.^59^ Hydrogen bond lengths were constrained using the SHAKE algorithm^60^ and system coordinates were recorded every 2 ps. All systems were initially energy minimized using the conjugate gradient method in 20,000 steps. Subsequently, the temperature of each system was gradually raised from 10 to 300 K in 30 ps. In the final step before production, each system was subjected to a 20 ns equilibration run with production runs designed to be 500 ns. Each production run was repeated 4 times.

All protein–protein complex structures and trajectories were analyzed using the Visual Molecular Dynamics (VMD) program.^61^ The convergence of simulations to conformational stability was calculated according to RMSD analysis of backbone atoms (C, N, Cα, and O) during each production simulation. In preliminary peptide 100 ns MD runs, pairwise RMSD analysis using Cα was also included. Hydrogen bond analyses were completed using the H bonds plugin available within VMD. In this analyses, the unique option was selected to provide analysis based on occupancy. Within this plugin, hydrogen bonds were identified using a donor–acceptor distance cutoff of 3 Å and a donor–hydrogen–acceptor angle cutoff of 20°. The newcontact.tcl script was used for hydrophobic interaction analysis, which a search of carbon–carbon distance less than 5 Å was searched in between selected residues. Heavy atom RMSF (Root mean square fluctuation) calculations of selected amino acids and SASA (Solvent accessible surface area) analyses of target chain A monomer of BACH2 BTB domain were also completed using Tcl scripts using the Tcl/Tk console of VMD program. For PCA analysis, in the first step, each frame was superimposed on the initial structure of the simulations, eliminating translational and rotational movements and isolating the internal movements of the system. PCA analysis was applied to the diagonalized Cartesian covariance matrix to obtain a set of eigenvectors and corresponding eigenvalues. PCA was employed to backbone atom Cα in all systems. Eigenvalues were calculated as mean-square fluctuations in the principal component direction, and the highest eigenvalue was determined as the collective motion performed with the highest dominance. Results were shown through graphs of eigenvalues versus eigenvector indices, graphs of principal components relative to each other, and the interpolation of motions on the structure. Additionally, motions performed during simulations were evaluated through cross-correlation analysis, and possible correlations between dominant collective motions via PCA analysis were revealed. All PCA and cross-correlation analyses were completed using the ProDy python software package.^62–64^

### 2.6 Estimation of MM-PBSA-Based Relative Binding Free Energy

For MM-PBSA based relative binding energy calculations, the last 20 ns encompassing frames of the common conformationally stable regions (the last 250 ns of the total 500 ns) within the MD trajectories were used. During calculations, the internal dielectric constant was taken as ϵ = 1, and the external dielectric constant was taken as ϵ = 80. Trajectories taken from 20 ns portion was divided into 50 frames of 400 ps frames. MM-PBSA calculations were completed using the MM-PBSA.py algorithm available in AmberTools 23.^65^ CHARMM parameters were used within AMBER using the chamber command of the ParmED program found under AmberTools.^66^ When necessary, statistical significance between experimental groups was analyzed using *t* test statistics.

### 2.7 Peptides for Supernatant Depletion Assay and Coimmunoprecipitation Experiments

Computationally selected peptides were synthesized and purchased from BIOMATIK. In summary, control peptide 1 and peptide 13 were synthesized with or without the C-terminal biotin tag, without TFA removal, at 95% purity and 100 mg quantity. Lyophilized peptides were stored at −20 °C up until the execution of the experiments.

### 2.8 HEK293T Cell Culture

HEK293T was used as the cell line to produce tagged protein overexpression containing target protein for supernatant depletion analysis and coimmunoprecipitation assays as it has been previously used for BACH2 overexpression.^22^ HEK293T cells were cultured in high glucose DMEM + 10% FBS medium at 37 °C and 5% CO_2_ conditions using the necessary cell culture equipment. T-25 and T-75 flasks were used to achieve optimum cell growth. Subculturing was completed at 1:5, 1:20, or 1:100 dilution depending on the planned experiment. For subculturing, cells were removed with 0.25% Trypsin-EDTA solution. Medium was changed every 3 days. For long-term storage, cells were kept in liquid nitrogen within cryotubes containing 10% DMSO-complete medium mixture. All experiments were implemented within 7 passage intervals. Cell growth was confirmed via counting with hemocytometer when deemed necessary.

### 2.9 Plasmid Amplification and Isolation

Control backbone plasmid capable of expressing only myc-DDK tag (pCMV6-Entry, mammalian vector with C-terminal Myc-DDK Tag, PS100001), control backbone plasmid capable of expressing only tGFP tag (pCMV6-AC-GFP Mammalian Expression Vector, PS100010), myc-DDK tagged BACH1 overexpression plasmid intended for use in negative control experiments (BACH1 (NM_206866) Human Tagged ORF Clone-RC221628), and myc-DDK or tGFP tagged BACH2 overexpression plasmid (BACH2 (NM_021813) Human Tagged ORF Clone RC214061, RG214061) were purchased from ORIGENE. Plasmids were purchased ready for transfection as 10 μg, dissolved in 100 μL sterile water, and stored at −20 °C at a concentration of 0.1 μg/μL. For plasmid amplification, 1 pg–100 ng of plasmid DNA was used within chemically competent DH5α *E. coli* cells. The transformation protocol began with a 30 min incubation following the mixing of DNA and cells. Following incubation, heat shock was performed at 42 °C for 30–35 s. After heat shock, cells were incubated on ice was for 2 min which was followed by recovery phase through adding SOC medium. The culture with added SOC medium was incubated at 37 °C for 60 min at 200 rpm. In the final phase, plating was performed on solid LB containing kanamycin (25 μg/mL) or ampicillin (100 μg/mL) with 5–200 μL samples taken from the cells in SOC medium, and this plate was incubated at 37 °C for 16–18 h. Subsequently, samples that were taken from colonies cultured on the Petri dish were further grown in volumes appropriate for Mini-Prep plasmid isolation protocol with liquid LB containing kanamycin (25 μg/mL) or ampicillin (100 μg/mL). Planned volume of the bacterial cell culture was subjected to plasmid isolation, while the remaining part was stored at −80 °C with glycerol stock for future use. Bacterial cell culture taken from liquid LB was subjected to plasmid isolation mini-prep protocol to make plasmids suitable for transfection into HEK293T cells. QIAprep Spin Miniprep Kit was used for plasmid isolation. In summary, 1–5 mL of overnight grown culture for 16–18 h was centrifuged at >8000 rpm (6800*g*) for 3 min at room temperature. The cell pellet was taken into a microcentrifuge tube in 250 μL of P1 buffer. Subsequently, 250 μL of P2 buffer was added to the cell pellet dissolved in P1 buffer, mixed by inverting 4–6 times, and incubated for 5 min to achieve cell lysis. Following this step, 350 μL of N3 buffer was added. Then centrifugation was performed at 13000 rpm (17,900*g*) for 10 min, and the supernatant was transferred to the QIAprep spin column with 30–60 s centrifugation. The spin column was washed with 0.5 mL PB buffer by 30–60 s centrifugation. Following this step, washing was performed with 0.75 mL PE buffer again by 30–60 s centrifugation. In the final step before elution, excess wash buffer was cleaned by 1 min centrifugation. The QIAprep column was transferred to a clean 1.5 mL microcentrifuge tube, incubated with 50 μL EB buffer (10 mM Tris–Cl, pH 8.5) for 1 min, and then elution was performed with 1 min centrifugation. Since a maximum of 20 μg of plasmid DNA isolation could be achieved with this protocol, the protocol was repeated when necessary.

### 2.10 Myc-DDK- and tGFP-Tagged BACH2 Overexpression and Protein Extraction

For transient transfection experiments in SDA, 60 mm cell culture dishes were used. From a fully confluent T25 plate, cell dilution was prepared at a 1:10 ratio to seed 60 mm plates and overnight incubation was provided to ensure attachment to the plate bottom. Thus, 50–70% confluency was achieved the next day. Three μg DNA was subjected to dilution with 750 μL serum-free medium, followed by 9 μL Turbofectin transfection agent that was added to this mixture and incubated at room temperature for 15 min. Subsequently, this mixture was added drop by drop to 60 mm plates that contain HEK293T cells and left for 48 h incubation at 37 °C—5% CO_2_. At the end of 48 h, the cell media were removed, cells washed once with PBS were transferred from the plate surface to 15 mL falcons with a scraper. After 125 × *g* 5 min centrifugation, cells were directed to the protein extraction phase. For coimmunoprecipitation experiments, 100 mm cell culture dishes were used. From a fully confluent T25 plate, cell dilution was prepared at a 1:5 ratio to seed 100 mm plates, and overnight incubation was provided to ensure attachment to the plate bottom. Thus, 50–70% confluency was achieved the next day. Fifteen μg DNA (7.5 μg DNA from each plasmid due to cotransfection) was subjected to dilution with 1500 μL serum-free medium followed by 45 μL Turbofectin transfection agent that was added to this mixture and incubated at room temperature for 15 min. Subsequently, this mixture was added drop by drop to 100 mm plates and left for 48 h incubation at 37 °C—5% CO_2_. At the end of 48 h, the cell media were removed, cells washed once with PBS were transferred from the plate surface to 15 mL falcons with a scraper. After 125 × *g* 5 min centrifugation, cells were directed to the protein extraction phase. M-PER Mammalian Protein Extraction Reagent (Thermofisher) was used for total soluble protein extraction, as described below. To the solution containing ready extraction buffer provided with the kit, protease and phosphatase inhibitor cocktails as well as 1 mM PMSF were added before any application. For HEK293T cells which is an adherent cell line, the medium was first discarded and washing with PBS was performed. 250 μL extraction solution was added for each 60 mm plate and incubated for 5 min. 500 μL extraction solution was added for each 100 mm plate and incubated for 5 min. Each lysate was then transferred to a microcentrifuge tube, centrifuged at 14,000 × *g* for 15 min, and the supernatant was transferred to another tube to be made ready for analysis. Protein amount was determined using the Pierce Coomassie Plus (Bradford) Assay Kit.

### 2.11 Immunoblotting

Target amount of protein extract was mixed with gel loading buffer containing 500 mM DTT or 5% beta-mercaptoethanol reducing agents, separated by 10% SDS-polyacrylamide gel, and transferred to the PVDF membrane. The PVDF membrane was blocked with 5% nonfat milk powder prepared in PBS-Tween-20. Subsequently, 16 h incubation was performed with primary antimyc tag antibody (Cell signaling, Myc-Tag Rabbit mAb (71D10), 2278) or anti-tGFP antibody (Origene, Mouse monoclonal turboGFP antibody clone OTI2H8, TA150041). After this incubation, following washing of the PVDF membrane with PBS-Tween20, HRP-labeled secondary antibody incubation was performed. Following the final wash with PBS-Tween20, luminescence in bands was analyzed using the SuperSignal West Pico PLUS Chemiluminescent Substrate. HRP-labeled antirabbit (Cell Signaling, Antirabbit IgG, HRP-linked Antibody, 7074) or HRP-labeled antimouse (Antimouse IgG, HRP-linked Antibody, 7076) antibodies obtained from Cell signaling company were used as secondary antibodies. In immunoblotting, the beta-actin amount was controlled with antibeta-actin antibody as the gel loading control (Cell signaling 4970). Imaging was completed with a BIORAD Chemi-Doc system. Immunoblotting method was used to test whether myc-DDK tagged BACH2 protein overexpression (calculated ∼95 kDa, appearing ∼130 kDa in gel), myc-DDK tagged BACH1 protein overexpression (calculated 82 kDa, appearing ∼100 kDa in gel), and tGFP tagged BACH2 overexpression (calculated ∼100 kDa, appearing ∼150 kDa in gel) were successful, for SDA and coimmunoprecipitation analysis. In coimmunoprecipitation experiments, after cotransfection, a total of 20 μg protein was used for immunoblotting.

### 2.12 Supernatant Depletion Assay

At this stage, C-terminally biotinylated peptides were first incubated at varying concentrations with streptavidin-coated Dynabeads (Dynabeads M-280 Streptavidin). It has been previously reported that Dynabeads-streptavidin has a binding capacity of 200 pmol of biotin-labeled peptide per mg of beads. Since there was no direct information regarding the affinities of the selected peptides (peptide 13 and the control peptide 1 toward BACH2 BTB domain), the experimentally determined *in vitro* *K*_d_ of the BACH2–heme interaction was used as a starting reference for peptide affinity analysis (BACH2–heme *K*_d_ = 166.2 nM).^67^ Accordingly, the amount of peptide bound to streptavidin-Dynabeads was adjusted within a total reaction volume of 50 μL to cover concentrations around this *K*_d_: approximately 1200 (60 pmol), 600 (30 pmol), 300 (15 pmol), 150 (7.5 pmol), 75 (3.75 pmol), and 0 nM (0 pmol). Based on the molecular weight and purity of the peptides, detailed calculations were performed, and the resulting graphs were plotted according to the exact concentrations used. The protocol briefly consisted of incubating the selected peptides with beads in PBS (pH 7.4) for 30 min. Following incubation, peptide-bound beads were separated magnetically and subsequently blocked with 0.1% BSA in PBS by three washing steps. For the receptor used in the SDA, supernatants from HEK293T cells overexpressing myc-DDK-tagged BACH2 or myc-DDK-tagged BACH1 were employed. In this context, it has been previously reported that total protein extracts are suitable for use in depletion assays and do not require prior purification.^68^ The concentration of the total protein extract containing myc-DDK-tagged BACH2 was kept constant during the assay (0.15 μg/μL). Theoretically, in order to reach the equilibrium *K*_d_, the receptor protein concentration should be maintained at a value lower than the true *K*_d_ (preferably at least 10-fold lower).^68^ However, since the actual concentration of the overexpressed protein in the supernatant could not be determined, the estimated *K*_d_ was treated as representing a nonequilibrium value and was compared to the *K*_d_ determined for the control peptide. During SDA, precipitation reactions containing only Dynabeads-streptavidin were included as negative controls. In this way, nonspecific binding was incorporated as a parameter in the estimation of the *K*_d_. In summary, approximately 7.5 μg of cell lysate was incubated with varying peptide concentrations. Specifically, C-terminally biotin-labeled peptides bound to streptavidin-Dynabeads were incubated for 30 min in separate microcentrifuge tubes containing receptor-containing supernatant, and complexes were separated magnetically. Aliquots of the supernatant (2.27 μg) were subsequently analyzed by immunoblotting using antimyc antibody to detect residual myc-DDK-tagged BACH2 or BACH1. The myc signal from the tube containing 0 nM peptide was normalized to 100%, and the percentage of depleted myc-DDK-tagged BACH2 molecules in the supernatant was calculated for increasing peptide concentrations. The resulting plots of peptide concentration versus percentage depletion were analyzed using GraphPad Prism software to estimate *K*_d_ under nonequilibrium conditions. All of the assays were repeated twice.

### 2.13 Coimmunoprecipitation

The cell culture, plasmid isolation, cotransfection, and immunoblotting control of overexpression necessary for the coimmunoprecipitation studies were described in the previous sections. For the coimmunoprecipitation study, 7 μg of protein extract containing both Myc-DDK tagged and tGFP tagged full-length BACH2 was used. In the first stage, the protein to be used was subjected to a preclearing step with 100 μL PBS and 150 μg Dynabeads-protein G conjugate to prevent nonspecific binding. To generate antibody-bound Dynabeads-protein G conjugate, 2 μg anti-tGFP antibody (Origene, Mouse monoclonal turboGFP antibody clone OTI2H8, TA150041) or 5 μg Mouse (G3A1) IgG1 kappa isotype control antibody (Cell Signaling, Mouse (e7Q5L) mAb IgG2b Isotype control, 53484) was incubated with 150 μg Dynabeads-protein G conjugate in 100 μL PBS for 10 min. The antibody-bound Dynabeads-protein G conjugate was washed twice with 200 μL of PBS-Tween20 prior to protein incubation. The precleared protein extract was incubated for 10 min with the washed antibody-bound Dynabeads-protein G conjugate or with Dynabeads-protein G conjugate alone, and the resulting complex was washed three times with 200 μL of PBS-Tween20. Proteins within the complex were analyzed by immunoblotting for myc signals. In systems using peptides, the protein extract was incubated with the relevant peptide concentration in 100 μL of PBS for 30 min prior to preclearing.

## 3 Results

To identify inhibitory peptides targeting the BACH2 BTB domain homodimer interface, we employed an integrated computational and experimental strategy (Figure 1). Computational screening and MD simulations prioritized Peptide 13 as the lead candidate, which was subsequently validated experimentally, demonstrating a nanomolar binding affinity and effective disruption of BACH2 homodimerization. The details of these results are given as below.

**Figure 1:**
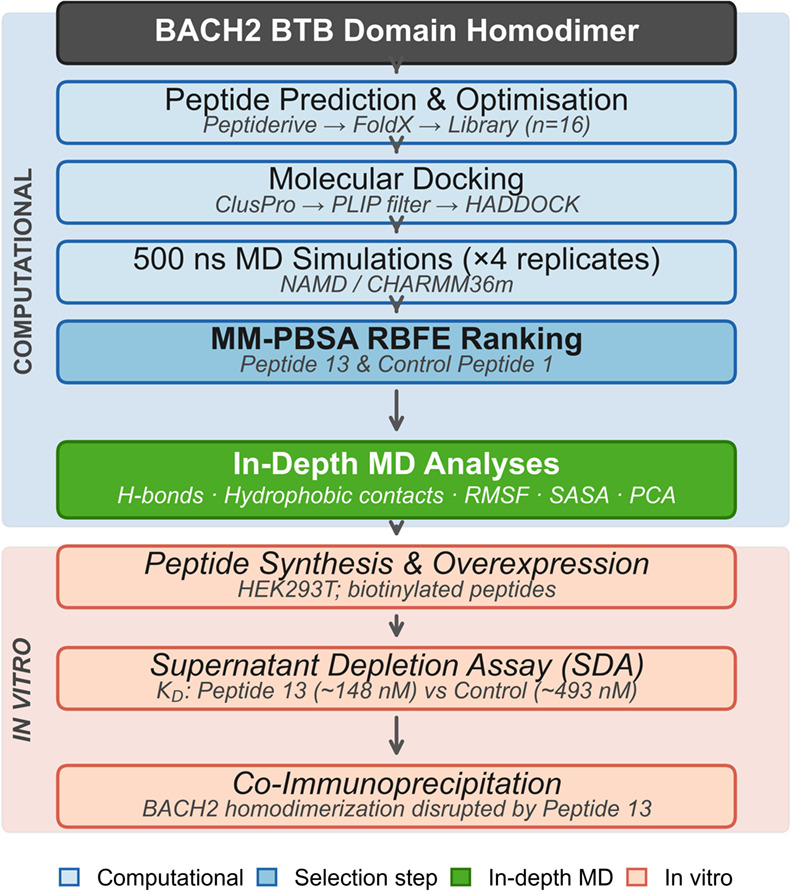
Integrated computational and experimental workflow for the identification of peptide-based BACH2 BTB domain homodimer inhibitors. The framework encompasses peptide prediction and optimization, molecular docking, 500 ns MD simulations, MM-PBSA-based candidate ranking, and in-depth MD analyses, culminating in experimental validation via supernatant depletion assay and coimmunoprecipitation.

### 3.1 Structure-Based Peptide Prediction and Optimization

Currently, PDB contains only two dimeric structures of the BACH2 BTB domain.^24^ One of these structures (PDB ID: 3OHV) has a disulfide bridge that holds the two chains (Chain A and B) together, while the other structure (PDB ID: 3OHU) does not include this interchain disulfide bond. To design a suitable peptide that could potentially inhibit dimerization of the BACH2 BTB domain, the structure lacking the disulfide bond (PDB ID: 3OHU, Chains A and B) was utilized (Figure 2A). The selected homodimeric structure of the BACH2 BTB domain was subjected to the Rosetta Peptiderive program^41–43^ to predict a 15 amino acid linear peptide sequence (derived from chain B monomer) which was chosen due to its highest energetic contribution to maintain the homodimeric state (Figure 2B and Table 1). After identifying its location, the interactions mediated by this predicted peptide sequence with the other monomer on the native homodimeric structure of the BACH2 BTB domain were investigated using the Ligplot + program,^69,70^ which can display possible hydrogen bonds and hydrophobic contacts (Supplementary Figure 1). This analysis revealed that the predicted peptide sequence in chain B interacts with the Ala97–Arg103 region of receptor monomer chain A primarily through hydrogen bonds using its amino acids in the Met11–Ser16 range (Supplementary Figure 1). Although several amino acids in the peptide sequence were shown to interact with the receptor monomer also through hydrophobic interactions (Supplementary Figure 1), it is well-known that hydrogen bonds govern specific binding. Therefore, the Ala97-Arg103 region of the Chain A BACH2 BTB domain monomer provided a rational basis for selecting the region where the peptide sequence would be preferentially targeting. In the next stage, the preliminary peptide sequence (Table 1) was isolated from the whole monomer and subjected to structure prediction and local docking procedures to prepare the protein-peptide structures for MD simulations. To ensure that the designed peptide has potential for *in vitro* and in vivo applications, a cell-penetrating peptide sequence (HIV TAT 47–57) was added to the N-terminal of the predicted peptide sequence (Table 1). The sequence provided in Table 1 was first subjected to the PEPFOLD 3.5 ^47–49^ program to predict the isolated structure of the peptide (Figure 2C). As shown in Figure 2C, while the predicted peptide structure (PP) did not adopt a secondary structure, the cell-penetrating peptide sequence (CPP-HIV Tat47–57) added to the N-terminal assumed an α-helical conformation as expected.^71^ After the prediction of the peptide structure, preliminary peptide was docked to the BACH2 BTB domain monomer using HADDOCK 2.2,^51,52^ a local docking method that allows flexibility in the protein backbone and side chains during docking. For the local docking procedure, amino acids 12–26 of the predicted region of the peptide were identified as active. On the target BACH2 BTB domain monomer, the region responsible for hydrogen bonding (amino acids 97–103) (Supplementary Figure 1) was selected as active for the local docking procedure. After the HADDOCK based local docking procedure, the protein-peptide structure with the highest HADDOCK score and the most sampled cluster was selected as the structure to be used in subsequent MD simulations. The structure selected from the HADDOCK local docking algorithm is shown in Figure 2C.

**Figure 2:**
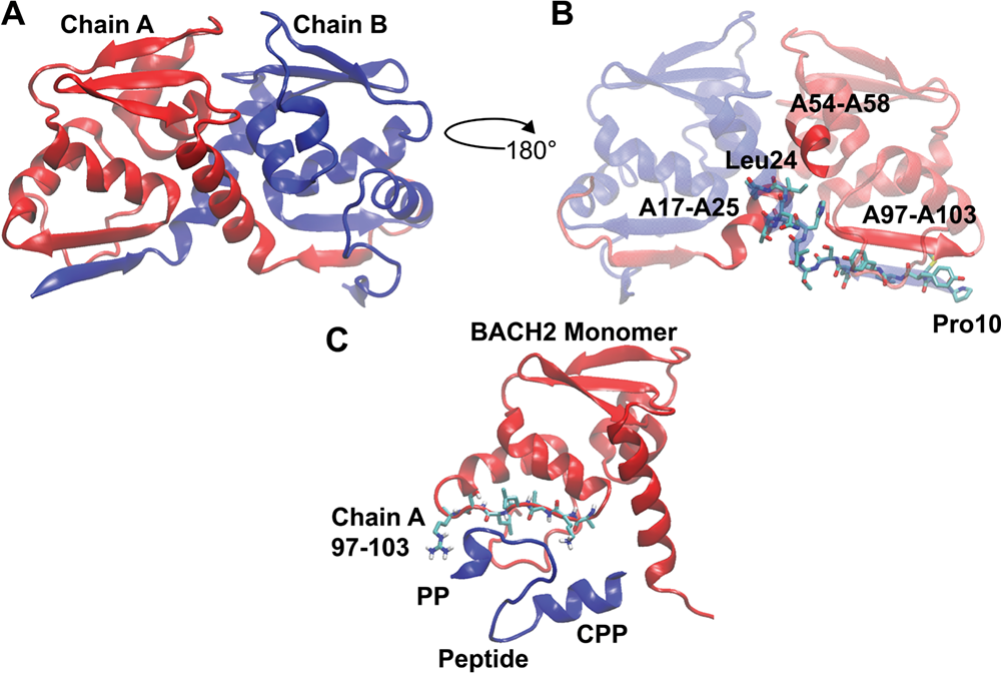
Predicted wild-type peptide targeting BACH2 BTB domain dimerization and potential molecular interactions with the opposing monomer. (A) The crystal structure of the BACH2 BTB domain homodimer (PDB ID: 3OHU, Chains A and B). (B) 15 amino acid long peptide sequence (Pro10-Leu24 shown in licorice representation), predicted on Chain B by the Rosetta Peptiderive program,^41–43^ is shown. Moreover, along with the predicted peptide, potential sequences (A17–A25, A54–A58, A97–A103) on the target BACH2 BTB domain monomer (Chain A) that could potentially interact with the peptide is shown. Exact information on interacting amino acids is shown in Supplementary Figure 1. (C) Cell-penetrating peptide (CPP) sequence (HIV TAT 7–57) amino acid sequence (1–11) was added to the N terminal of the predicted peptide (PP) sequence. This sequence was subjected to peptide structure prediction using the PEPFOLD 3.5 ^47–49^ program and the final peptide structure was locally docked to the chain A amino acids in between 97 and 103 region of BTB domain monomer of BACH2. The target region on chain A monomer was selected due to a high number of hydrogen bonding observed in the native structure (Supplementary Figure 1). The resulting pose was shown to depict the initial protein–peptide structure that has been fed into MD simulations. Red: BACH2 Monomer (Chain A), blue: peptide (CPP+PP).

**Table 1:** WT Peptide Sequence Designed from BACH2 BTB Domain Chain B Used in Docking, MD Simulations, and RBFE Estimation in the Preliminary Study

peptide name	sequence
preliminary control peptide 1	YGRKKRRQRRR^a^-PMYVYESTVHCTNIL^b^

a 11 amino acid long cell-penetrating peptide sequence (HIV Tat 47–57).

b Wild-type peptide sequence predicted from the BACH2 BTB domain homodimer (Chain B).

In the next step, BACH2 BTB domain monomer-preliminary peptide structure selected from the local docking procedure performed with HADDOCK, along with the positive control complex, BACH2 BTB domain homodimer available in the protein database, were subjected to 100 ns MD simulations. Using these MD simulations, RBFE was estimated for the BACH2 BTB domain monomer-preliminary peptide and positive control homodimeric BACH2 BTB domain complexes. To determine which parts of the trajectories from these initial MD simulations were suitable for the RBFE calculations, convergence of conformational stability was assessed using RMSD analysis (Supplementary Figure 2). First, the all-to-all RMSD analyses of the BACH2 BTB domain monomer-preliminary peptide complex were performed, revealing that conformational stability was maintained with RMSD fluctuations below ∼3 across all simulations, at least between 10 and 100 ns (Supplementary Figure 2A–C, corresponding to experimental replicates 1, 2, and 3). In parallel, the all-to-all RMSD analyses of the positive control BACH2 BTB domain homodimer structure showed a stable conformation with RMSD oscillations below ∼3 Å throughout the 100 ns simulation (Supplementary Figure 2D, showing the result of the first replica). Additionally, the RMSD versus simulation time graph indicated stable conformations with RMSD values below ∼3 Å for all simulations (Supplementary Figure 2E). Based on all these results, it was concluded that the 10–100 ns interval of the analyzed protein–peptide and protein–protein simulations could be used for estimation of the RBFE.

The last 10 ns segments (between 90th and 100th ns) from the selected last 90 ns part of all simulations were used to estimate the RBFE using the MM-PBSA method. When compared with the RBFE between the BACH2 monomers in the homodimeric BACH2 BTB domain complex, the energy of the designed peptide binding to the BACH2 BTB domain monomer was increased in a statistically significant manner (∼21 fold, *p* ≤ 0.05) (Δ*G* (Interaction), Table 2), verifying the preliminary peptide‘s potential for targeting homodimerization of BACH2 BTB domain. Following the estimation of RBFE, components of this energy (electrostatic vs nonpolar contributions) were analyzed to understand how the designed peptide utilizes them to achieve a high RBFE compared with the positive control (Table 2).

**Table 2:** Components of RBFE for the BACH2 BTB Domain Monomer–Preliminary Peptide Complex and Positive Control Homodimeric BACH2 BTB Domain Complex Retrieved in Preliminary Study^a^

	BACH2 BTB domain monomer-peptide complex		homodimeric BACH2 BTB domain complex	
	energy (kcal/mol)	±SEM	energy (kcal/mol)	±SEM
Δ*E* (VDWAALS)	–64.0	4.4	–227.4	2.2
Δ*E* (EEL)	–878.1	67.7	–327.6	2.1
Δ*E* (EPB)	+869.1	64.7	+421.3	4.6
Δ*E* (ENPOLAR)	–67.1	2.7	–175.0	1.0
Δ*E* (EDISPER)	+114.7	6.7	+307.5	1.4
Δ*G* (gas)	–942.1	69.1	–555.0	5.0
Δ*G* (solvation)	+916.7	65.7	+553.8	5.0
Δ*G* (interaction)	–25.4	5.2	–1.2	4.9

a Δ*E* (VDWAALS): van der Waals molecular mechanics energy, Δ*E* (EEL): electrostatic molecular mechanics energy, Δ*E* (EPB): polar contribution to the solvation energy, Δ*E* (ENPOLAR): nonpolar contribution of repulsive solute–solvent interactions to the solvation energy, Δ*E* (EDISPER): nonpolar contribution of attractive solute–solvent interactions to the solvation energy, Δ*G* (gas): total gas-phase molecular mechanical energy, Δ*G* (solvation): total solvation energy, Δ*G* (interaction): total RBFE, SEM: standard error of the mean (calculations were completed in triplicate for the protein–peptide complex and duplicate for the positive control).

Among these components: (1) by adding Δ*E* (VDWAALS) and Δ*E* (EEL), the total gas phase molecular mechanics energy, Δ*G* (Gas), was obtained; (2) by adding Δ*E* (EPB), Δ*E* (ENPOLAR), and Δ*E* (EDISPER), the total solvation energy, Δ*G* (Solvation), was obtained; 3) By adding Δ*G* (Gas) and Δ*G* (Solvation), the total interaction energy, Δ*G* (Interaction), was obtained (Table 2). The total solvation energy (Δ*G* (Solvation)) indicated that complex formation was not a favorable event for both complexes (Table 2). However, the level of total gas phase molecular mechanics energy compensated for the disadvantageous solvation energy so that the bound state became more favorable compared to the unbound state in both of the complexes (Table 2).

For the polar component of the RBFE, while the BACH2 BTB domain monomer–peptide complex showed a disadvantageously increased polar contribution to the solvation energy (compared to the positive control complex, Δ*E* (EPB) was +447.8 kcal/mol higher), this was easily compensated by the electrostatic molecular mechanical energy (Δ*E* (EEL) was −550.5 kcal/mol lower compared to the positive control complex) (Table 2). Considering the total electrostatic contribution (Δ*E* (EEL) + Δ*E* (EPB)), a value of −9.0 kcal/mol was observed for the BACH2–peptide complex, whereas the positive control complex exhibited a completely disadvantageous level of +93.7 kcal/mol for this term (Table 2). This data demonstrated that the electrostatic contribution consisted of ∼36% of the total interaction energy in the BACH2–peptide complex, whereas it remained at a disadvantageous level for the positive control complex.

Nevertheless, the nonpolar component constituted the main energetic contribution driving the interaction between the partners in both the BACH2–peptide complex and the positive control complex BACH2–BACH2. The total nonpolar contribution (Δ*E* (VDWAALS) + (Δ*E* (ENPOLAR) + Δ*E* (EDISPER)) showed a significant increase in the positive control compared to the BACH2–peptide complex (−94.9 kcal/mol for the positive control complex and −16.4 kcal/mol for the BACH2–peptide complex) (Table 2). Despite this difference, the nonpolar contribution accounted for a large proportion (64%) of the total interaction energy in the BACH2–peptide complex, whereas in the positive control complex, it was only sufficient to shift the total interaction energy values to favorable negative values (Table 2). Overall, these results indicated that most of the affinity formed between the BACH2 BTB domain monomer and the wild-type (WT) peptide with a N-terminal cell-penetrating peptide extension arises from nonpolar interactions. This eventually prompted the need for further optimization of which at least some nonpolar residues should be replaced by polar ones so that increased specificity could be achieved in peptide binding. Therefore, in the final phase of the peptide design effort, possible amino acid replacements of peptide amino acids were predicted to generate a peptide library that could potentially carry the optimal number of hydrophilic/hydrophobic amino acids. The optimization process was completed using the FoldX 5.0 algorithm^45^ by substituting the amino acids of the predicted preliminary peptide in their native positions on the BACH2 BTB domain homodimer with other naturally occurring amino acids. At this point, this strategy was taken to select more realistic interactions for the amino acid replacements of the predicted peptide sequence within the homodimeric BTB domain of the BACH2 transcription factor. Because the MM-PBSA results reflected that nonpolar interactions contributed more heavily to the RBFE, to search for more specificity, plausible hydrophilic amino acid conversions were explored using FoldX (Tables 3 and 4). According to the summary of FoldX results in Table 3, a total of 4 hydrophilic amino acid replacements, showing statistically significant stabilizing impact for the monomer to monomer interaction of BACH2 BTB homodimer, were defined. Based on this output, a peptide library was generated by generation of all possible combinations of these mutations on the preliminary peptide (Table 4) which was fed into peptide structure prediction (Figure 3) and global docking studies.

**Table 3:** FoldX-Derived Interaction ΔΔ*G* Values for Hydrophilic Amino Acid Replacements, Predicted to Stabilize the Interaction of the BACH2 BTB Domain Homodimer

	selected chain A and B mutations			
#	mutation	AVG^a^	SD^b^	SEM^c^
1	PA10R^d^	–0.61	0.19	0.09
2	SA16E	–0.59	0.58	0.26
3	CA20H	–1.43	0.10	0.05
4	NB22R	–1.00	0.20	0.09

a ΔΔ*G,* average (calculated based on 5 replicates for each mutation).

b ΔΔ*G*, standard deviation.

c ΔΔ*G*, standard error of the mean.

d Example for the representation of the amino acid replacement: P (original amino acid) A (chain name) 10 (amino acid position on BACH2 BTB domain monomer) R (newly inserted amino acid).

**Table 4:** FoldX-Based Peptide Library That Was Directed to Structure Prediction and Global Docking Study

#	peptides utilized in the global docking
1	*YGRKKRRQRRRA* ^a^-PMYVYESTVHCTNIL^b^
2	*YGRKKRRQRRRA*-RMYVYESTVHCTNIL^c^
3	*YGRKKRRQRRRA*-PMYVYEETVHCTNIL
4	*YGRKKRRQRRRA*-PMYVYESTVHHNIL
5	*YGRKKRRQRRRA*-PMYVYESTVHCTRIL
6	*YGRKKRRQRRRA*-RMYVYEETVHCTNIL
7	*YGRKKRRQRRRA*-RMYVYESTVHHTNIL
8	*YGRKKRRQRRRA*-RMYVYESTVHCTRIL
9	*YGRKKRRQRRRA*-PMYVYEETVHHTNIL
10	*YGRKKRRQRRRA*-PMYVYESTVHHTRIL
11	*YGRKKRRQRRRA*-PMYVYEETVHCTRIL
12	*YGRKKRRQRRRA*-RMYVYEETVHHTNIL
13	*YGRKKRRQRRRA*-RMYVYEETVHCTRIL
14	*YGRKKRRQRRRA*-RMYVYESTVHHTRIL
15	*YGRKKRRQRRRA*-PMYVYEETVHHTRIL
16	*YGRKKRRQRRRA*-RMYVYEETVHHTRIL

a The italic sequence is the cell-penetrating peptide Tat 47–57, shown bound to N terminus of predicted peptide sequence. An alanine (A) was added to the C terminus of cell-penetrating peptide sequence in the case of a need for antibody detection.

b Preliminary predicted peptide sequence.

c Amino acid replacements gained from FoldX data are shown bold and underlined in the preliminary peptide sequence (an example R).

**Figure 3:**
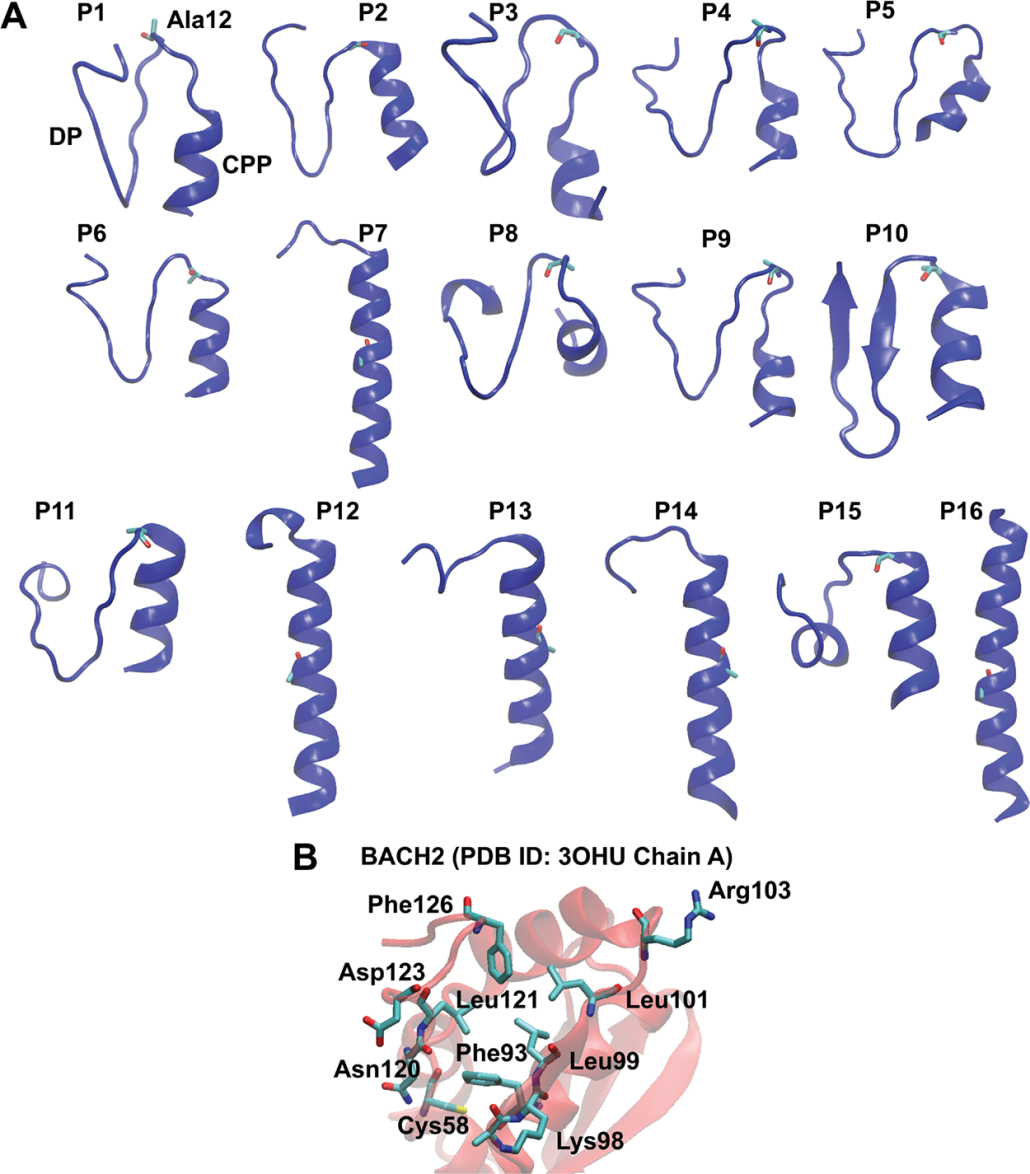
Predicted structures of the members of the peptide library and possible amino acid network governing dimerization for the BACH2 BTB domain. (A) Predictions were completed via PEPFOLD 3.5 program.^47–49^ The preliminary peptide region (designed peptide: DP) was displayed on the left of Ala12 for all peptides as Ala12 lies at the border to the cell-penetrating peptide (CPP) region (HIV Tat 47–57). The designed peptide regions for P1, P2, P3, P4, P5, P6, P9, and P11 remained unstructured. In the other peptides, the designed region had defined secondary structure formations. As also expected, the cell-penetrating peptide region has adopted a helical structure in all peptides (example naming: P1: peptide 1). (B) In addition to the amino acids Ala97, Lys98, Leu99, Leu100, Leu101, Ser102, and Arg103 identified in the preliminary peptide prediction with the Ligplot+ program (2), the BACH2 BTB domain homodimer was further analyzed using the PLIP program,^50^ and the amino acid network that may be crucial for interactions was expanded. This was done to broaden the region where peptides might bind, ensuring that possible peptide interactions were not overlooked.

### 3.2 Structure Prediction for the Peptide Library and Docking Studies

Peptide sequences in the peptide library shown in Table 4, which were generated through FOLDX program, were subjected to peptide structure prediction using the PEPFOLD 3.5 program^47–49^ (Figure 3A). In regard to the peptide structures obtained, peptides P10, P12, P13, P14, P15, and P16 displayed some secondary structure in their designed regions (DP), apart from the expected helical structure in the cell-penetrating peptide (CPP) region in the N terminal of all peptides (Figure 3A). As a result, it was concluded that as hydrophilic amino acid substitutions increase in the designed peptide region, the tendency of these regions to form secondary structure generally increased.

Next, in order to determine whether the predicted structures of 16 peptides target only the dimerization region of the BACH2 BTB domain, the global docking program ClusPro was utilized.^46^ Within this analysis, all peptide structures were globally docked to the BACH2 BTB domain monomer, and the resulting complexes were checked for whether peptides preferentially remain bound to the dimerization interface of the BACH2 BTB domain monomer. These preferences between peptides and target were evaluated through analyses of weak interactions, implemented via the PLIP program.^50^ As PLIP based weak interaction analyses dictated definition of amino acids residing in the interface, here PLIP program^50^ was also utilized to define interface residues from BACH2 BTB domain monomer (Figure 3B). For the BACH2 BTB domain, this approach expanded the set of amino acids for evaluating peptide interactions compared with initial consideration depicted in Figures 2 and S1. Amino acids identified through PLIP for BACH2 were given in detail within Figure 3B. After global docking via ClusPro, peptides capable of showing interactions with PLIP defined amino acid residues depicted in Figure 3B, as well as showing at least five hydrogen bonds or one salt bridge with the dimerization responsible amino acids, were selected as possible dimerization interfering peptides. With this selection criteria, importance of hydrophilic amino acid replacements in peptide selection was aimed to be underscored which may be in turn lead to selecting peptides that have more probable specificity toward BACH2 BTB domain. These peptides together with their host–guest interactions were listed in Table 5. During selection of peptide interactions, three amino acids upstream and downstream of these BACH2 BTB domain monomer target residues were also included in the analysis to ensure comprehensive targeting of the dimerization region.

**Table 5:** Weak Interaction Analyses of ClusPro-Based Global Docking Generated Structures of the Selected Peptides in Complex with the BACH2 BTB Domain Dimerization Region

peptide	interaction type	protein AA#^b^	peptide AA#	distance (Å)	side chain interaction
CP1^a^	hydrophobic	Ala57	Tyr17	3.79	
		Ala57	Thr20	3.45	
		Phe93	Ile26	3.48	
	hydrogen bond	Lys98	Asn25	1.64	yes
	salt bridge	Glu60	Arg19	3.47	
peptide 4	hydrogen bond	His119	Tyr17	3.63	yes
		Glu60	Thr20	2.95	yes
		Arg117	Val21	2.93	no
		Arg117	Val21	3.90	yes
		Arg117	Val21	2.67	no
	π-stacking	His119	Tyr17	5.26	
peptide 12	hydrophobic	Ala57	Tyr17	3.94	
	hydrogen bond	Arg117	Arg13	2.76	yes
		Arg117	Arg13	2.67	yes
	salt bridge	Glu60	Arg13	4.39	
peptide 13	hydrophobic	Ala56	Tyr17	3.90	
	salt bridge	Glu60	Arg13	3.56	
peptide 14	hydrogen bond	His119	Arg13	2.94	yes
	salt bridge	Glu60	Arg13	3.87	
peptide 15	hydrophobic	Ala97	Pro13	3.58	
		His119	Tyr15	3.75	
		Glu60	Tyr17	3.43	
		Ala57	Arg25	3.75	
	hydrogen bond	Asn120	Pro13	2.87	no
		His119	Tyr15	3.19	no
		His119	Tyr15	2.84	no
		Glu60	Tyr17	2.82	yes
		Glu60	Tyr17	2.82	yes

a CP1: control peptide 1.

b Amino acid residues residing +3 and −3 of the PLIP defined interaction surface shown in Figure 3B.

The results of the global docking program were summarized in Table 5. Key findings include: Control peptide 1 formed 3 hydrophobic bonds, 1 hydrogen bond, and 1 salt bridge, Peptide 4 formed 5 hydrogen bonds and 1 π-stacking interaction, Peptide 12 formed 1 hydrophobic interaction, 2 hydrogen bonds, and 1 salt bridge, Peptide 13 formed 1 hydrophobic interaction and 1 salt bridge, Peptide 14 formed 1 hydrogen bond and 1 salt bridge and Peptide 15 formed 4 hydrophobic interactions and 5 hydrogen bonds (Table 5). Based on the criteria set, peptides 1, 4, 12, 13, 14, and 15 were selected for further studies, while others were excluded due to insufficient levels of electrostatic interactions. These selected peptides and target BACH BTB domain monomer underwent local docking using the HADDOCK program^52^ to generate redocked MD ready structures and all these locally docked complexes were subjected to MD simulations with four replicates of 500 ns. The last 20 ns of these simulations, representing conformational stability, were used for MM-PBSA-based RBFE calculations of the peptide–protein complexes. In particular, local docking program HADDOCK was employed here to retrieve MD-ready structures since it allows flexibility to backbone and side chain of proteins.

### 3.3 MD Simulations of BACH2 BTB Domain–Peptide Complexes and Estimation of MM-PBSA-Based Relative Binding Free Energy

Next, HADDOCK redocked MD ready BACH2 BTB domain monomer–peptide complexes were subjected to 500 ns MD simulations in four replicates, totaling 2 μs of simulation time for each complex The conformational stability analysis for these MD simulations were completed through time-dependent RMSD analysis. During this step, BACH2 BTB domain homodimer was also subjected to MD simulations at similar time scale with protein–peptide complexes and was used as a positive control for protein–peptide interactions. In all simulation replicas, time intervals where the RMSD fluctuations remained below ∼3 Å were considered conformationally stable and deemed suitable for further in-depth analysis. Additionally, to allow for the evaluation of all simulations within the same time interval based on RMSD values, a common time interval showing stable conformations was selected. When all simulations were evaluated together (Supplementary Figures 4 and 5), most of the complexes, except those containing the BACH2 BTB domain-control peptide 1 (peptide 1, wild type) (Supplementary Figure 4B), BACH2 BTB domain–peptide 12 (Supplementary Figure 4D), and BACH2 BTB domain–peptide 14 (Supplementary Figure 4E), showed a continuous stable conformation in all 4 repeats of the 500 ns simulations. At this point, the BACH2 BTB domain–peptide 12 complex was eliminated from further analysis, since it did not show full conformational stability in two of the four replicates (Supplementary Figure 4D). The complexes containing BACH2 BTB domain-control peptide 1 (Supplementary Figure 4B) and BACH2 BTB domain–peptide 14 (Supplementary Figure 4E) showed conformational stability during the last 250 ns time interval in some of the replicas of the 500 ns MD simulations. Therefore, to analyze the common time interval showing conformational stability across all simulations, the last 250 ns section was selected.

After the time interval to be analyzed in common was determined, the last 20 ns of the last 250 ns of all MD simulation replicas for the protein–protein and protein–peptide complexes were used in estimation of RBFE with the MM-PBSA-method. During MM-PBSA studies, RBFE values between protein chains or protein–peptide chains were calculated (Figure 4). When MM-PBSA results were evaluated across all MD-simulated complexes in comparison with the positive control, the highest statistically significant difference was observed between the BACH2 BTB domain monomer and peptide 13 (Figure 4A, *p* < 0.0068, 51 kcal/mol higher RBFE compared with the positive control). Although other analyzed peptides also showed numerically favorable average RBFE values compared to the positive control, peptide 13 exhibited the highest computational affinity for the BACH2 BTB domain (Figure 4A). The control peptide 1 and peptide 4 were also more favorable than the positive control but did not reach statistical significance; therefore, peptide 4 was excluded from further analysis. However, the control peptide was retained for further studies as a potential lower affinity control, as it contains the wild type preliminary peptide sequence used in the beginning of the study. In line, the control peptide 1 sequence was also employed as an essential parameter for *in vitro* experimental setup. Furthermore, peptides 14 and 15 showed statistically significant differences compared to the positive control (*p* < 0.05), but due to their lower RBFE values and weaker statistical significance determined through *p* values, they were accepted as not reaching the computationally determined affinity level of peptide 13 and were excluded from deeper analyses at this point (Figure 4A). Overall, peptide 13 was selected as the peptide with the highest computationally determined affinity for binding to the BACH2 BTB domain monomer and was included in the *in vitro* studies.

**Figure 4:**
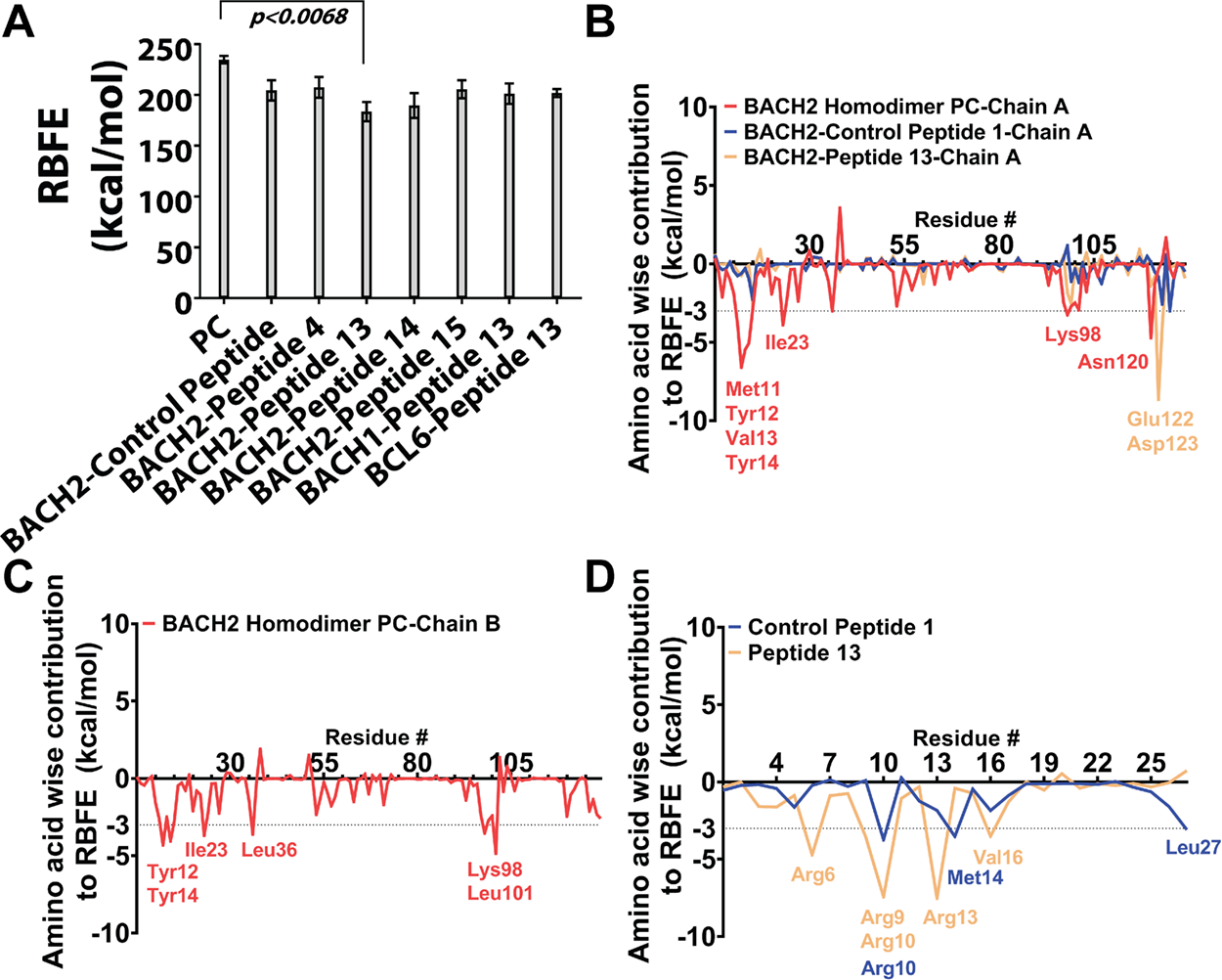
(A) Predicted relative binding free energies (RBFE) from MM-PBSA analysis of MD-simulated complexes and (B–D) amino acid-level contributions to RBFE for the positive control homodimer, BACH2 BTB domain monomer-control peptide (peptide 1), and BACH2 BTB domain monomer–peptide 13 complexes. Compared with the positive control (PC, BACH2 BTB homodimer, first bar), all BACH2 BTB monomer–peptide complexes exhibited higher RBFE values (numerically more negative values indicate an increase). Among these, the BACH2 BTB domain monomer–peptide 13 complex had the highest RBFE value (183.51 ± 9.61 kcal/mol (∼51 kcal/mol higher than PC on average) and the lowest *p*-value (*p* < 0.0068). Other RBFE values were PC (BACH2 BTB homodimer): 234.90 ± 3.75 kcal/mol, BACH2–control peptide (peptide 1): 204.57 ± 9.98 kcal/mol (∼30 kcal/mol higher than PC), BACH2-peptide 4:207.57 ± 10.28 kcal/mol (∼27 kcal/mol higher than PC), BACH2–peptide 14:189.67 ± 12.23 kcal/mol (∼45 kcal/mol higher than PC), and BACH2–peptide 15:205.68 ± 8.93 kcal/mol (∼29 kcal/mol higher than PC). Although peptides 14 and 15 also had statistically significant *p*-values (*p* < 0.05) compared with positive control, peptide 13 had the lowest *p*-value, making it the most suitable candidate for further *in vitro* studies. Additionally, peptide 13 showed the largest RBFE difference (∼50 kcal/mol higher than PC), indicating that it has the highest affinity toward the BTB domain. After identification of peptide 13 as the most plausible BACH BTB domain binder, its specificity toward the BACH BTB domain was tested using other BTB domain as negative controls. BACH1 BTB domain monomer-peptide 13 and BCL-6 BTB domain monomer–peptide 13 complexes were prepared similarly to other complexes using global docking followed by weak interaction analyses and local docking. These complexes were then simulated and subjected to estimation of RBFE using MM-PBSA. Obtained RBFE values were as follows: BACH1–peptide 13 complex: 201.29 ± 10.14 kcal/mol (∼17.7 kcal/mol lower than BACH2–peptide 13 complex), BCL-6–peptide 13 complex: 201.85 ± 4.07 kcal/mol (∼18.3 kcal/mol lower than BACH2–peptide 13). Although no statistically significant difference was found compared with the BACH2–peptide 13 complex, the lower RBFE values suggested that peptide 13 may be selective for the BACH2 BTB domain monomer. Among these negative controls, the BACH1 BTB domain monomer was selected for *in vitro* studies, as it shares 91% sequence similarity and 59% identity with the BACH2 BTB domain monomer over 138 amino acids.^72^ All RBFE values and amino acid contributions were averaged from the last 20 ns of four separate 500 ns simulations. RBFE values were reported as the mean ± standard error of the mean (SEM). *t* test (*p* < 0.05) was used to determine statistically significant differences from PC. (B) Amino acid wise contributions to RBFE in the target BACH2 BTB domain of the protein–peptide complexes were shown. Amino acids contributing more than −3 kcal/mol were considered as significant contributors. In the target monomer of the PC BACH2 BTB domain homodimer, Met11, Tyr12, Val13, Tyr14, Ile23, Lys98, and Asn120 showed the highest contributions, with Met11-Tyr14 and Asn120 being the most dominant. In the target monomer of BACH2 BTB domain monomer–peptide 1 complex, no amino acid exceeded −3 kcal/mol. In the target monomer of BACH2 BTB domain monomer–peptide 13 complex, Glu122 and Asp123 contributed significantly, aligning with the positive control’s N terminal interaction pattern. (C) Amino acid wise contributions to RBFE in the other monomer of PC BACH2 BTB Domain Homodimer (Chain B) was shown. This monomer utilized Tyr12, Tyr14, Ile23, Leu36, Lys98, and Leu101 as determined through levels exceeding −3 kcal/mol. These contributors both aligned with the other monomer‘s contributors shown in (B), as well as validated the initial preliminary peptide prediction shown in Table 1. (D) Peptide-level amino acid contributions to RBFE are depicted for control peptides 1 and 13. Control peptide 1 contributed through Arg10 on the cell-penetrating region and Met14 and Leu27 on the designed region. Peptide 13 contributed via Arg6, Arg9, and Arg10 in the cell-penetrating region and Arg13 and Val16 in the design-exposed region. Similar to protein contributions, only amino acids with RBFE contributions of less than −3 kcal/mol were included. RBFE contributions were calculated using MM-PBSA.py with idecomp =1 to determine amino acid-level interactions.

Since peptide 13 was selected, as the next step, computational specificity of peptide 13 was also assessed through the same pipeline, now with BACH1 BTB domain monomer and BCL-6 BTB domain monomer (Supplementary Figures 3A,B, 5B,C, and Figure 4A). As completed with BACH2 BTB monomer, first, BACH1 BTB domain dimer (PDB ID: 2IHC) and the BCL-6 BTB domain dimer (PDB ID: 6CQ1) was retrieved from PDB and checked for residues that mediate the weak interactions between monomers using PLIP program. Second, peptide 13 was globally docked to BACH1 BTB domain and BCL-6 BTB domain monomers using the ClusPro program, which returned possible binding of peptide 13 to these domain as well. As presented in Supplementary Table 1, PLIP based weak interaction analyses of ClusPro global docking, peptide 13 was shown to be potentially capable of binding to the dimerization mediating regions in BACH1 and BCL-6 BTB domain monomers. Next using this amino acid information gained from PLIP, BACH1 BTB domain monomer-peptide 13 and BCL-6 BTB domain monomer–peptide 13 structures were redocked using HADDOCK local docking and subjected to 500 ns MD simulations in four replicates. Convergence of conformational stability for these specificity control simulations was analyzed using RMSD analysis (Supplementary Figure 5B,C). For both complexes, all simulation replicates were stable within 20 ns. In parallel to this observation, last 20 ns of last 250 ns of all specificity controls were subjected to MM-PBSA based estimation of RBFE values and were compared with that of BACH2 BTB domain monomer–peptide 13 (Figure 4A). It was clear that peptide 13 exhibited an approximately 18 kcal lower RBFE in both BTB domains. While these results suggest a major advantage in terms of selectivity of peptide 13 toward BACH2 BTB domain, BACH1 BTB domain monomer was selected to be included in the *in vitro* part of the study since it shares high sequence and structural similarity with BACH2 BTB domain monomer.

After identifying the highest-affinity peptide 13 toward BACH2 BTB domain monomer, the other potential negative control peptide (control peptide (peptide 1) as a low affinity peptide toward BACH2 BTB domain monomer) and negative control target protein (BACH1 BTB domain monomer as a possible low affinity target for peptide 13) of all which were intended to be employed in *in vitro* studies, a detailed analyses was conducted on the amino acid-level contributions to the RBFE values in positive control complex BACH2 BTB domain homodimer, BACH2 BTB domain-control peptide 1 complex and BACH2 BTB domain–peptide 13 containing complexes (Figure 4B–D). When positive control BACH2 BTB homodimer was examined, it was found that amino acids Met11, Tyr12, Val13, Tyr14, Ile23, Lys98, and Asn120 on chain A, and Tyr12, Tyr14, Ile23, Leu36, Lys98, and Leu101 on chain B made the highest contributions to the positive control RBFE value (Figure 4B,C). When these amino acids were comparatively evaluated with those predicted for the preliminary peptide (Figure 2 and Table[Table tbl1]), it was apperant that initial predicted peptide sequence, peptide binding site and also the related docking region used for peptide binding were validated. (Chain A forms an interaction network with amino acids 98 and 120, while chain B interacts with amino acids 12, 14, and 23. The amino acids identified on chain B are part of the control peptide, whereas those identified on chain A correspond to the region where the control peptide is expected to dock.) Interestingly, while the BACH2 BTB domain monomer of control peptide 1 returned no contributions higher than the −3 kcal/mol threshold, verifying its low affinity, peptide 13 interacted with the BACH2 BTB domain through its C terminal residues Glu122 and Asp123 (Figure 4B). Additionally, when the amino acid contributions within the RBFE of peptide 13 against the BACH2 BTB domain homodimer were assessed, it was observed that it contributed via Arg6, Arg9, and Arg10 in the cell-penetrating peptide region and via Arg13 and Val16 in the designed region (Figure 4C). In contrast, the control peptide (peptide 1) contributed via Arg10 in the cell-penetrating peptide region and via Met14 and Leu27 in the designed region. Given both the lower number of contributing amino acids and their individually weaker RBFE contribution values, this analysis explained why the control peptide exhibited lower affinity compared with peptide 13.

In the next stage, the evaluation of the components of RBFE obtained from the MM-PBSA-based calculations was provided. Within these components: (1) the total gas-phase molecular mechanical energy, Δ*G* (Gas), was obtained by summing Δ*E* (VDWAALS) and Δ*E* (EEL); (2) the total solvation energy, Δ*G* (solvation), was obtained by summing Δ*E* (EPB), Δ*E* (ENPOLAR), and Δ*E* (EDISPER); (3) the total interaction energy, Δ*G* (interaction) excluding the entropic term, was obtained by summing Δ*G* (gas) and Δ*G* (solvation); (4) the RBFE was defined by subtracting the entropic term from Δ*G* (interaction) (Table 6). The total solvation energy (Δ*G* (solvation)) values indicated that complex formation is not favorable for any of the three complexes (Table 6). However, the total gas-phase molecular mechanical energy levels observed in all complexes showed that the bound complex state is preferred over the unbound state (Table 6). When the polar contribution (Δ*E* (EEL) + Δ*E* (EPB)) was analyzed, it was found that this contribution was completely disadvantageous for the positive control (PC) homodimer (+83.2 kcal/mol) and the complex containing peptide 1 (+12.4 kcal/mol) (Table 6). However, favorably compatible with the insertion of hydrophilic amino acids in the sequence, the total electrostatic contribution in the complex containing peptide 13 (−23.8 kcal/mol) constituted approximately 81.5% of the interaction energy excluding the entropic term (Δ*E* (interaction) excluding *T*Δ*S*) (Table 6). At this point, it was concluded that the goal of increasing hydrophilic interactions for peptide 13 was successfully achieved.

**Table 6:** Components of RBFE for BACH2 BTB Domain Monomer–Peptide Complexes (Control Peptides 1 and 13) and Positive Control Homodimeric BACH2 BTB Domain Complex^a^

	PC		BACH2–peptide 1		BACH2–peptide 13	
(kcal/mol)	Mean	SEM	Mean	SEM	Mean	SEM
Δ*E* (VDWAALS)	–244.2	5.3	–53.9	4.1	–39.8	3.1
Δ*E* (EEL)	–386.1	17.9	–291.8	80.7	–916.6	98.3
Δ*E* (EPB)	469.3	15.4	304.2	74.2	892.8	87.4
Δ*E* (ENPOLAR)	–191.9	2.9	–47.5	4.9	–45.3	4.8
Δ*E* (EDISPER)	338.6	6.0	86.3	7.0	79.8	7.7
Δ*G* (gas)	–630.3	13.8	–345.7	83.0	–956.4	99.8
Δ*G* (solvation)	616.1	13.1	342.9	75.9	927.2	89.8
TAS	–249.1	0.8	–207.4	3.1	–210.0	1.1
Δ*G* (interaction) excluding TAS	–14.2	4.0	–2.8	7.3	–29.2	10.7
Δ*G* (interaction) including TAS	234.9	3.7	204.6	10.0	180.8*	9.6

a Δ*E* (VDWAALS): van der Waals molecular mechanics energy, Δ*E* (EEL): electrostatic molecular mechanics energy, Δ*E* (EPB): polar contribution to solvation energy, Δ*E* (ENPOLAR): nonpolar contribution to solvation energy from repulsive solute–solvent interactions, Δ*E* (EDISPER): nonpolar contribution to solvation energy from attractive solute–solvent interactions, Δ*G* (gas): total gas phase molecular mechanics energy, Δ*G* (solvation): total solvation energy, TAS: entropic term, Δ*G* (interaction): total RBFE, PC: positive control complex, SEM: standard error of the mean, (all complexes were calculated with 4 replicates) *p < 0.0068 compared with PC.

When the total nonpolar contribution (Δ*E* (VDWAALS) + Δ*E* (ENPOLAR) + Δ*E* (EDISPER)) was examined, the PC homodimer complex compensated for the disadvantageous state observed in the polar contribution with a level of −97.5 kcal/mol, maintaining the interaction energy excluding the entropic term (Δ*G* (Interaction) Excluding *T*Δ*S*) at approximately −14 kcal/mol (Table 6). A similar pattern was valid for the complex containing control peptide 1, where the nonpolar contribution (−15.1 kcal/mol) compensated for the polar contribution, bringing the interaction energy excluding the entropic term (Δ*G* (Interaction) Excluding *T*Δ*S*) to approximately −3 kcal/mol (Table 6). Thus, for PC and the complex containing control peptide 1, the nonpolar contribution resolved the disadvantageous binding contribution of the polar term, keeping the enthalpic term at a level that supports binding. However, the nonpolar contribution remained at −5.3 kcal/mol in the complex containing peptide 13, which showed the highest affinity, and together with the polar term, it helped support the enthalpic term for binding (Table 6). The nonpolar contribution constituted approximately 18% of the total interaction energy excluding the entropic term (Δ*G* (Interaction) excluding *T*Δ*S*) in the complex containing peptide 13. These results underscored our findings in the polar contribution that peptide 13 aimed to engage in more hydrophilic interactions.

Finally, the entropic term (*T*Δ*S*) of which was computed through quasi-harmonic approximation was evaluated. As shown in Table 6, the entropic term calculated for the interaction energy in all complexes had excessively negative values. This motif primarily shifted the RBFE calculated with the entropic term (Δ*G* (interaction) (including *T*Δ*S*)) to unrealistic positive values, disproportionately compensating for the total enthalpic term (Δ*G* (interaction) excluding *T*Δ*S*) (Table 6). Deviation from realistic values was considered acceptable both because it is an expected outcome in the quasi-harmonic approximation of the entropic term^65^ and because only the ranking, not the absolute value of the RBFE was utilized in our study. Additionally, since there were no studies in the literature on the absolute binding energy of the PC BACH2 homodimer, an optimization study was deemed not possible, and the study proceeded with the calculated entropic term values. In light of this background, when the entropic term values were considered, it was noted that the entropic penalty in the PC complex decreased in the peptide-containing complexes (Table 6). This indicated that the interaction in the peptide-containing complexes, due to containing a peptide instead of a larger BACH2 monomer, had a more flexible binding mode, as expected. When the control peptide was examined, although this complex showed a lower enthalpic term (Δ*G* (interaction) excluding *T*Δ*S* −2.8 kcal/mol—PC −14.2 kcal/mol) compared with the PC, it surpassed the PC in RBFE (Δ*G* (interaction) (including *T*Δ*S*)) due to the decrease in the entropic penalty (Table 6). However, this effect observed in peptide 1 did not cause a statistically significant difference with the PC. Peptide 13, on the other hand, showed an interaction energy of approximately +180 kcal/mol with higher enthalpic contribution and decreased entropic penalty (Table 6). This RBFE value of peptide 13 generated a more favorable binding ranking by approximately 54 kcal/mol compared with that of the PC and approximately 24 kcal/mol compared with that of the control peptide 1 (Table 6). Ultimately, peptide 13 emerged as the most advantageous peptide in terms of statistical significance and ranking, with a lower entropic penalty (−39 kcal/mol) and a higher enthalpic value (−15 kcal/mol) compared with the PC complex (Table 6).

### 3.4 In-Depth Analyses of MD Simulations

As depicted in Figure 5A, the amino acids contributing to the MM-PBSA-based RBFE were identified for the PC complex, the BACH2 BTB domain monomer (Chain A)–control peptide 1 complex, and the BACH2 BTB domain monomer (Chain A) control peptide 13 complex. From this point onward, in depth analyses of MD simulations were conducted within the framework of these amino acids, focusing their contribution in the conformationally stable common time frame, last 250 ns (Figure 5A).

**Figure 5:**
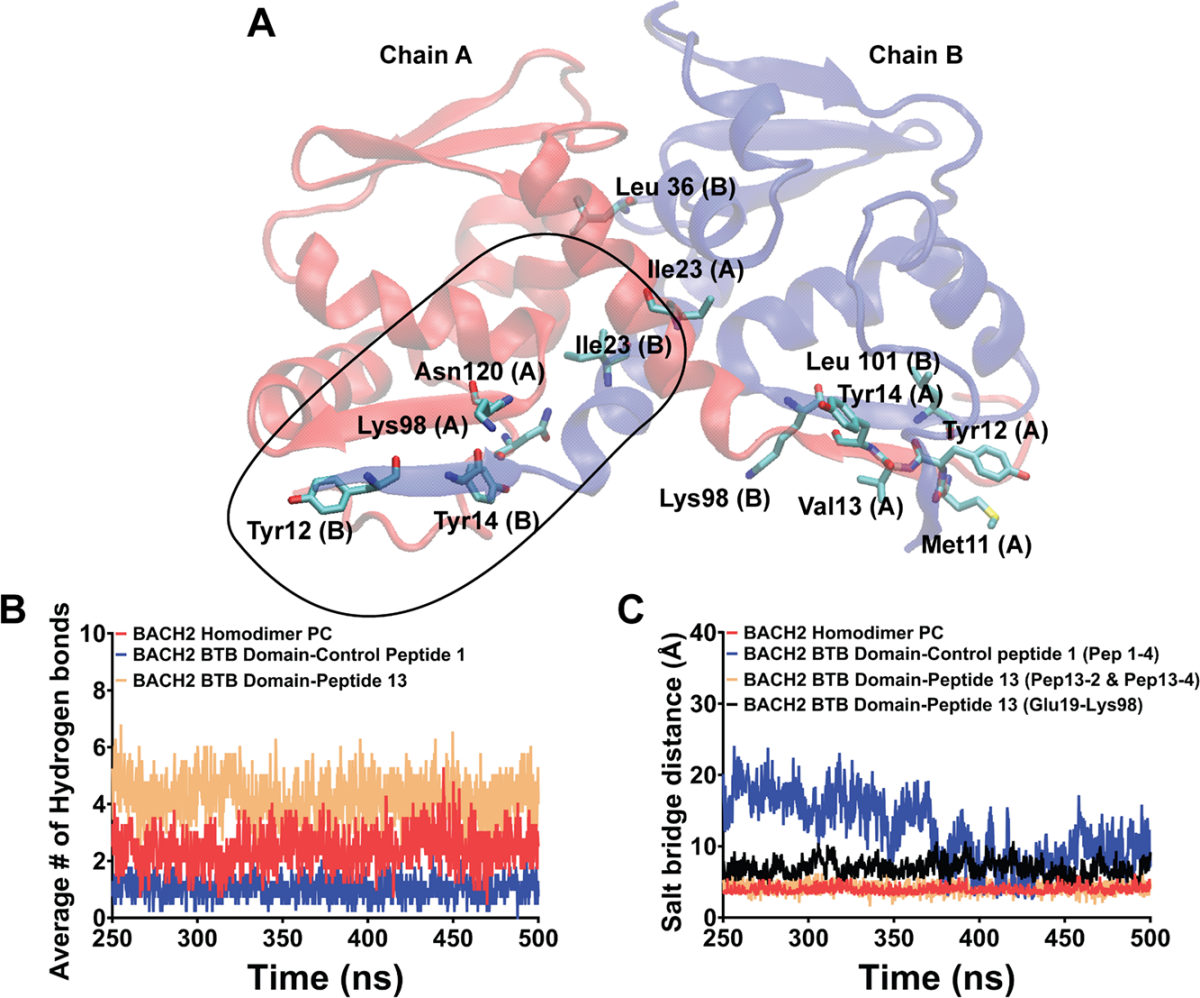
Visualization of contributing amino acids to the RBFE and analyses of electrostatic interactions through hydrogen bonds and salt bridges. (A) Amino acids that contribute the most to the RBFE obtained from the PC complex simulations, determined using the MM-PBSA method, were represented on the homodimer of the BACH2 BTB domain. The region enclosed within the black ellipse indicates the area where the control peptide, predicted from chain B, is located and the corresponding interacting region in chain A. In the complex containing the control peptide, no amino acids on the BACH2 BTB domain monomer (chain A) were found to contribute to the interaction lower than the threshold −3 kcal/mol. However, in the complex containing peptide 13, Glu122 and Asp123 on the BACH2 BTB domain monomer were identified as the amino acids contributing to the interaction (not shown). In addition to these residues, the amino acids Arg10, Met14, and Leu27 on the control peptide 1, and Arg6, Arg9, Arg10, Arg13, and Val16 on peptide 13, which exhibits the highest affinity for the BACH2 BTB domain, have been identified as the key contributors to the peptide’s affinity for BACH2 BTB domain (chain A) (not shown). (B) Average number of unique hydrogen bonds formed by the side-chain atoms of PC the BACH2 BTB domain homodimer, the BACH2 BTB domain monomer–control peptide 1 complex, and the BACH2 BTB domain monomer-peptide 13 complex were presented within the last 250 ns of simulations. For the PC, hydrogen bonds between the two monomers were considered, whereas for peptide-containing complexes, hydrogen bonds between the peptide and one BACH2 BTB domain monomer were counted. The average number of hydrogen bonds formed by the side-chain atoms of amino acids that hold the complexes together were presented frame-wise. The average number of hydrogen bonds is given as the mean of four replicates. Peptide 13 exhibited a higher number of hydrogen bonds compared to both control peptide 1 and the PC homodimer. These results supported the conclusion that peptide 13 targets the BACH2 BTB domain homodimer with higher electrostatic contribution than either of the other complexes (approximate average number of hydrogen bonds: Peptide 13 ∼ 5, PC ∼ 3, Control Peptide ∼ 1). (C) Distance comparison of the salt bridge detected in the PC BACH2 BTB domain homodimer between Lys98 (Chain A) and Glu5 (Chain B) with its counterpart salt bridges in the BACH2 BTB domain monomer bound to control peptide 1 and peptide 13. The distance was measured from the center of mass of the acidic side-chain oxygen to the center of mass of the basic side-chain nitrogen. A salt bridge was defined when the distance dropped below 3.2 Å in at least one frame. All analyses were completed within the last 250 ns of the simulations, where common conformational stability was observed. In the PC complex, a salt bridge was detected (marked in red) between Lys98 in Chain A of the BACH2 BTB domain homodimer and Glu15 from the predicted peptide-binding region on Chain B. The average distance of this salt bridge remained ∼3.5 Å over the last 250 ns of four simulation replicates (red). Given the identicality or close proximity of Lys98 and Glu5 to amino acids contributing to the RBFE in MM-PBSA studies, the stability of this salt bridge in the last 250 ns of the simulation was considered an important affinity-enhancing parameter for the PC. In this context, the presence of equivalent salt bridges was investigated in the complexes containing the control peptide 1 and peptide 13. In the BACH2 BTB domain monomer–control peptide 1 complex, the equivalent salt bridge was observed between Lys98 (Chain A)-Glu18 (Chain B-Peptide) (blue). However, this salt bridge was only observed in a single simulation replicate (Pep1–4, blue). Furthermore, when the distance of this salt bridge was analyzed over the last 250 ns of the simulation, it was found to remain mostly above 10 Å. In the complex containing peptide 13, this salt bridge (Lys98-Glu18) was observed in two replicates (Pep13-2 and Pep13-4), with an average distance (∼3–5 Å) similar to the PC (beige). The fact that peptide 13 exhibited a distance closer to the PC for this critical salt bridge highlighted its superiority over the control peptide. Additionally, in the initial peptide design stage, a Ser19Glu mutation was introduced in peptide 13 using the FoldX method, adding an extra GLU19 residue adjacent to Glu18. It was observed that LYS98 in the BACH2 monomer also formed a salt bridge with this modified Glu19 in all four simulation replicates, analogous to the PC (black). Distance analysis showed that although this salt bridge fluctuated between ∼5–9 Å, its presence in at least one frame of all peptide 13 containing simulation replicates suggested that it could serve as an alternative to the Lys98-Glu18 salt bridge. All of the analyses for hydrogen bonds and salt bridges were performed using the Salt Bridges plugin embedded in the VMD program.

At the initial phase of extensive analysis MD simulations, hydrogen bonding patterns were deciphered in the complexes within the last 250 ns. As shown in the Table 7, involvement of MM-PBSA defined residues and numerical magnitude of occupancy values for hydrogen bonding observed between PC monomers and between BACH2 BTB domain monomer and peptide 13 were mostly higher and much more uniform in all simulation replicates, compared with control peptide 1 containing complex. This impact of hydrogen bonds were more clear when a comparison based on the average number of side-chain-associated hydrogen bonds that hold the complexes together were assessed (Figure 5B). Peptide 13 exhibited approximately 1.5 times more hydrogen bonds than PC and about 5 times more hydrogen bonds than the control peptide in terms of the average number of side-chain-associated hydrogen bonds stabilizing the complexes during the last 250 ns of the simulations (Figure 5B). Ultimately, these results were considered to validate both FoldX defined hydrophilic amino acids insertion as well as, the dependence of the MM-PBSA derived relative binding free energies mostly on electrostatic contributions for peptide 13 containing complex.

**Table 7:** Unique and Highly Occupied Hydrogen Bonds Formed between Side Chain Atoms of Monomers or Monomer and Peptide in BACH2 BTB Domain Homodimer (PC), BACH2 BTB Domain Monomer–Control Peptide 1 (CP1), and BACH2 BTB Domain Monomer–Peptide 13 (P13) Complexes^a^

don.	acc.	occ. (%)	don.	acc.	occ. (%)	don.	acc.	occ. (%)
PC-1			CP1-1			P13-1		
**K98**	**E15**	**53.2**	**R10P**	**E15**	**52.8**	**R10P**	**D123**	**39.3**
**N22**	**N120**	**52.8**	**R10P**	**E15**	**49.9**	**R10P**	**D123**	**37.2**
**K98**	**E15**	**48.3**	K5P	D123	28.9	**R10P**	**D123**	**32.0**
**N120**	**H19**	**20.8**	K5P	D123	24.9	**R10P**	**D123**	**30.5**
PC-2			CP1-2			P13-2		
**N22**	**N120**	**57.4**	**S124**	**T24P**	**33.4**	**R13P**	**E122**	**31.9**
**K98**	**E15**	**49.7**	**R10P**	**E15**	**13.5**	**R13P**	**E122**	**24.6**
**K98**	**E15**	**47.2**	**R10P**	**E15**	**12.8**	**K98**	**E18P**	**20.8**
**Y14**	**E122**	**13.0**	**Y15P**	**E15**	**11.8**	**K98**	**E18P**	**19.2**
PC-3			CP1-3			P13-3		
**K98**	**E15**	**48.1**	K5P	E15	14.2	**R13P**	**D123**	**37.0**
**K98**	**E15**	**47.0**	**R7P**	**E15**	**13.4**	**Y15P**	**T21**	**37.3**
**N22**	**N120**	**47.0**	**R7P**	**E15**	**11.8**	**R9P**	**E122**	**36.4**
**N120**	**H19**	**26.0**	K5P	E15	9.1	**T17**	**T24P**	**36.0**
PC-4			CP1-4			P13-4		
**N22**	**N120**	**58.4**	K5P	D123	14.1	**R10P**	**D123**	**44.0**
**K98**	**E15**	**56.6**	K5P	D123	13.0	**Y17P**	**D123**	**38.8**
**K98**	**E15**	**52.3**	T20P	E15	6.2	**R10P**	**D123**	**30.1**
**N120**	**H19**	**33.9**	T20P	E15	5.9	**R13P**	**N120**	**29.0**

a H-bond analyses were performed for each simulation replicate. Bold indicates involvement of MM-PBSA selected amino acids (for the selection criteria of hydrogen bonds mediated by MM-PBSA defined residues, presence of any MM-PBSA defined amino acid as a hydrogen bond donor or acceptor was used for PC. For peptide containing complexes, the presence of MM-PBSA defined amino acids from peptide side was considered as selection criteria. +3 and −3 positions relative to the contributing amino acids in the sequence were also considered). The first four hydrogen bonds in the ranking were selected and presented based on the occupancy rate. Don: donor, Acc: acceptor, Occ: occupancy. In donor and acceptor columns, the first letter stands for one letter amino acid abbreviation, numerical values indicate residue number. P (Ex: R10P) indicates the peptide side amino acid.

One step further, salt bridge analyses were performed for the PC complex as well as for the BACH2 BTB domain complexes containing the control peptide and peptide 13 (Figure 5C). Similar to the hydrogen bonding, this analyses allowed us to observe the possible contribution of salt bridges to protein-peptide affinity within the scope of the amino acids contributing to MM-PBSA analysis. In this context, in the PC, when examined in terms of the amino acids contributing to MM-PBSA-based affinity as defined earlier (Figure 5A), a salt bridge was detected between Lys98 (BACH2 BTB Domain Chain A) and Glu15 (BACH2 BTB Domain Chain B) in every simulation replicate (Figure 5C). The fact that the LYS98-GLU15 bond was also predicted in the hydrogen bond analysis supported its significance (Table 7). Since Tyr14 in Chain B was one of the amino acids contributing to RBFE, Glu15, located at its +1 position, was also included in the salt bridge analysis (Figure 5A). In the complex containing peptide 1, equivalent of this LYS98-GLU15 salt bridge was identified as LYS98-GLU18. However, since this salt bridge was observed in only one replicate and showed very large distance fluctuations, it was considered unlikely to contribute strongly to affinity (Figure 5C). In peptide 13, on the other hand, the Lys98-Glu18 salt bridge was detected in two replicates and exhibited a distance comparable to that of its counterpart in the PC peptide (Figure 5C). This fact further positioned peptide 13 as a more favorable candidate compared to peptide 1. Additionally, an engineered Glu19 residue in peptide 13 formed a salt bridge with Lys98 of the BACH2 BTB domain monomer in all four simulation replicates (Figure 5C). Although the average distance of this Lys98-Glu19 salt bridge was higher than that of the identified salt bridge of the PC complex, it was hypothesized that this salt bridge might serve as an alternative to the Lys98-Glu18 salt bridge, which was already observed in two replicates of peptide 13 (Figure 5C). As a result, when the amino acids contributing to RBFE in the MM-PBSA analysis were evaluated in the context of the established salt bridges, it was observed that peptide 13 attempted to form a network similar to the salt bridges found in the PC. In contrast, the low-affinity peptide 1 exhibited these relevant salt bridges at a much more limited level.

In the next stage, a carbon–carbon contact analysis was conducted in parallel with the amino acid contribution data predicted by the MM-PBSA-based RBFE analysis (Supplementary Table 2). This allowed for the assessment of potential hydrophobic interactions within the final 250 ns of simulations, focusing on the PC BACH2 homodimer complex, the complex containing peptide 1, and the complex containing peptide 13 (Supplementary Table 2). A carbon–carbon contact occupancy threshold of 50% was used as the cutoff criterion for selecting significant interactions (Supplementary Table 2). In the PC BACH2 dimer complex, carbon–carbon interactions were examined between Met11, Tyr12, Val13, Tyr14, Ile23, Leu36, Lys98, and Asn120 in chain A and Tyr12, Tyr14, Ile23, Leu36, Lys98, and Leu101 in chain B during the final 250 ns of simulation replicas (Supplementary Table 2). In this PC complex, interactions such as Tyr12–Leu101, Tyr14–Lys98, Lys98–Tyr14, and Asn120–Tyr14 were found to be present for at least 90% of the last 250 ns, indicating the persistence of hydrophobic contacts (Supplementary Table 2). For the carbon–carbon contact analysis of the peptide 1-containing complex, amino acids in chain A of the PC that contributed to the MM-PBSA RBFE were considered alongside Arg10, Met14, and Leu27 from the peptide side, which contributed to binding in the peptide 1 complex (Supplementary Table 2). Since no MM-PBSA-contributing residues were identified in the BACH2 BTB domain of the peptide 1 complex, only the contributing residues from the PC’s chain A monomer were included in the analysis. In this context, MM-PBSA-defined residue Met11 from the BACH2 BTB domain chain A showed carbon–carbon contacts with Arg10 (Pep1-1 replica, 54.2% occupancy) and Met14 (Pep1-4 replica, 56% occupancy), both exceeding the 50% threshold (Supplementary Table 2). Additionally, within the peptide 1 complex, Lys98–Met14 (1 replica, 90.6% occupancy) and Val13–Arg10 (1 replica, 99.4% occupancy) emerged as key potential hydrophobic interactions based on their occupancy rates (Supplementary Table 2). For the peptide 13 containing complex, the same amino acids from the PC chain A monomer were analyzed along with the peptide-side contributors Arg6, Arg9, Arg10, Arg13, and Val16 from the MM-PBSA analysis of the peptide 13 complex (Supplementary Table 2). To ensure comparability with the control peptide 1, MM-PBSA-defined residues from the BACH2 BTB domain in the PC were included in the hydrophobic contact analyses of peptide 13 containing comples. In the peptide 13 containing complex, carbon–carbon interactions such as Asn120–Arg13 (3 replicas), Tyr12–Arg9 (1 replica), Asn120–Val16 (3 replicas), and Lys98–Val16 (4 replicas) demonstrated occupancy rates of at least 80% (Supplementary Table 2). Based on these results, considering the number of carbon–carbon contacts, their specificity to residues identified in the PC, and their occupancy rates, the complex containing peptide 13 was found to exhibit a higher hydrophobic interaction potential compared with the control peptide 1 complex, at least within the MM-PBSA defined residues, and showed greater similarity to the PC. Overall, these findings were considered to support the higher binding affinity of peptide 13 toward BACH2.

Beyond the previously described favorable features of peptide 13 associated with persistence of weak interactions of MM-PBSA defined residues in the final half of 500 ns simulations, the subsequent phase of analysis further investigated the consistency of differences observed in relative binding energy between peptide-containing complexes using RMSF-based backbone α carbon flexibility, interatomic distance, and solvent-accessible surface area (SASA) analyses. Initially, α carbon flexibility of the target BACH2 BTB domain monomer (chain A) was analyzed based on RMSF values across the homodimer PC, the control peptide (peptide 1), and the peptide 13-containing complexes (Figure 6A). As the RMSF quantifies atomic flexibility, reductions in this value were interpreted as indicative of tighter binding. The results revealed that N- and C-terminal flexibility of the BACH2 BTB domain monomer (chain A) was more similar between the PC and the peptide 13 complex, while the control peptide complex exhibited higher flexibility in these regions (Figure 6A). This positive control-like rigidity in the BACH2 BTB monomer within the peptide 13 complex was also reflected in the peptide itself (Figure 6B). Notably, residues 6–19 of peptide 13 displayed lower α carbon flexibility during the final 250 ns compared to the corresponding region of the control peptide (Figure 6B). These flexibility data, derived from the final segment of the simulations, were interpreted as supporting the higher binding affinity of peptide 13. To further support these findings, distance analysis was subsequently performed as a confirmatory step (Figure 6C). A reduction in distance between interacting molecules was considered a sign of stronger binding. Accordingly, residues contributing to the MM-PBSA-derived RBFE of peptide 13 were analyzed in both the control peptide and peptide 13 complexes (Figure 6C). In the PC homodimer, the distance between these residues on chain A and the region on chain B corresponding to the predicted peptide binding site served as a reference. Peptide 13 and PC produced comparable results within this distance range, while the control peptide complex exhibited substantially greater distances. Thus, distance analysis further demonstrated that peptide 13 behaved similarly to chain B of the PC homodimer in its interaction with the BACH2 BTB domain monomer. Finally, SASA analysis was conducted on chain A of all complexes to validate the distance data obtained for peptide 13 (Figure 6D). A decrease in SASA was interpreted as indicative of stronger binding due to reduced solvent exposure. Results from this analysis showed that the BACH2 BTB domain monomer in the peptide 13-containing complex generally exhibited a SASA pattern more closely resembling that of PC during the majority of the final 250 ns of simulation (Figure 6D). This behavior was interpreted as an attempt by peptide 13 trying to mimic the interaction mode of chain B in the PC homodimer. In contrast, the control peptide complex maintained a higher SASA value throughout the final 250 ns, with chain A consistently displaying greater solvent exposure compared with the peptide 13 complex (Figure 6D). In the next phase, the relationship between the all-atom flexibility of specific key peptide residues and binding behavior was examined over the final 250 ns of the simulations. To this end, two amino acids of peptide 13—Arg9, located in the cell-penetrating segment and contributing to the RBFE as defined earlier, and Val16, contributing from the designable region—were subjected to RMSF-based all-atom flexibility analysis within both the control peptide and peptide 13 complexes (Figure 6E,F). The results revealed that Arg9 exhibited significantly lower flexibility in the peptide 13 complex compared to the control peptide (Figure 6E,G), while Val16 showed a similar trend toward reduced flexibility that approached, but did not reach, statistical significance (Figure 6F,H). Overall, all of these findings supported the MM-PBSA-based binding energy data and suggested that the RBFE observed in the final 20 ns of simulation may, in fact, be sustained over the broader 250 ns simulation window.

**Figure 6:**
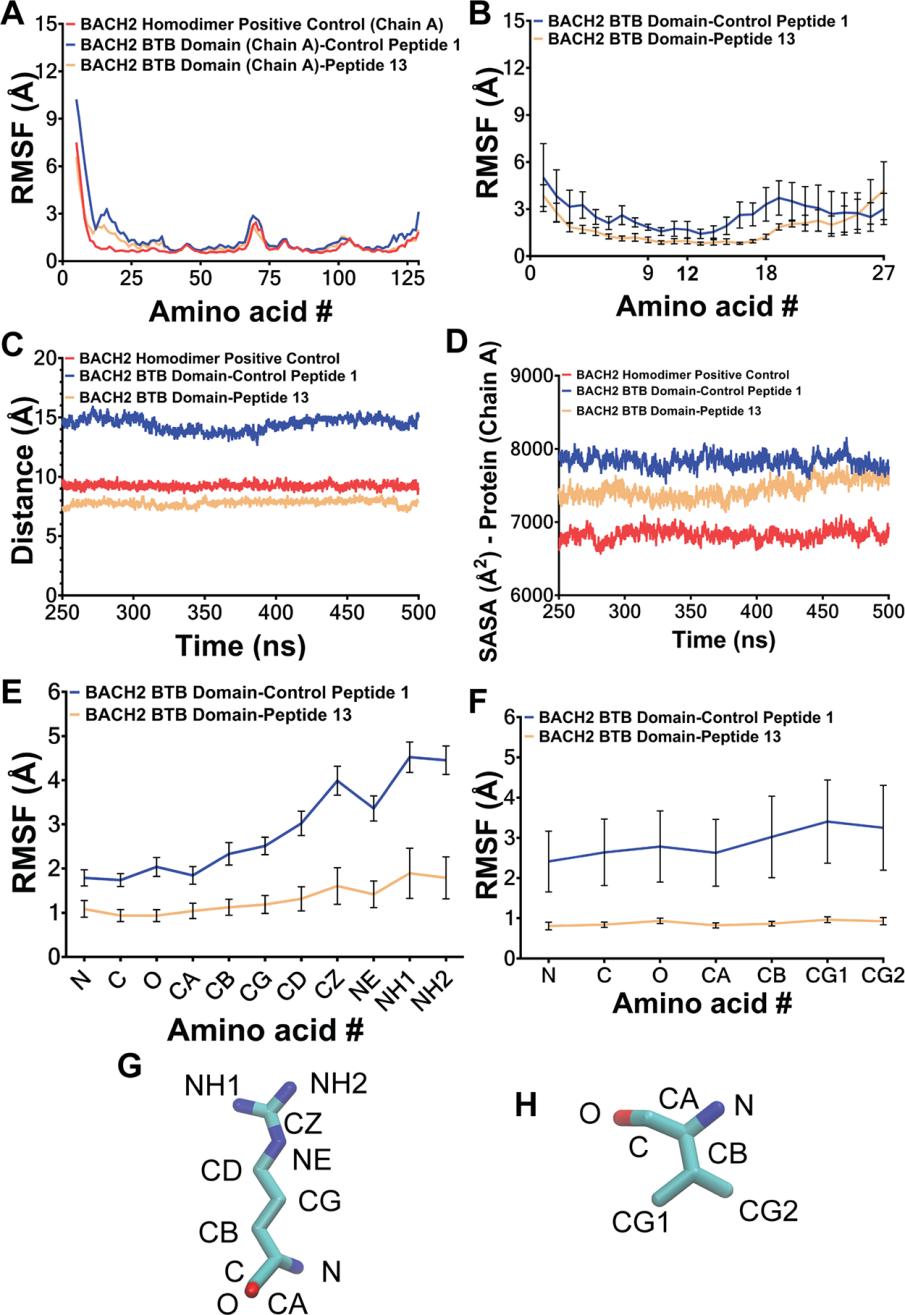
Analyses of α carbon flexibility, distance, and solvent-accessible surface area (SASA). In analyses of α carbon flexibility, distance, and solvent-accessible surface area (SASA), the BACH2 BTB domain–peptide 13 complex demonstrated stronger binding characteristics compared with the BACH2 BTB domain–control peptide 1 complex, exhibiting results more closely aligned with the PC. All analyses were conducted using the final 250 ns of the simulations, during which conformational stability was consistently observed. The presented data represent the average measurements from four simulation replicates. (A) Root-mean-square fluctuation (RMSF)-based α carbon flexibility analyses were performed for the BACH2 BTB domain monomer (chain A) in the BACH2 BTB domain homodimer and in complexes with control peptides 1 and peptide 13. Increased flexibility was observed at the N- and C-terminal regions of the BACH2 BTB domain monomers. Notably, the complex containing peptide 13 exhibited lower flexibility in these regions compared to control peptide 1 and showed values more similar to those of PC. This reduction in flexibility is interpreted as being consistent with an increase in binding affinity. (B) Similar trend was observed in the RMSF-based α carbon flexibility analysis of the peptides themselves. α Carbon atoms of control peptide 1, particularly between residues 6 to 19 (corresponding to the terminal region of the cell-penetrating segment and its immediate downstream residues), showed higher flexibility than those of peptide 13. This relative rigidity of peptide 13 is suggested to contribute to its higher affinity for the BACH2 BTB domain compared with peptide 1. (C) To further elucidate the relationship between peptide 13’s RMSF-based backbone rigidity and its binding behavior, a distance analysis was performed. For this analysis, amino acids that contribute to the MM-PBSA predicted RBFE of peptide 13 from both the peptide and target sides were examined across the PC homodimer complex and the complexes with control peptide 1 and peptide 13. In the PC, the distance between the center of mass of residues 122 and 123 on chain A and residues 11–24 on chain B (representing the region where the original peptide is predicted to bind) was used as the reference (∼9 Å). In the peptide 1 and peptide 13 complexes, distances between the center of mass of residues 122 and 123 on chain A and residues 6, 9, 10, 13, and 16 on chain B (the peptide) were measured. The results showed that peptide 13 was located even closer (∼7 Å) to the BACH2 BTB domain monomer than in the homodimer PC, while control peptide 1 remained at a distance of ∼15 Å. These results are consistent with the observed relative binding free energies. (D) To validate the findings from the distance analysis, SASA analysis was performed. As the binding interface expands, the SASA value typically decreases, offering a secondary verification step through reduced solvent exposure. In the PC homodimer, the SASA of chain A (BACH2 BTB domain), which is bound to a relatively large protein (chain B), was approximately ∼6900 Å^2^. Although peptide 13 approached a SASA value similar to that of control peptide 1 during the final 50 ns of the simulation—despite its stronger RBFE—it generally exhibited a lower SASA (∼7500 Å^2^) than control peptide 1 (∼7900 Å^2^) over the final 250 ns, aligning more closely with the PC. In parallel with the distance data, these findings support the hypothesis that peptide 13 has a greater capacity to occupy a broader surface area on the BACH2 BTB domain. (E) RMSF-based flexibility values, calculated from the final 250 ns of the simulations, were shown for all atoms of Arg9 located in the cell penetrating peptide region. Peptide 13 exhibited significantly lower flexibility than the control peptide across all atoms of Arg9, with statistical significance (*p* < 0.05). (F) RMSF-based flexibility values for all atoms of Val16 located in the designed region, calculated from the final 250 ns of the simulations, were presented. Peptide 13 demonstrated lower flexibility than the control peptide across all atoms. Although a consistent trend was observed in this second analysis, the difference did not reach statistical significance, with the *p*-value approaching but remaining above the threshold (*p* < 0.12). (G, H) Atom names and spatial positions of the Arg9­(G) and Val16­(H) residue are illustrated.

In the next step, principal component analysis (PCA) was employed to identify the dominant motions corresponding to the collective movements of atom groups throughout the simulations. For this analysis, the BACH2 BTB domain monomer from chain A of the PC homodimer, from chain A of the BACH2–Control Peptide 1 complex, and from chain A of the BACH2–Peptide 13 complex were used. This approach enabled a comparative investigation of the collective dominant motions observed in the PC BACH2 monomer versus those seen in complexes containing the low-affinity Control Peptide 1 and the high-affinity Peptide 13. As a first step, scree plots of eigenvalues vs eigenvector indices were generated to assess the flexibility of the BACH2 monomers within each complex. The final 250 ns of four replicate MD simulations were used for this analysis (Figure 7A,C,E). In all simulations, the first few eigenvectors accounted for the majority of the motions of the BACH2 BTB domain (Figure 7A,C,E). Notably, the BACH2 BTB domain monomers from both the PC and the Peptide 13-containing complex exhibited a more rigid structure compared to the monomer in the Peptide 1-containing complex, particularly along the first eigenvector (Figure 7A,C,E). Subsequently, scatter plots of the projections of the MD trajectories onto the first two principal components were examined. In line with the observed rigidity, the BACH2 BTB domain monomers from the PC and Peptide 13 complexes were found to sample a narrower conformational space relative to their counterpart in the Peptide 1-containing complex (Figure 7B,D,F). Taken together, these findings suggested that the BACH2 BTB domain monomer in the high-affinity Peptide 13 complex exhibits collective dominant motions more closely resembling those of PC, further supporting its stronger interaction profile.

**Figure 7:**
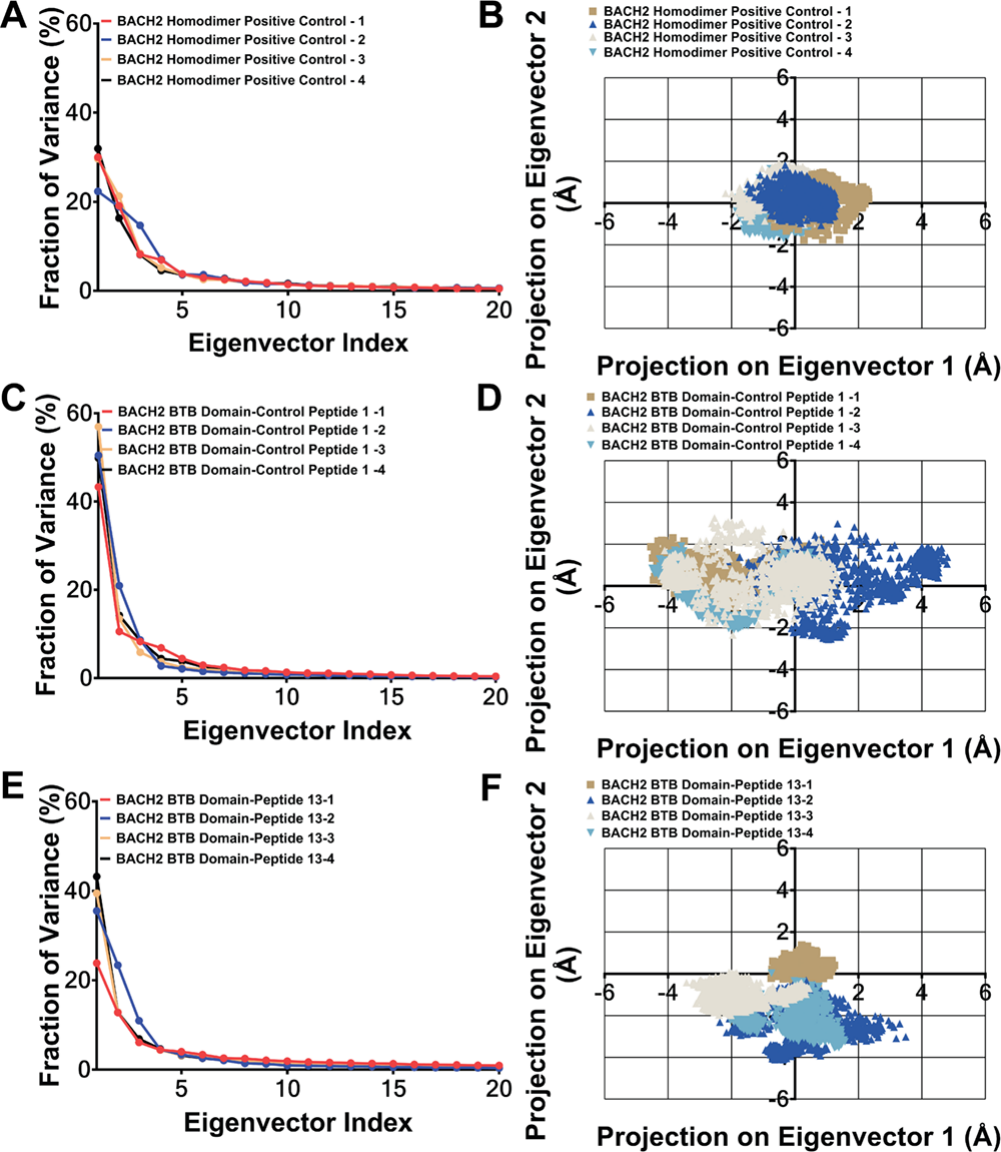
Fraction of variance vs eigenvector index plots and trajectory projections onto the principal components obtained from essential dynamics (PCA) analysis over the final 250 ns of simulations for the PC BACH2 BTB domain homodimer, the BACH2 BTB domain monomer–control peptide 1, and the BACH2 BTB domain monomer–peptide 13 complexes. To observe collective dominant motions, the BACH2 BTB domain monomer from chain A was isolated from each system and subjected to essential dynamics analysis. (A, C, E) Scree plots showing the fraction of variance as a function of eigenvector index for the last 250 ns of four replicate 500 ns MD simulations for each complex. Trend indicates that the BACH2 BTB domain monomers from the PC and the Peptide 13-containing complexes exhibit a more rigid structure compared with their counterpart in the peptide 1-containing complex, particularly with respect to the first eigenvector. (B, D, F) Scatter plots showing the projection of MD trajectories onto the first two principal components. These plots reveal that the BACH2 BTB domain monomers from the PC and peptide 13-containing complexes explore a narrower conformational space relative to the monomer in the peptide 1-containing complex. Overall, these observations suggested that the dynamic behavior of peptide 13 on the BACH2 BTB monomer was more similar to that of found in the PC complex.

To better understand the differences in conformational space sampling observed along the first and second eigenvectors, Cα atom mobility analyses were performed for the amino acids contributing to these eigenvectors. This analysis clarified which specific amino acids of the BACH2 BTB domain monomer were primarily responsible for the observed conformational space sampling. In agreement with the conformational patterns observed in the PCA projections, the N-terminal region of the BACH2 BTB domain displayed notably higher mobility in the complex containing control peptide 1 compared with both the PC and the peptide 13-containing complex, for both eigenvector 1 (PC1) (Figure 8A,C,E) and eigenvector 2 (PC2) (Figure 8B,D,F). These results implied that the homodimeric structure of the BTB domain or the binding of Peptide 13 to the BACH2 BTB monomer imparts a similar dynamic restraint on the N-terminal region, reducing its collective dominant motions and resulting in lower mobility, unlike what is observed in the control peptide 1 complex.

**Figure 8:**
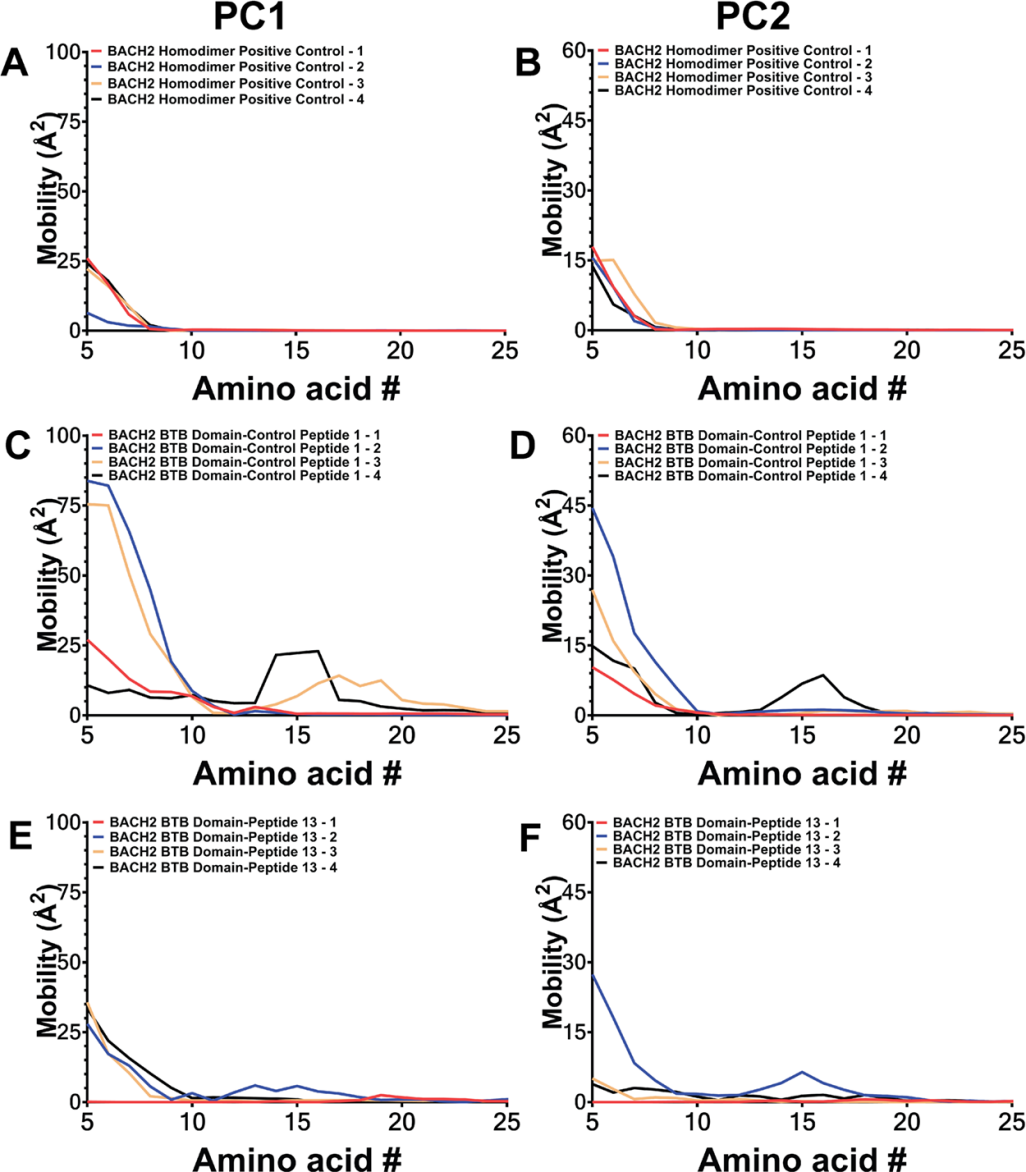
Cα atom mobility analysis of amino acids contributing to the first and second eigenvectors in the essential dynamics (PCA) analyses of the simulations for the PC BACH2 BTB domain homodimer, the BACH2 BTB domain monomer–control peptide 1 and the BACH2 BTB domain monomer–peptide 13 complexes. As the most prominent contributors to the dominant motions were found to be the first 25 amino acids located in the N-terminal region of the BACH2 BTB domain monomer, this region was analyzed in detail. (A, C, E) Mobility profiles of amino acids contributing to eigenvector 1 are shown, derived from the final 250 ns of four replicate 500 ns MD simulations for each complex. Consistent with the fraction of variance plots (scree plots) and the sampling of conformational space inferred from the trajectory projections onto the principal components (scatter plots) in Figure 7, the mobility of N-terminal residues was generally attenuated in both the PC and the Peptide 13 complex, whereas an increase in N-terminal flexibility was observed in the control peptide 1 containing complex. (B, D, F) Mobility profiles of amino acids contributing to eigenvector 2, obtained from the same simulation windows. These results were also parallel to the mobility trends observed for eigenvector 1. Collectively, the findings suggested that binding of the high affinity peptide, as seen in peptide 13, dampens N-terminal dynamics, a pattern that was also observable in the PC complex.

To further interpret the N-terminal mobility patterns observed in the analysis through visualization, the first principal component of the third replicate from each simulation was examined using shortened trajectory projections (Supplementary Figure 6). At this stage, only the first principal component was analyzed as the eigenvectors generally exhibited similar motion patterns. The third replicate was selected because it provided a structurally representative model for visualizing mobility effects on the structure. As shown in Supplementary Figure 6A, in the PC complex, the motion of the BACH2 BTB monomer—interacting with the second monomer—was limited to the terminal amino acids (residues 1–25) of the N-terminus. In Supplementary Figure 6C, the BACH2 BTB domain monomer from the Peptide 13-containing complex also exhibited N-terminal motion, similar to the PC. However, since peptide 13 is a smaller molecule compared with the BTB domain monomer in the homodimer, the region following residue 25 extended toward the BACH2 BTB domain core and established interactions that resulted in structural stabilization. This indicated that peptide 13 binding contributes to N-terminal stabilization via an alternative interaction network. This outcome was considered to be consistent with the higher binding affinity of peptide 13, which even surpasses that of the PC. Furthermore, the region spanning residues 97–103, which is spatially opposite the predicted peptide-binding interface, and the 120–129 region, known to contribute to peptide 13 affinity, remained stable in both the PC and the peptide 13-containing complex. In contrast, the behavior of the BACH2 BTB monomer in the control peptide 1 containing complex deviated from these patterns. As shown in Supplementary Figure 6B, this monomer exhibited greater mobility involving a larger portion of the N-terminal residues. Although the N-terminus approached the monomer’s core structure, its motion was less suppressed compared to that seen in the Peptide 13 complex. Additionally, the regions spanning residues 97–103 and 120–129 underwent more conformational alterations relative to each other. In conclusion, the structural insights obtained from these shortened trajectory projections were consistent with the flexibility, conformational space sampling, and mobility data derived from the principal component analysis.

Finally, to better understand the internal motions of the N-terminal region and adjacent residues within the BACH2 BTB domain monomer, a cross-correlation analysis of Cα atom fluctuations was performed (Supplementary Figure 7). The results revealed that in both the PC BACH2 BTB domain monomer and the monomer from the Peptide 13-containing complex, the regions immediately following the N-terminal residues—designated as Regions 1, 2, and 3—generally exhibited positive correlations (Supplementary Figure 8A,C). In contrast, for the control peptide 1-containing complex, these marked regions predominantly showed a shift toward negative correlation (Supplementary Figure 7B). These findings suggested that control peptide 1 binding fails to synchronize the N-terminal motions with the protein core, whereas Peptide 13 binding, similar to PC, facilitated a coordinated dynamic behavior between the N-terminus and the protein core. In conclusion, the cross-correlation analysis supported the findings from the interpolation of eigenvector 1 onto the structure, confirming the observed motion patterns.

### 3.5 Determination and Ranking of the *In Vitro* Binding Affinities of Selected BACH2 BTB Domain-Targeting Peptides Using Supernatant Depletion Assay

After detailed MD analyses of the complexes, next, SDA was used to assess *in vitro* binding affinity ranking of computationally selected peptides, specifically, the highest-affinity peptide 13 and the low-affinity control peptide 1 so that a wet lab based verification step could be established for relative binding free energies estimated by the computational MM-PBSA method. More specifically, through utilizing SDA, nonequilibrium *K*_d_ of the selected peptides toward full-length BACH1 and BACH2 proteins were retrieved. Following binding analyses via SDA, in the subsequent step, functional validation of the peptides was performed using coimmunoprecipitation experiments.

In order to execute SDA and coimmunoprecipitation experiments, first, the peptides were synthesized by a commercial provider with selected parameters. Following peptide synthesis, myc-DDK-tagged full-length BACH2 was overexpressed in HEK293T cells, a model system previously used for *in vitro* BACH2 dimerization studies.^22^ Protein extracts were prepared from these cells to generate BACH2-containing supernatants. As a negative control, myc-DDK-tagged full-length BACH1, the protein selected during computational analysis, was also overexpressed in the same cell line.

After peptide synthesis, SDA was conducted to validate computationally predicted relative binding free energies *in vitro* through determination of the affinity ranking of the peptides.

A critical step in accurately estimating the *K*_*d*_ is selecting a biochemical assay method that maintains the system at equilibrium.^68^ For protein–peptide interactions, one strategy to achieve this is to ensure that the sedimentation constant of the protein–peptide complex is lower than that of either reactant alone, thereby allowing the complex to be selectively precipitated without disturbing equilibrium. For example, a peptide immobilized on beads can be incubated with a low-concentration protein extract containing the protein of interest. This incubation could allow the system to reach a binding equilibrium between the peptide and target protein. Upon precipitation of the bead-bound peptide, any protein bound to it will also be coprecipitated without significantly disrupting the equilibrium. Consequently, a portion of the unbound protein remains in the supernatant. Rather than analyzing the precipitate, quantifying the remaining free protein in the supernatant provides a means of estimating binding ratios without disrupting the equilibrium state. Plotting the percentage of free (unbound) protein remaining in the supernatant as a function of peptide concentration enables estimation of the dissociation constant (*K*_d_). This supernatant depletion methodology can be applied using either purified protein solutions or crude protein extracts.^68^ Since the *K*_d_ estimated with this methodology represented the first of such measurements in the literature for our peptides and targets, it was assumed that they reflect nonequilibrium *K*_d_ estimates, and emphasis was placed on the relative ranking between peptides. This ranking was then compared to the computational MM-PBSA-based predictions, providing an *in vitro* validation layer for the peptide selection strategy.

As shown in Supplementary Figure 8, full-length myc-DDK-tagged BACH1 and BACH2 proteins were successfully overexpressed in HEK293T protein extracts following transfection. An optimization experiment was also performed to determine the minimum amount of protein required for immunoblotting for the detection step of SDA (Supplementary Figure 9). Based on this trial, the minimum detectable antimyc signal for myc-DDK-tagged BACH2, enabling analysis of the peptide’s binding effect from the bands, was determined to be approximately 2.5 μg (Supplementary Figure 9). Accordingly, for the samples to be loaded onto the gel after SDA, the total protein amount was adjusted to a volume corresponding to 2.27 μg.

Following this overexpression verification and protein amount optimization for SDA analyses, the main experiment consisting of SDA was executed. According to this analysis, *K*_d_ for the interaction of peptide 13 with myc-DDK tagged full length BACH2 was determined to be 148.4 nM (95% CI: 35.2–463.1) (Figure 9A,C). For the interaction of control peptide 1 with Myc-DDK tagged full length BACH2, *K*_d_ was determined to be 493.3 nM (95% CI: 163.8–1876) (Figure 9B,C). Although both *K*_d_ values showed overlapping 95% confidence intervals (CI) and were not statistically different, an approximately 3-fold higher affinity was observed in favor of peptide 13 between the estimated *K*_d_ values. To verify the BACH2 specificity of peptide 13, SDA was also repeated with myc-DDK tagged full length BACH1, where the affinity was found to be 187.8 nM (95% CI: 12.43–1473 nM) (Supplementary Figure 10A,B). This data also indicated that peptide 13 might be more specific for BACH2 than BACH1, although not at a statistically significant level. Despite the fact that statistical differences do not exist with our experimental framework, which necessitates further replicated measurements, these findings were evaluated as carrying a trend toward validating the data obtained from the computational study.

**Figure 9:**
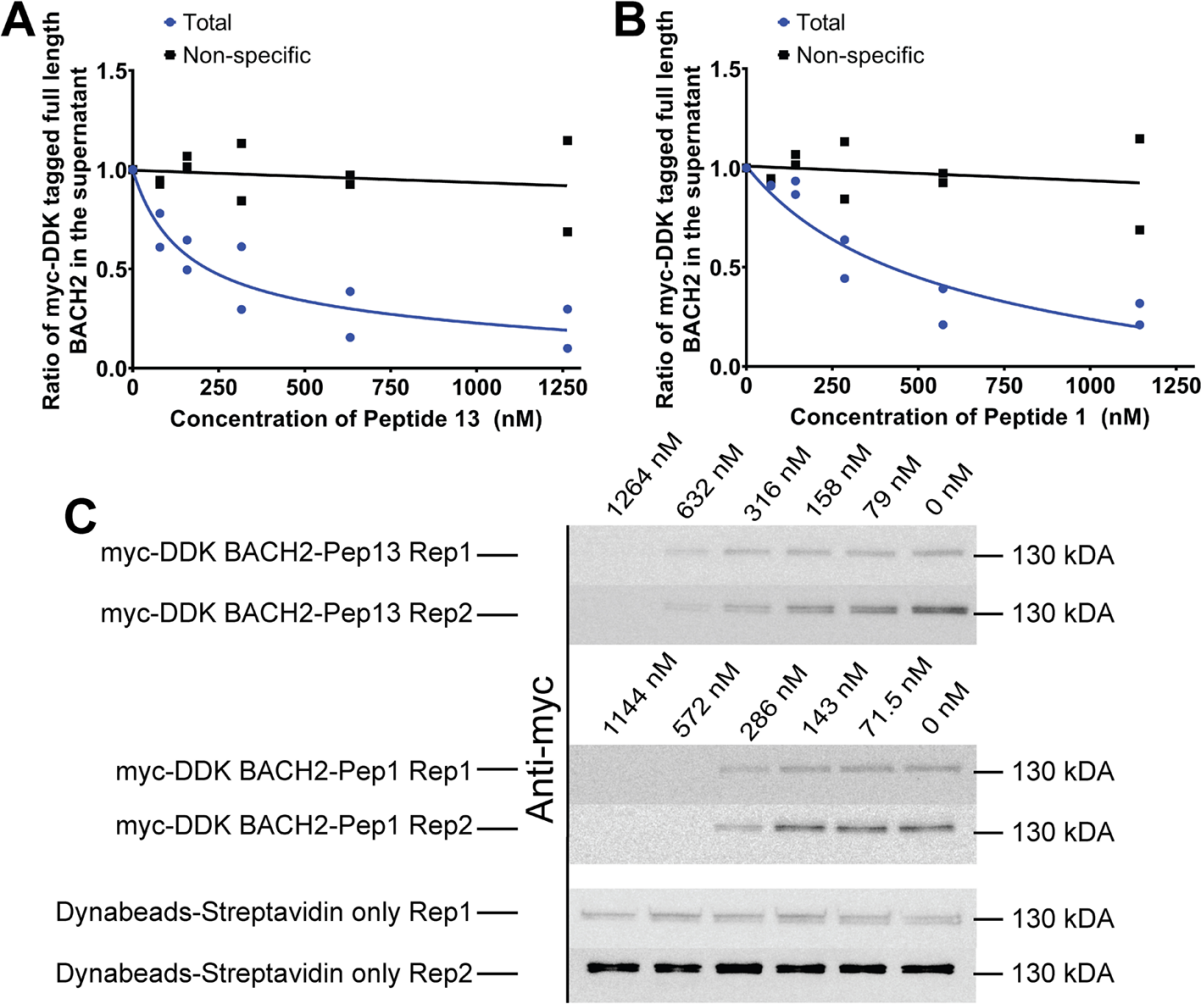
Determination of *K*_d_ for peptide 13 and control peptide 1 against myc-DDK-tagged BACH2 via SDA. (A) Based on this analysis, peptide 13 exhibited a *K*_d_ of 148.4 nM (95% CI: 35.2–463.1 nM) for myc-DDK-tagged BACH2. (B) The control peptide 1 displayed an *K*_d_ of 493.3 nM (95% CI: 163.8–1876 nM) for myc-DDK-tagged BACH2. Although the 95% confidence intervals overlapped, preventing statistical significance, the estimated *K*_d_ values indicate ∼3.3-fold higher affinity for peptide 13 compared to control peptide 1, consistent with the computational predictions. (C) Representative immunoblotting data corresponding to the analyses in panels A and B, which were quantified by densitometry (CI, confidence interval; Rep, replicate).

### 3.6 Co-Immunoprecipitation Based Assessment of Functional Activity for Computationally Selected Peptides toward Inhibiting Interaction between myc-DDK Tagged Full Length BACH2 and turboGFP Tagged Full Length BACH2

The objective at this point was to investigate the suppressive efficacy of selected peptides on BACH2 homodimerization in order to demonstrate their functionalities. Toward this objective, full-length BACH2 tagged with myc-DDK and full-length BACH2 tagged with tGFP (turboGFP) were overexpressed at the protein level in HEK293T cells (Figure 10A). Following this overexpression, coimmunoprecipitation studies were performed (Figure 10B). As shown in Figure 10B, the anti-tGFP antibody-conjugated Dynabeads–Protein G complex yielded both tGFP and myc signals, demonstrating the interaction between BACH2 proteins bearing these two tags. The specificity of the interaction was confirmed using appropriate controls, showing that the interaction did not originate from the Dynabeads–Protein G conjugate or the antibody Fc region (Figure 10B). At this point, reverse coimmunoprecipitation (IP: antimyc, IB: anti-tGFP) was attempted; however, due to observed non specific binding of the antimyc-antibody, this approach was omitted. Following demonstration of the interaction, it was shown that 0.5 μM peptide 13 was insufficient to disrupt this interaction, whereas 2.5 μM peptide 13 and control peptide 1 completely abolished the myc-DDK-BACH2/tGFP-BACH2 interaction (Figure 10C). Subsequently, to test whether peptide 13 is a more potent BACH2 binder than control peptide 1, as suggested by *in silico* data and *in vitro* supernatant depletion analysis, concentrations of peptides ranging from 0.5 to 2.5 μM were tested for their effects on the interaction (Figure 10D). For this purpose, the impact of 1 and 1.5 μM concentrations of peptide 13 and control peptide 1 on the interaction were analyzed, revealing that peptide 13 acts as a potentially more powerful coimmunoprecipitation disruptor at these concentrations (Figure 10D). Additionally, densitometric analysis was performed for the coimmunoprecipitation disruption shown in Figure 10D, demonstrating that 1 μM peptide 13 exhibited a decrease approaching the threshold of marginal statistical significance (Supplementary Figure 11).

**Figure 10:**
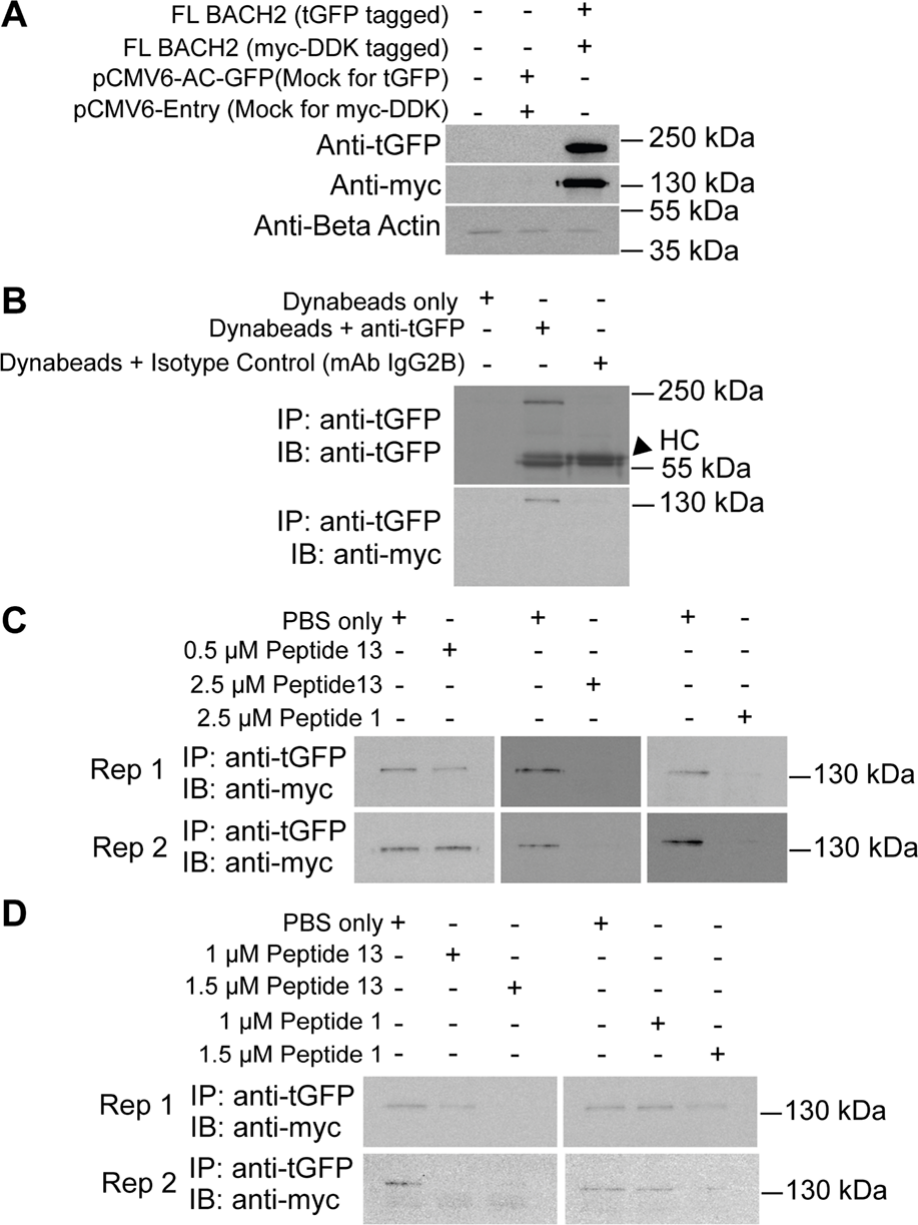
Interference of the selected peptides with the interaction of tGFP tagged BACH2 and myc-DDK tagged BACH2, analyzed through coimmunoprecipitation. (A) Full-length (FL) tGFP (turbo GFP) tagged BACH2 and full-length myc-DDK tagged BACH2 were contransfected and overexpressed in HEK293T cells (lane 3). As controls, backbone plasmids pCMV6-AC-GFP (backbone plasmid for tGFP) and pCMV6-Entry (backbone plasmid for MYC-DDK) were similarly subjected to overexpression, and it was confirmed that no proteins of the same molecular weight were observed (lane 2). Additionally, it was confirmed for control purposes that no proteins corresponding to these tags existed in normal HEK293T cells (lane 1). (B) In protein extracts that simultaneously overexpress FL-tGFP BACH2 and FL-myc-DDK tagged BACH2, a control coimmunoprecipitation experiment was implemented to present homodimerization. Immunoprecipitation band was revealed to be antigen-specific through lack of corresponding bands in Dynabeads only and in Dynabeads only isotype control lanes (Above and below immunoblots). Moreover, 55 kDa band was marked to depict the antibody heavy chain that has a molecular weight around 50 kDa (above immunoblot). Overall, signals obtained between 250 and 130 kDa bands indicated that tGFP-tagged FL BACH2 could coimmunoprecipitate with myc-DDK tagged FL BACH2, thus indicating an expected interaction (below immunoblot). HC: heavy chain (antibody heavy chain). (C) After establishing the coimmunoprecipitation system, the peptide interaction phase was initiated. In the first stage, peptide 13 was incubated with the same amount of total protein extract used in coimmunoprecipitation at 0.5 μM level (corresponding to ∼3 times *K*_d_) (left) and at 2.5 μM level (∼16 times *K*_d_) (middle). As the control group, the same volume of PBS was added instead of peptide (PBS only). After peptide incubation, same coimmunoprecipitation procedure shown was applied. As a result of this analyses, it was observed that 0.5 μM peptide 13 was not very effective in disrupting coimmunoprecipitation (left), but at 2.5 μM level, this interaction was completely eliminated (middle). To understand whether the effect of 2.5 μM peptide 13 was comparable with that of control peptide 1, the same experiment was repeated with 2.5 μM peptide 1 (right), and it was observed that this peptide also completely prevented the interaction at this concentration. Rep1 and Rep2 represented experimental duplicates. (D) Since 2.5 μM peptide 13 and control peptide 1 were completely successful in eliminating the coimmunoprecipitation indicating probable interference with BACH2 dimerization, the coimmunoprecipitation experiment was repeated with lower concentrations for peptide 13 and control peptide 1. Thus, the possible difference between peptide 13 and peptide 1 was attempted to be revealed. At this stage, the effect of 1 and 1.5 μM peptide 13 and peptide 1 on tGFP-FL-BACH2/myc-DDK-FL-BACH2 coimmunoprecipitation was investigated. The utilization of 1 and 1.5 μM peptide 13 showed a more effective interference with immunoprecipitation compared with the control peptide 1 at the corresponding concentrations. This information was evaluated as confirming the higher BACH2 affinity of peptide 13 compared with control peptide 1. Rep1 and Rep2 represented experimental duplicates.

## 4 Discussion

As a fundamental contribution, to our knowledge for the first time in the literature, here we report selected peptide sequences capable of interfering with BACH2 dimerization revealed through the combination of computational and *in vitro* methods. Thus, a promising option has been obtained for future attempts toward inhibiting of BACH2 at different settings. More specifically, in order achieve these aims, first we identified a precursor peptide sequence from available homodimeric BACH2 BTB domain structure through a initial pilot study (Figure 2, Table 1, and Supplementary Figure 1). In this initial preliminary study, 100 ns MD simulations of the peptide with target BACH2 BTB domain were executed and based on these simulations, MM-PBSA-based RBFE estimation was completed (Table 2 and Supplementary Figure 2). Upon retrieving positive findings with the preliminary peptide sequence, sequence optimization and establishment of a potential peptide library was completed within the scope of initial pilot study (Tables 3 and 4). This peptide library was then subjected to peptide structure prediction and peptides were selected using a global docking algorithm based on their binding preference toward the native binding site on one of the monomers of the BACH2 BTB domain monomer (Figure 3, Table 5, Supplementary Table 1, and Figure 3). Using global docking data from the selected targets protein–peptide complexes, these complexes were redocked using a local docking program, which were fed into 500 ns MD simulations. These longer MD simulations were first analyzed for conformational stability, then commonly stable frames were used again for MM-PBSA based RBFE estimation (Figure 4, Table 6, Supplementary Figures 4, and 5). After MM-PBSA based elimination of the other peptides, high affinity peptide 13 and the control peptide 1 were selected and subjected to in depth MD analyses together with the PC complex, BACH2 BTB domain homodimer. Moreover, since MM-PBSA also indicated hydrophilic dominance in peptide 13 binding, the approach to in depth analyses was initiated starting from that point onward. At this point, hydrogen bond analyses of amino acids contributing to the MM-PBSA predicted binding free energies were completed (Table 7 and Figure 5A). Following hydrogen bonding, critical salt bridge analyses were also performed (Figure 5). In addition to these electrostatic analyses, hydrophobic interaction analyses were also implemented for the selected complexes (Supplementary Table 2). After hydrophobic interaction, RMSF, distance, and SASA analyses were also completed to comprehend the behavior of the peptides in detail (Figure 6). In addition, principal component and cross-correlation analyses were executed on the target BACH2 BTB domain monomer in all experimental groups to decipher conformational preferences of spatial organization (Figures 7, 8, and Supplementary Figures 6, and 7). All together, in depth MD analyses clearly indicated that MM-PBSA based selection of peptide 13 could show strong signs of binding, which persisted into at least the final 250 ns of the MD simulations. After obtaining promising results for the peptide 13 in the computational part, we extended the study into *in vitro* part, where peptide 13 as well as control peptide 1 was challenged for their binding affinity toward full length BACH2 protein and their functional capacity to reduce dimerization of BACH2 was tested. For determination of *in vitro* *K*_d_ values of selected peptides, overexpression of BACH2, followed by SDA was conducted (Figure 9 and Supplementary Figures 8–10). At the final phase, capability of selected peptides to disrupt BACH2 homodimerization was deciphered through coimmunoprecipitation (Figure 10). Overall, it has been demonstrated that peptide 13, which showed high affinity for BACH2 through *in silico* analyses and *in vitro* SDA, can also disrupt BACH2 homodimerization more efficiently than control peptide 1. Hence, a candidate peptide molecule that could serve as a BACH2 homodimer formation inhibitor has been discovered.

The inhibition of transcription factors by peptides has been presented as a concept previously addressed in the literature. A peptide-based c-Jun antagonist has been shown to disrupt DNA binding capability by binding to c-Jun.^73^ Similarly, the development of a peptide for preventing dimerization of BZLF1 transcription factor has been recently reported.^74^ As an another example, a cyclic peptide reported against dimerization of C-terminal binding protein (CtBP) transcriptional repressor protein could also be given.^75^ These peptide design examples could also be provided for other transcription factors as well, such as STAT3 ^76^ and FOXP3.^77^ Additionally, through utilizing computational methods, our group has also predicted a C terminal binding peptide capable of targeting dimerization of NFAT5 DNA binding domain and determined its relative binding free energy using MD simulations.^78^ All these background studies clearly demonstrated that transcription factors can be targeted via peptides, and through our study, a novel inhibitory peptide targeting dimerization of the BACH2 BTB domain has been characterized using both *in silico* and *in vitro* approaches.

Previously accumulated data clearly indicated that the computationally determined experimental absolute binding free energy for the BACH2 BTB domain homodimer is not available. Furthermore, no attempt has been taken toward defining its *in vitro* *K*_d_. Thus, the computationally predicted RBFE values and experimentally estimated *K*_d_ values for the protein–protein and protein–peptide complexes were considered to be relative values. In line, comparative assessment with controls became the main determinant for interpreting binding free energy and *K*_d_ levels. Within our study, the entropic term estimated by the MM-PBSA methodology used for RBFE prediction also shifted the binding free energy values numerically to positive values. Parallel to this observation, it has been previously reported that the quasi-harmonic approximation method used for estimation of entropic term was the underlying reason.^65^ This observation constitutes an aspect of our study that is open to improvement, and the completion of more advanced MD methodologies, such as free energy perturbation or steered MD simulations, is recommended for future estimation attempts to compute absolute binding energy values. Furthermore, to confirm the predicted *in vitro* *K*_d_ value, the application of methodologies such as surface plasmon resonance, biolayer interferometry or isothermal titration calorimetry is required.

Within the MMPBSA study, the internal dielectric constant was taken as 1 for the estimation of the polar solvation energy. In many previously reported studies, different internal dielectric constant values have been used, and particularly, it has been shown that the compatibility of experimental data with computational studies increases with increasing internal dielectric constant.^79–81^ Therefore, in future MM-PBSA studies, optimization of internal dielectric constant was recommended to be utilized, or it should be demonstrated that the RBFE comparisons obtained from the studies show patterns independent of the internal dielectric constant applied during computations.

Another point that should be highlighted within our study is the impact of the cell-penetrating peptide on the binding event. The MM-PBSA study revealed that some amino acids of the cell-penetrating peptide contributed to the binding of peptide 13, which displayed high affinity and functionality both *in silico* and *in vitro* (Figure 4). Although there were amino acid contributions from the designed region of peptide 13 as well (Figure 4), the substantial contribution of the HIV TAT 47–57 cell-penetrating peptide constituted an area of our study that is clearly open to deeper investigation. While the FoldX based optimization study completed at the very beginning succeeded in engaging two amino acids from the designed region of peptide 13 to get involved in the target interaction, whether the use of different cell-penetrating peptides would yield the same result and whether this condition was specific only to HIV TAT 47–57 are concepts that need to be addressed in the future. Considering that cell-penetrating peptides may have off-target effects,^82^ peptide design strategies need to be evaluated accordingly. Furthermore, despite the fact that the FoldX method was beneficial for allocating new hydrophilic amino into the interaction of protein and peptide, current literature contains more effective strategies for peptide design. While FoldX-based mutagenesis provides a rapid framework for estimating binding free energy changes, its reliance on static structural templates may limit the exploration of novel binding geometries. De novo design methods such as RFdiffusion/ProteinMPNN^83,84^ and BindCraft^85^ enable backbone-level sampling unconstrained by existing scaffolds followed by full-sequence side-chain design, collectively supporting the generation of binders with optimized shape complementarity. Thus, through future studies, utilization of these modern AI based methods will likely bring unexplored peptide sequence and structure possibilities together with high-throughput screening capability toward larger peptide libraries for targeting BTB domain of BACH2.

The only direct physical data regarding homodimerization of BACH2 BTB domain is the crystal structure presented in PDB ID: 3OHU. No other earlier studies could be found that has demonstrated this homodimerization event through coimmunoprecipitation experiments. In terms of closing this gap, our manuscript has generated a novel output through which a co-IP experiment presents full length BACH2 homodimerization (10). The ability to disrupt this coimmunoprecipitation event through peptide treatment, particularly applicable to high-affinity peptide 13, has emerged as a factor that advances the currently available knowledge in this field. Despite the claimed interaction data, whether the peptides are direct and specific binders of BACH2 BTB domain remains as a question yet to be answered. Moreover, since all studies were completed with total protein extracts, it remains unclear whether peptide interaction was mediated through a different peptide-macromolecule relationship and/or an allosteric effect both in supernatant depletion analyses and in coimmunoprecipitation experiments. In future, interaction of peptides should be investigated with purified proteins so that one could establish whether peptide binding is direct for BACH2 or indirect though other means.

Although cell-penetrating peptide addition has been completed for potential cellular utilization, how peptide 13 will affect BACH2 homo- or heterodimerization in living cells and how it will behave toward healthy or cancer cell lines remain questions worth investigating. Since BACH2 is known to be a suppressor of the NRF2 transcription factor, how peptide 13 modifies NRF2 target genes at the mRNA and protein levels should be thoroughly investigated. Additionally, due to BACH2’s role in immunosuppression, analysis of peptide 13 in *in vitro* and in vivo immunosuppression models is strongly recommended. From a genetic perspective, it is also known that some BACH2 mutations are causally related to various pathologies. However, the conformational changes that most of these variants induce on the protein structure are still unknown. In the future, when peptide 13 is tested on BACH2 in terms of binding using computational methodologies, incorporating these mutations in target BACH2 molecules will guide to a deeper understanding of peptide 13 behavior in different genetic backgrounds.

Recent back-to-back studies published in Nature Immunology have collectively established BACH2 as a central and dose-dependent regulator of T cell stemness, exhaustion, and antitumor efficacy, underscoring the broad therapeutic relevance of modulating BACH2 activity in cancer immunotherapy.^86–89^ Hu et al. demonstrated that BACH2 dose-dependently governs the hierarchy of long-term stem-like CAR T cell differentiation, with the highest BACH2 expression marking cells of superior antitumor capacity both before infusion and after tumor clearance. Conti et al. further showed that while constitutively high BACH2 expression enforces quiescence and impairs tumor control, calibrated low-dose BACH2 expression promotes CD8^+^ T cell persistence without compromising effector function by partially restraining terminal differentiation through attenuation of AP-1-dependent gene programs. Chang et al. complemented these findings by linking BACH2 to a degradation domain to achieve tunable expression, successfully preventing exhaustion while preserving potent effector function in CAR T cells targeting both liquid and solid tumors and further identifying a correlation between BACH2 activity and favorable clinical outcomes in leukemia patients. In regards to these recent evidence, our BTB domain-targeting peptide inhibitor peptide 13, by potentially disrupting BACH2 homodimerization, offers a probable contribution to the physiological tuning of BACH2 activity to achieve precise modulation.^86–89^

## 5 Conclusions

In conclusion, following complementary computational and *in vitro* studies, a peptide-based molecule with the potential to inhibit BTB domain homodimerization of BACH2, the nuclear suppressor of NRF2 transcription factor, has been identified as peptide 13. A potentially modulatory factor has been defined both for a possible activation of the NRF2 transcription factor, which is a regulator of cellular antioxidant capacity, and also for the elimination of immunosuppression in cancer. In future, determining the absolute RBFE of the identified peptide 13 to BACH2, validating our *K*_d_ estimations, defining the effects of various cell-penetrating peptide combinations on peptide 13 within the presented framework, and confirming the cellular and organism-level effects of the peptide through *in vitro* cell culture and in vivo experiments were considered to be required.

## Supplementary Material



## Data Availability

The data underlying this study are openly available in Zenodo at 10.5281/zenodo.17415474.

## Notes

Supplementary Figure 1: the possible hydrogen bonds and hydrophobic contacts between the BACH2 BTB domain monomer sequence in chain A, predicted to mediate protein–peptide interaction, and the peptide sequence predicted on chain B were analyzed using the Ligplot+; Supplementary Figure 2: RMSD analyses for the initial MD simulations implemented for peptide design; Supplementary Figure 3: possible amino acid networks governing dimerization for the potential negative controls BACH1 and BCL6 BTB domain dimers, identified using the PLIP program; Supplementary Table 1: weak interaction analyses of ClusPro based global docking generated structures of the peptide 13 in complex with the dimerization regions of BACH1 BTB domain and BCL-6 BTB domain monomers; Supplementary Figure 4: time-dependent RMSD analysis for four 500 ns MD simulation replicates of the positive control BACH2 BTB domain homodimer; Supplementary Figure 5: time-dependent RMSD analysis for four 500 ns MD simulation replicates of the following complexes: BACH2 BTB domain–peptide 13 (A), BACH1 BTB domain–peptide 13 (B), and BCL6 BTB domain–peptide 13 (C); Supplementary Table 2: carbon–carbon contacts with the highest occupancy rates observed in the last 250 ns of simulations for the BACH2 BTB domain homodimer PC complex, BACH2 BTB domain–control peptide 1 (CP1), and BACH2 BTB domain–peptide 13 complex (P13); Supplementary Figure 6: shortened trajectories along the first principal component from the essential dynamics (PCA) analysis of the third replicate simulations for the positive control BACH2 BTB domain homodimer, the BACH2 BTB domain monomer–control peptide 1 complex, and the BACH2 BTB domain monomer–peptide 13 complexes; Supplementary Figure 7: cross-correlation analysis of BACH BTB monomer retrieved from the third replicate simulations for the positive control BACH2 BTB domain homodimer, the BACH2 BTB domain monomer–control peptide 1 and the BACH2 BTB domain monomer–peptide 13 complexes; Supplementary Figure 8: verification of myc-DDK-tagged BACH1 and BACH2 overexpression in HEK293T cells via immunoblotting; Supplementary Figure 9: optimization experiment to determine the minimum protein amount required for detectable gel bands following immunoblotting of overexpressed myc-DDK-tagged BACH2; Supplementary Figure 10: determination of the dissociation constant of peptide 13 against Myc-DDK tagged BACH1 by supernatant depletion analysis; and Supplementary Figure 11: densitometric analysis of coimmunoprecipitation assay (PDF)

## Abbreviations

BACH2: transcription regulator protein BACH2 (BTB and CNC homologue 2)
*K*_d_: dissociation constant
MD: molecular dynamics
MM-PBSA: Molecular Mechanics-Poisson–Boltzmann Surface Area continuum solvation
NRF2: nuclear factor erythroid 2-related factor 2
PC: positive control
PDB: Protein Data Bank
RBFE: relative binding free energy
RMSD: root mean square displacement
RMSF: root mean square fluctuation
SASA: solvent accessible surface area
SDA: supernatant depletion assay

## References

[1] Robledinos-Antón N., Fernández-Ginés R., Manda G., Cuadrado A. (2019). Activators
and Inhibitors of NRF2: A Review of Their Potential for Clinical Development. Oxid. Med. Cell. Longevity.

[2] Ma Q. (2013). Role of Nrf2
in Oxidative Stress and Toxicity. Annual Review
of Pharmacology and Toxicology.

[3] Hayes J. D., Dinkova-Kostova A. T. (2014). The Nrf2
regulatory network provides an interface between
redox and intermediary metabolism. Trends Biochem.
Sci..

[4] Pajares M., Jiménez-Moreno N., Dias I. H., Debelec B., Vucetic M., Fladmark K. E., Basaga H., Ribaric S., Milisav I., Cuadrado A. (2015). Redox control of protein degradation. Redox Biology.

[5] Pajares M., Jiménez-Moreno N., Ángel J., García-Yagüe, Escoll M., de Ceballos M. L., Leuven F. V., Rábano A., Yamamoto M., Rojo A. I., Cuadrado A. (2016). Transcription factor
NFE2L2/NRF2 is a regulator of macroautophagy genes. Autophagy.

[6] Pajares M., Cuadrado A., Rojo A. I. (2017). Modulation
of proteostasis by transcription
factor NRF2 and impact in neurodegenerative diseases. Redox Biology.

[7] Vega M., Dodson M., Gross C., Mansour H., Chapman E., Wang T., Black S., Garcia J., Zhang D. (2016). Role of Nrf2
and Autophagy in Acute Lung Injury. Curr. Pharmacol.
Rep..

[8] Cuadrado A. (2018). Transcription Factor
NRF2 as a Therapeutic Target for Chronic Diseases:
A Systems Medicine Approach. Pharmacol. Rev..

[9] Cuadrado A., Rojo A., Wells G., Hayes J., Cousin S., Rumsey W., Attucks O., Franklin S., Levonen A.-L., Kensler T., Dinkova-Kostova A. (2019). Therapeutic
targeting of the NRF2
and KEAP1 partnership in chronic diseases. Nat.
Rev. Drug Discovery.

[10] Oyake T., Itoh K., Motohashi H., Hayashi N., Hoshino H., Nishizawa M., Yamamoto M., Igarashi K. (1996). Bach Proteins Belong
to a Novel Family of BTB-Basic Leucine Zipper Transcription Factors
That Interact with MafK and Regulate Transcription through the NF-E2
Site. Mol. Cell. Biol..

[11] Zhou Y., Wu H., Zhao M., Chang C., Lu Q. (2016). The Bach Family of
Transcription Factors: A Comprehensive Review. Clin. Rev. Allergy Immunol..

[12] Dhakshinamoorthy S., Jain A. K., Bloom D. A., Jaiswal A. K. (2005). Bach1 Competes with
Nrf2 Leading to Negative Regulation of the Antioxidant Response Element
(ARE)-mediated NAD­(P)­H:Quinone Oxidoreductase 1 Gene Expression and
Induction in Response to Antioxidants∗. J. Biol. Chem..

[13] Chen Z., Pittman E. F., Romaguera J., Fayad L., Wang M., Neelapu S. S., Mclaughlin P., Kwak L., McCarty N. (2013). Nuclear Translocation
of B-Cell-Specific Transcription Factor, BACH2, Modulates ROS Mediated
Cytotoxic Responses in Mantle Cell Lymphoma. PLoS One.

[14] Unno M., Muto A., Takeuchi A., Kometani K., Kurosaki T., Igarashi K., Saito T. (2013). Bach2 maintains
T cells in a naive
state by suppressing effector memory-related genes. Proc. Natl. Acad. Sci. U. S. A..

[15] Uittenboogaard L., Payan-Gomez C., Pothof J., van IJcken W., Mastroberardino P., van der Pluijm I., Hoeijmakers J., Tresini M. (2013). BACH2: A marker of DNA damage and ageing. DNA Repair.

[16] Tonelli C., Chio I. I. C., Tuveson D. A. (2018). Transcriptional
Regulation by Nrf2. Antioxidants & Redox
Signaling.

[17] Waxman, E. A. Bach2 is a potent repressor of Nrf2-mediated antioxidant enzyme expression in dopaminergic neurons. 2019, bioRxiv.

[18] Igarashi K., Hoshino H., Muto A., Suwabe N., Nishikawa S., Nakauchi H., Yamamoto M. (1998). Multivalent
DNA Binding Complex Generated
by Small Maf and Bach1 as a Possible Biochemical Basis for β-Globin
Locus Control Region Complex. J. Biol. Chem..

[19] Hoshino H., Igarashi K. (2002). Expression of the Oxidative
Stress-Regulated Transcription
Factor Bach2 in Differentiating Neuronal Cells. Journal of biochemistry.

[20] Albagli O., Dhordain P., Deweindt C., Lecocq G., Leprince D. (1995). The BTB/POZ
domain: A new protein-protein interaction motif common to DNA- and
actin-binding proteins. Cell Growth Differ..

[21] Kanezaki R., Toki T., Yokoyama M., Yomogida K., Sugiyama K., Yamamoto M., Igarashi K., Ito E. (2001). Transcription Factor
BACH1 Is Recruited to the Nucleus by Its Novel Alternative Spliced
Isoform∗. J. Biol. Chem..

[22] Afzali B. (2017). BACH2 immunodeficiency
illustrates an association between super-enhancers
and haploinsufficiency. Nature Immunology.

[23] Liu J., So̷rensen A., Boshi W., Wabl M., Nielsen A., Pedersen F. (2009). Identification
of novel Bach2 transcripts and protein
isoforms through tagging analysis of retroviral integrations in B-cell
lymphomas. BMC Mol. Biol..

[24] Rosbrook G. O., Stead M. A., Carr S. B., Wright S. C. (2012). The structure of
the Bach2 POZ-domain dimer reveals an intersubunit disulfide bond. Acta Crystallographica Section D.

[25] Igarashi K., Kurosaki T., Roychoudhuri R. (2017). BACH transcription
factors in innate
and adaptive immunity. Nat. Rev. Immunol..

[26] Ferreira M. (2011). Identification of IL6R and chromosome 11q13.5
as risk loci for asthma. Lancet.

[27] Sawcer S. (2011). Genetic risk and a primary
role for cell-mediated immune mechanisms
in multiple sclerosis. Nature.

[28] Franke A. (2010). Genome-wide meta-analysis increases to 71 the number
of confirmed
Crohn’s disease susceptibility loci. Nat. Genet..

[29] Medici M. (2014). Identification of Novel
Genetic Loci Associated with Thyroid Peroxidase
Antibodies and Clinical Thyroid Disease. PLoS
Genet..

[30] Roychoudhuri R. (2013). BACH2 represses effector
programs to stabilize T­(reg)-mediated immune
homeostasis. Nature.

[31] Kim E. H., Gasper D. J., Lee S. H., Plisch E. H., Svaren J., Suresh M. (2014). Bach2 Regulates Homeostasis of Foxp3+ Regulatory T
Cells and Protects against Fatal Lung Disease in Mice. J. Immunol..

[32] Roychoudhuri R., Eil R. L., Restifo N. P. (2015). The interplay of
effector and regulatory
T cells in cancer. Current Opinion in Immunology.

[33] Roychoudhuri R. (2016). The transcription factor
BACH2 promotes tumor immunosuppression. J. Clin.
Invest..

[34] Sakaguchi S., Sakaguchi N., Asano M., Itoh M., Toda M. (1995). Immunologic
self-tolerance maintained by activated T cells expressing IL-2 receptor
alpha-chains (CD25). Breakdown of a single mechanism of self-tolerance
causes various autoimmune diseases. Journal
of immunology (Baltimore, Md.: 1950).

[35] Zhang H., Dai D., Hu Q., Yang F., Xue Y., Li F., Shen N., Zhang M., Huang C. (2021). Bach2 attenuates IL-2R
signaling to control Treg homeostasis and Tfr development. Cell Reports.

[36] Yao C. (2021). BACH2 enforces the transcriptional and epigenetic programs
of stem-like
CD8+ T cells. Nature immunology.

[37] Gao H. (2025). BACH2 promotes seeding
and establishment of long-lived HIV-1 reservoir
in memory CD4+ T cells. Cell Reports Medicine.

[38] Son J., Ding H., Farb T., Efanov A., Sun J., Gore J., Syed S., Lei Z., Qidi W., Accili D., Califano A. (2021). BACH2 inhibition reverses
β
cell failure in type 2 diabetes models. J. Clin.
Invest..

[39] Attucks O. C., Jasmer K. J., Hannink M., Kassis J., Zhong Z., Gupta S., Victory S. F., Guzel M., Polisetti D. R., Andrews R., Mjalli A. M. M., Kostura M. J. (2014). Induction of Heme
Oxygenase I (HMOX1) by HPP-4382: A Novel Modulator of Bach1 Activity. PLoS One.

[40] Vardaka P., Lozano T., Bot C., Ellery J., Whiteside S. K., Imianowski C. J., Farrow S., Walker S., Okkenhaug H., Yang J., Okkenhaug K., Kuo P., Roychoudhuri R. (2020). A cell-based
bioluminescence assay reveals dose-dependent and contextual repression
of AP-1-driven gene expression by BACH2. Sci.
Rep..

[41] London N., Raveh B., Movshovitz-Attias D., Schueler-Furman O. (2010). Can self-inhibitory
peptides be derived from the interfaces of globular protein–protein
interactions?. Proteins: Struct., Funct., Bioinf..

[42] Sedan Y., Marcu O., Lyskov S., Schueler-Furman O. (2016). Peptiderive
server: derive peptide inhibitors from protein–protein interactions. Nucleic Acids Res..

[43] Lyskov S. (2013). Serverification of Molecular Modeling Applications: The Rosetta Online
Server That Includes Everyone (ROSIE). PLoS
One.

[44] Schymkowitz J., Borg J., Stricher F., Nys R., Rousseau F., Serrano L. (2005). The FoldX web server: an online force
field. Nucleic Acids Res..

[45] Delgado J., Radusky L. G., Cianferoni D., Serrano L. (2019). FoldX 5.0: working
with RNA, small molecules and a new graphical interface. Bioinformatics.

[46] Kozakov D., Hall D. R., Xia B., Porter K. A., Padhorny D., Yueh C., Beglov D., Vajda S. (2017). The ClusPro
web server
for protein–protein docking. Nat. Protoc..

[47] Lamiable A., Thévenet P., Rey J., Vavrusa M., Derreumaux P., Tufféry P. (2016). PEP-FOLD3:
faster de novo structure prediction for
linear peptides in solution and in complex. Nucleic Acids Res..

[48] Thévenet P., Shen Y., Maupetit J., Guyon F., Derreumaux P., Tufféry P. (2012). PEP-FOLD: an updated de novo structure prediction server
for both linear and disulfide bonded cyclic peptides. Nucleic Acids Res..

[49] Shen Y., Maupetit J., Derreumaux P., Tufféry P. (2014). Improved PEP-FOLD
Approach for Peptide and Miniprotein Structure Prediction. J. Chem. Theory Comput..

[50] Adasme M. F., Linnemann K. L., Bolz S. N., Kaiser F., Salentin S., Haupt V., Schroeder M. (2021). PLIP 2021: expanding the scope of
the protein–ligand interaction profiler to DNA and RNA. Nucleic Acids Res..

[51] Honorato R. V., Koukos P. I., Jiménez-García B., Tsaregorodtsev A., Verlato M., Giachetti A., Rosato A., Bonvin A. M. J. J. (2021). Structural Biology in the Clouds:
The WeNMR-EOSC Ecosystem. Front. Mol. Biosci..

[52] van
Zundert G., Rodrigues J., Trellet M., Schmitz C., Kastritis P., Karaca E., Melquiond A., van Dijk M., de Vries S., Bonvin A. (2016). The HADDOCK2.2 Web
Server: User-Friendly Integrative Modeling of Biomolecular Complexes. J. Mol. Biol..

[53] Jurrus E. (2018). Improvements to the APBS biomolecular solvation software
suite. Protein Sci..

[54] MacKerell A. D. J. (1998). All-Atom Empirical Potential
for Molecular Modeling
and Dynamics Studies of Proteins. J. Phys. Chem.
B.

[55] Mackerell A. D., Feig M., Brooks C. L. (2004). Extending the
treatment of backbone energetics in protein force fields:
Limitations of gas-phase quantum mechanics in reproducing protein
conformational distributions in molecular dynamics simulations. J. Comput. Chem..

[56] Best R. B., Zhu X., Shim J., Lopes P. E. M., Mittal J., Feig M., MacKerell A. D. J. (2012). Optimization of the Additive CHARMM All-Atom Protein
Force Field Targeting Improved Sampling of the Backbone φ, ψ
and Side-Chain χ1 and χ2 Dihedral Angles. J. Chem. Theory Comput..

[57] Huang J., Rauscher S., Nawrocki G., Ting R., Feig M., de Groot B., Grubmüller H., MacKerell A. (2017). CHARMM36m:
An Improved Force Field for Folded and Intrinsically Disordered Proteins. Biophys. J..

[58] Jorgensen W. L., Madura J. D. (1983). Quantum and statistical
mechanical studies of liquids.
25. Solvation and conformation of methanol in water. J. Am. Chem. Soc..

[59] Martyna G. J., Hughes A., Tuckerman M. E. (1999). Molecular dynamics algorithms for
path integrals at constant pressure. J. Chem.
Phys..

[60] Hoover W.
G. (1985). Canonical
dynamics: Equilibrium phase-space distributions. Phys. Rev. A.

[61] Humphrey W., Dalke A., Schulten K. (1996). VMD: Visual molecular dynamics. J. Mol. Graphics.

[62] Bakan A., Meireles L. M., Bahar I. (2011). ProDy: Protein Dynamics
Inferred
from Theory and Experiments. Bioinformatics.

[63] Bakan A., Dutta A., Mao W., Liu Y., Chennubhotla C., Lezon T. R., Bahar I. (2014). Evol and ProDy for
bridging protein
sequence evolution and structural dynamics. Bioinformatics.

[64] Zhang S., Krieger J. M., Zhang Y., Kaya C., Kaynak B., Mikulska-Ruminska K., Doruker P., Li H., Bahar I. (2021). ProDy 2.0:
increased scale and scope after 10 years of protein dynamics modelling
with Python. Bioinformatics.

[65] Miller B. R. I., McGee T. D. J., Swails J. M., Homeyer N., Gohlke H., Roitberg A. E. (2012). MMPBSA.py: An Efficient
Program for End-State Free
Energy Calculations. J. Chem. Theory Comput..

[66] Crowley M. F., Williamson M. J., Walker R. C. (2009). CHAMBER: Comprehensive support for
CHARMM force fields within the AMBER software. Int. J. Quantum Chem..

[67] Suenaga T., Watanabe-Matsui M., Uejima T., Shima H., Matsui T., Ikeda-Saito M., Shirouzu M., Igarashi K., Murayama K. (2016). Charge-state-distribution
analysis of Bach2 intrinsically disordered heme binding region. Journal of Biochemistry.

[68] Pollard T. D. (2010). A Guide
to Simple and Informative Binding Assays. Mol.
Biol. Cell.

[69] Laskowski R. A., Swindells M. B. (2011). LigPlot+: Multiple Ligand–Protein Interaction
Diagrams for Drug Discovery. J. Chem. Inf. Model..

[70] Wallace A. C., Laskowski R. A., Thornton J. M. (1995). LIGPLOT: a program to generate schematic
diagrams of protein-ligand interactions. Protein
Engineering, Design and Selection.

[71] Chauhan A., Tikoo A., Kapur A. K., Singh M. (2007). The taming of the cell
penetrating domain of the HIV Tat: Myths and realities. J. Controlled Release.

[72] Ito N., Watanabe-Matsui M., Igarashi K., Murayama K. (2009). Crystal structure
of
the Bach1 BTB domain and its regulation of homodimerization. Genes to Cells.

[73] Brennan A., Leech J. T., Kad N. M., Mason J. M. (2022). An Approach to Derive
Functional Peptide Inhibitors of Transcription Factor Activity. JACS Au.

[74] Madden S. K., Brennan A., Mason J. M. (2024). A library-derived peptide inhibitor
of the BZLF1 transcription factor. J. Pept.
Sci..

[75] Birts C. N., Nijjar S. K., Mardle C. A., Hoakwie F., Duriez P. J., Blaydes J. P., Tavassoli A. (2013). A cyclic peptide inhibitor of C-terminal
binding protein dimerization links metabolism with mitotic fidelity
in breast cancer cells. Chem. Sci..

[76] Turkson J., Kim J. S., Zhang S., Yuan J., Huang M., Glenn M., Haura E., Sebti S., Hamilton A. D., Jove R. (2004). Novel peptidomimetic
inhibitors of signal transducer and activator
of transcription 3 dimerization and biological activity. Molecular Cancer Therapeutics.

[77] Lozano T., Casares N., Martil-Otal C., Anega B., Gorraiz M., Parker J., Ruiz M., Belsúe V., Pineda-Lucena A., Oyarzabal J., Lasarte J. J. (2021). Searching for Peptide
Inhibitors of T Regulatory Cell Activity by Targeting Specific Domains
of FOXP3 Transcription Factor. Biomedicines.

[78] Timucin A. C. (2021). Structure
based peptide design, molecular dynamics and MM-PBSA studies for targeting
C terminal dimerization of NFAT5 DNA binding domain. Journal of Molecular Graphics and Modelling.

[79] Genheden S., Ryde U. (2015). The MM/PBSA and MM/GBSA
methods to estimate ligand-binding affinities. Expert Opinion on Drug Discovery.

[80] Wang C., Greene D., Xiao L., Qi R., Luo R. (2018). Recent Developments
and Applications of the MMPBSA Method. Front.
Mol. Biosci..

[81] Hu X., Contini A. (2019). Rescoring Virtual Screening Results with the MM-PBSA
Methods: Beware of Internal Dielectric Constants. J. Chem. Inf. Model..

[82] Krautwald S., Dewitz C., Fändrich F., Kunzendorf U. (2016). Inhibition
of regulated cell death by cell-penetrating peptides. Cell. Mol. Life Sci..

[83] Watson J. L. (2023). De Novo Design of Protein Structure and Function with RFdiffusion. Nature.

[84] Dauparas J. (2022). Robust Deep Learning–Based Protein Sequence
Design Using ProteinMPNN. Science.

[85] Pacesa M. (2025). One-Shot Design of Functional
Protein Binders with BindCraft. Nature.

[86] Alli R., Youngblood B. (2026). BACH2 the
Future. Nat. Immunol..

[87] Hu T., Zhu Z., Luo Y., Wizzard S., Hoar J., Shinde S. S., Yihunie K., Yao C., Wu T. (2026). BACH2 Dosage Establishes
the Hierarchy of Stemness and Fine-Tunes Antitumor Immunity in CAR
T Cells. Nat. Immunol..

[88] Conti A. G. (2026). Fine-Tuning BACH2 Dosage
Balances Stemness and Effector Function
to Enhance Antitumor T Cell Therapy. Nat. Immunol..

[89] Chang T.-C. (2026). BACH2 Regulates T Cell
Lineage State to Enhance CAR T Cell Function. Nat. Immunol..

