# Supplementary material for: Peptide Design for Targeting BTB Domain Homodimerization of BACH2: Complementary In Silico and In Vitro Approaches
Source: ACS Omega. 2026 Apr 17;11(16):24679–704. doi: 10.1021/acsomega.6c01122 (PMC13129818; doi:10.1021/acsomega.6c01122)
Supplement: Supplementary file 1 [file ao6c01122_si_001.pdf]

Peptide design for targeting BTB domain homodimerization of BACH2: Complementary *in silico* and *in vitro* approaches

Efe Acar[a], Hüveyda Başağa[b], Emel Timuçin[c], Ahmet Can Timuçin[d,e,*]

[a]*Department of Molecular Biology and Genetics, Graduate School of Natural and Applied Sciences, Acibadem Mehmet Ali Aydinlar University, İstanbul, 34752, Türkiye*
[b]*Department of Molecular Biology, Genetics and Bioengineering, Faculty of Engineering and Natural Sciences, Sabancı University, İstanbul, 34956, Türkiye*
[c]*Department of Molecular Biology and Genetics, Faculty of Science, Gebze Technical University, Kocaeli, 41400, Türkiye*
[d]*Department of Molecular Biology and Genetics, Faculty of Engineering and Natural Sciences, Acibadem Mehmet Ali Aydinlar University, İstanbul, 34752, Türkiye*
[e]*Department of Molecular Biology and Genetics, Graduate School of Natural and Applied Sciences, Acibadem Mehmet Ali Aydinlar University, İstanbul, 34752, Türkiye*

Supporting Information

*Corresponding author
Email address: ahmet.timucin@acibadem.edu.tr (Ahmet Can Timuçin)

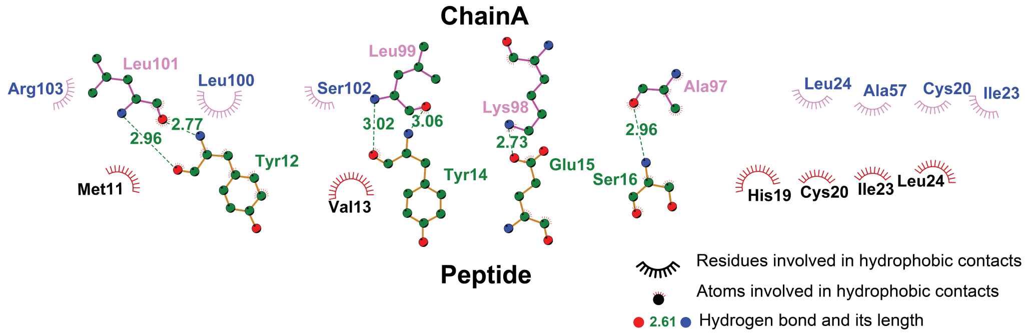

Supplementary Figure 1: The possible hydrogen bonds and hydrophobic contacts between the BACH2 BTB domain monomer sequence in chain A, predicted to mediate protein-peptide interaction, and the peptide sequence predicted on chain B were analyzed using the Ligplot+ [1, 2] program on the native structure of BACH2 BTB homodimer. The BACH2 BTB domain monomer of chain A, within the sequence range A97-A103, predominantly interacted through hydrogen bonds with the amino acids in the Met11-Ser16 range of the predicted peptide sequence on chain B. Other amino acids predicted on chain A (Ile23, Leu24, Cys20, and Ala57) established only hydrophobic contacts with certain amino acids (His19, Cys20, Ile23, and Leu24) of the predicted peptide sequence on chain B. Explanations for the representations used in Ligplot+ analysis were provided at the bottom right of the figure. The amino acid numbering was based on the original structure of the BACH2 BTB domain homodimer (PDB ID: 3OHU).

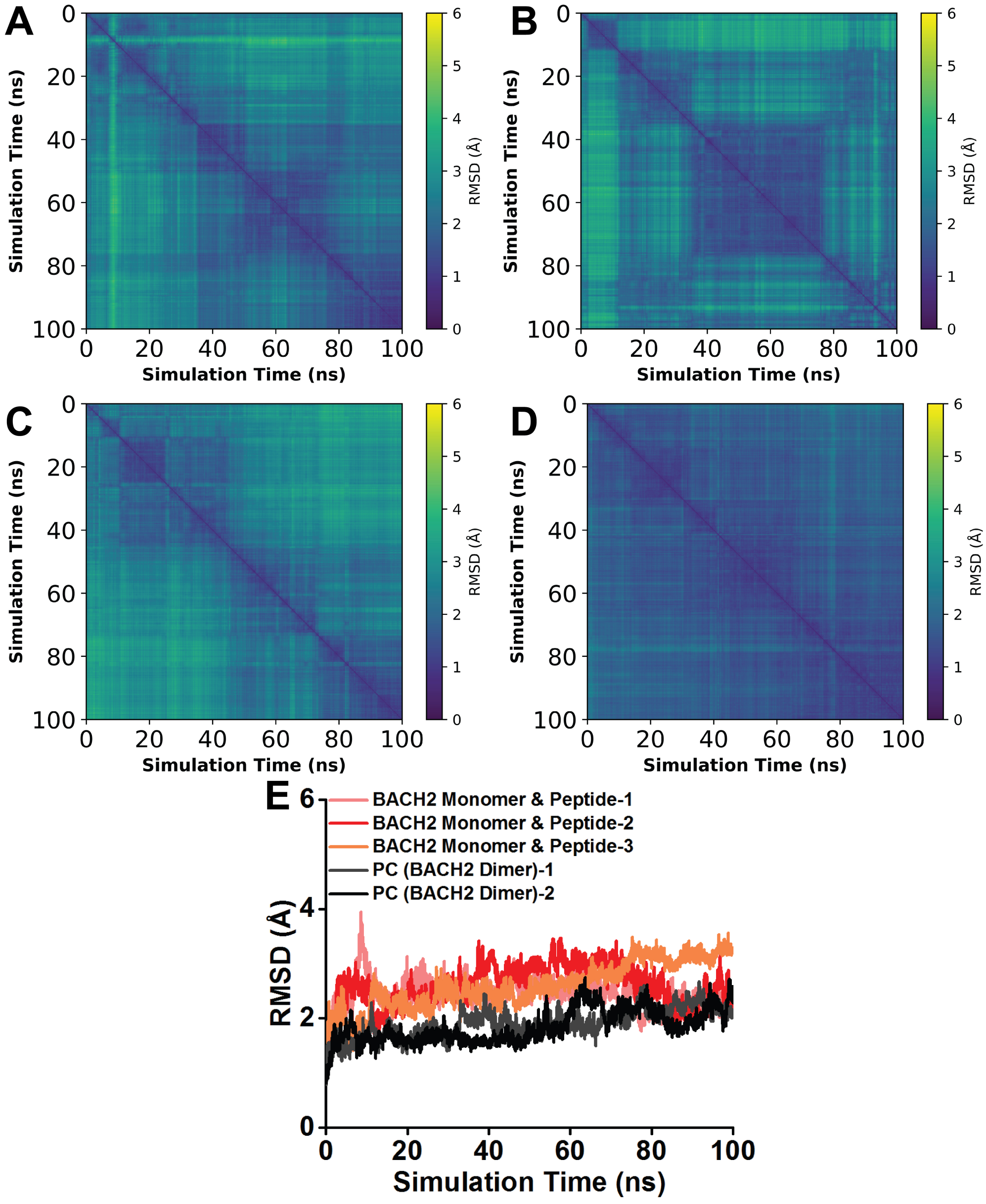

Supplementary Figure 2: RMSD analyses for the initial MD simulations implemented for peptide design. A), B), C) All-to-all RMSD graphs show that the BACH2 BTB domain monomer-preliminary peptide complex exhibited similar conformations (RMSD < ~3 Å) after the first 10th ns of the 100 ns MD simulations (shown in A, B, and C for replicates 1, 2, and 3, respectively). D) All-to-all RMSD analysis of the homodimeric BACH2 BTB domain structure used as a positive control (PC) shows that it maintained similar conformations throughout the 100 ns simulation, with RMSD remaining below ~3 Å throughout the entire simulation. This control simulation was repeated twice and since similar results were observed, only the results from the first replicate were shown. E) RMSD-time graphs for the simulations analyzed in A, B, C, and C, along with the second replicate of the positive control (PC (BACH2 Dimer)-2), indicated that RMSD fluctuations remained below ~3 Å during the 100 ns MD simulations. When the results from all-to-all RMSD and RMSD-time graphs are evaluated together, it was concluded that all simulations after the first 10th ns are suitable

Supplementary Figure 2: (Continued) for more detailed analysis. The protein-peptide complex simulations were repeated three times and the positive control BACH2 BTB domain homodimer simulations were repeated twice.

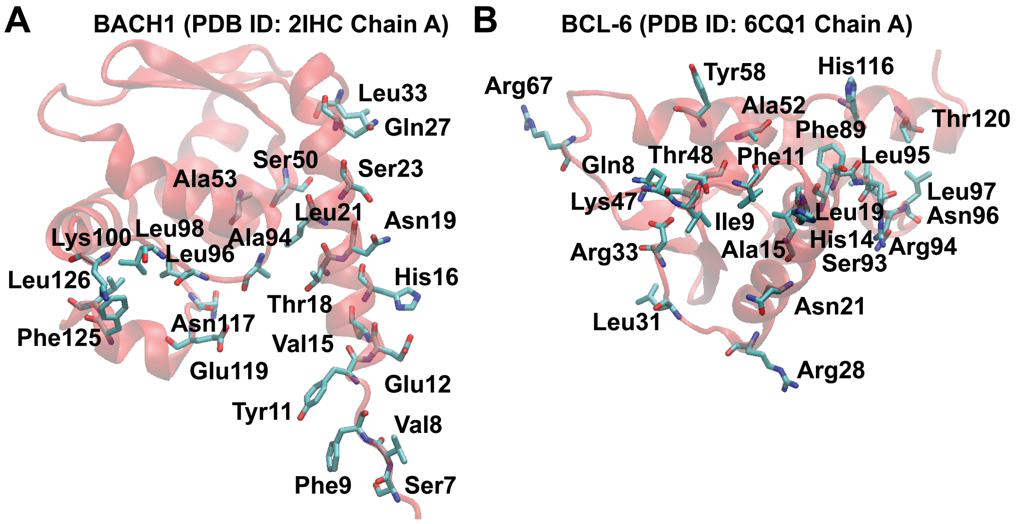

Supplementary Figure 3: Possible amino acid networks governing dimerization for the potential negative controls BACH1 and BCL6 BTB domain dimers, identified using the PLIP program [3]. A), B) Potential negative controls, the BACH1 BTB domain dimer (PDB ID: 2IHC) and the BCL-6 BTB domain dimer (PDB ID: 6CQ1), which were utilized to check whether if peptides could specifically bind to the BACH2 BTB domain, were also analyzed in terms of the amino acids residing in target side of the dimer interface using the PLIP algorithm. For both, the amino acids holding these complexes together were identified and amino acids from the target monomer were depicted in the figure. In the next step, peptides that would target these amino acids were analyzed using the ClusPro global docking program.

Supplementary Table 1: Weak interaction analyses of ClusPro based global docking generated structures of the peptide 13 in complex with the dimerization regions of BACH1 BTB domain and BCL-6 BTB domain monomers.

Target Monomer	Interaction Type	Protein AA#[1]	Peptide AA#	Distance (Å)	Side Chain Interaction
BACH1	Hydrophobic	Val8	Ile26	3.87	-
	Hydrogen bond	Arg49	Thr20	2.85	No
		Ala10	Leu27	3.73	No
BCL-6	Hydrophobic	Phe89	Ile26	3.75	-
		Ile9	Ile26	3.94	-
		Ile9	Leu27	3.71	-
	Hydrogen bond	Asp6	Arg13	3.83	Yes
		His116	Tyr15	2.78	Yes
		Arg94	Glu19	2.76	No
		His116	Thr24	3.87	Yes
		Thr120	Arg25	2.82	Yes
	Salt bridge	Asp6	Arg13	3.18	-

[1] Amino acid residues residing +3 and -3 of the PLIP defined interaction surface.

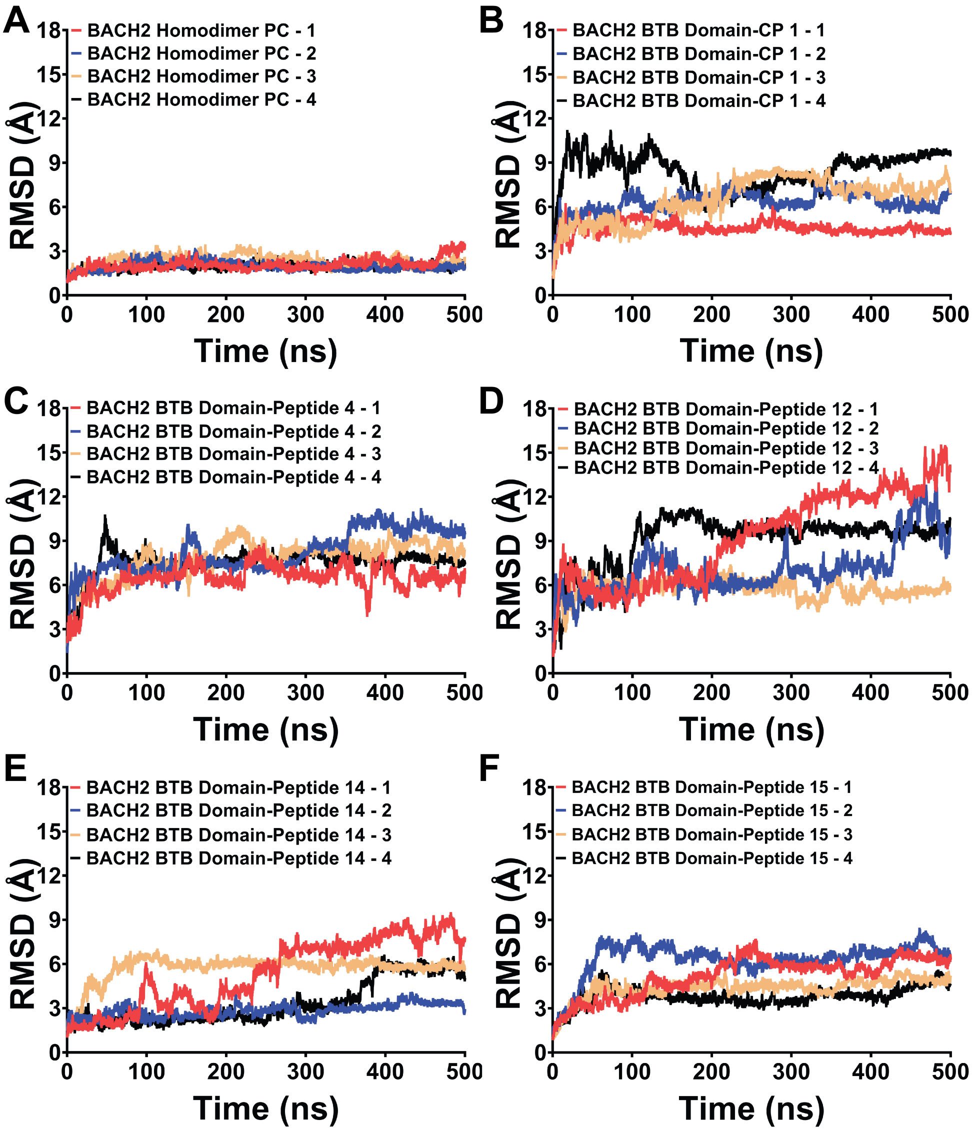

Supplementary Figure 4: Time-dependent RMSD analysis for four 500 ns MD simulation replicates of the positive control BACH2 BTB domain homodimer (A), BACH2 BTB domain-Control Peptide (CP) (Peptide 1, Wild type) (B), BACH2 BTB domain-Peptide 4 (C), and BACH2 BTB domain-Peptide 12 (D), BACH2 BTB domain-Peptide 14 (E), BACH2 BTB domain-Peptide 15 (F) complexes. In all complexes, C, N, Cα, and O atoms were used as the protein backbone atoms for RMSD calculations. (A) The positive control BACH2 BTB domain homodimer exhibited fluctuations below 3 Å, maintaining conformational stability throughout all four 500 ns simulation replicates. (B) In the BACH2 BTB domain-Control Peptide (Peptide 1) complex, the first and second replicates reached a stable conformation after 20 ns. However, the third and fourth replicates only exhibited fluctuations below 3 Å in the last 250 ns, indicating that conformational stability was achieved in this phase. (C) In the BACH2 BTB domain-Peptide 4 simulations, all replicates showed fluctuations below 3 Å after the first 50 ns, suggesting that conformational stability was achieved from this point onward. (D) In the BACH2 BTB domain-Peptide 12 complex, the first replicate failed to reach conformational stability. The second replicate attempted to explore a different conformation in the last 100 ns. Although the third and fourth replicates maintained a stable conformation with fluctuations below 3 Å in the last 400 ns, the lack of sufficient stable

Supplementary Figure 4: (Continued) conformational replicates led to the elimination of this peptide from further analysis at this point. (E) The BACH2 BTB domain-Peptide 14 complex showed fluctuations below 3 Å after the first 250 ns in the first replicate, indicating conformational stability in this phase. In the other replicates, stability was achieved after the first 50 ns, with RMSD fluctuations remaining below 3 Å for the rest of the simulation (F) The BACH2 BTB domain-Peptide 15 complex maintained fluctuations below 3 Å for the last 450 ns in all simulation replicates, suggesting that the complex remained structurally stable throughout most of the simulation.

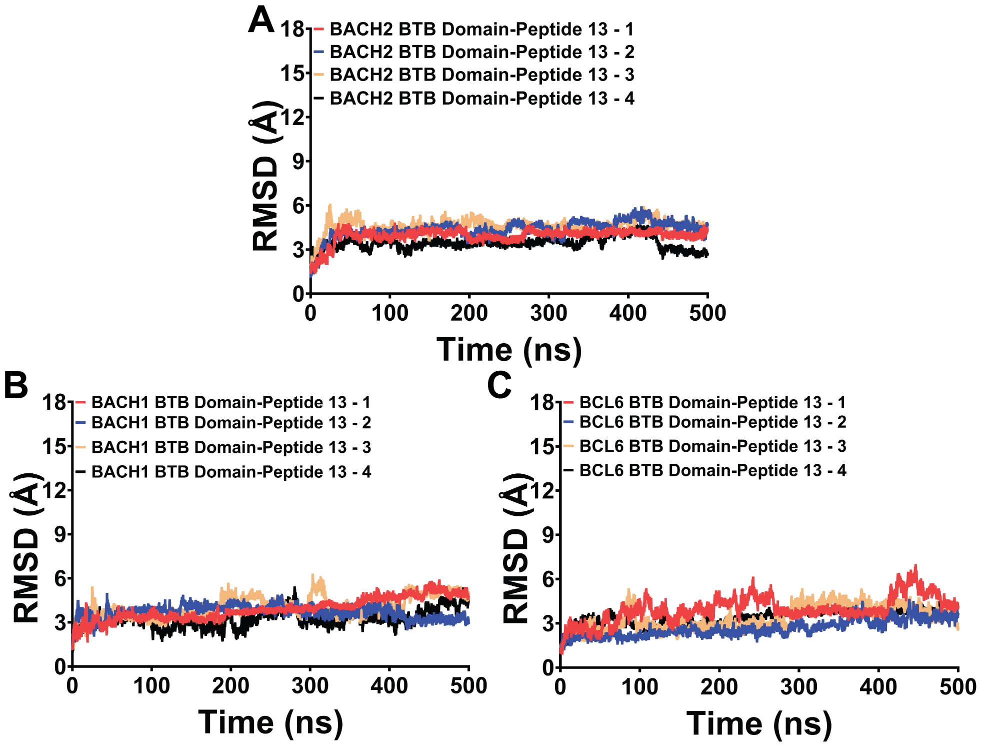

Supplementary Figure 5: Time-dependent RMSD analysis for four 500 ns MD simulation replicates of the following complexes: BACH2 BTB domain-Peptide 13 (A), BACH1 BTB domain-Peptide 13 (B), and BCL6 BTB domain-Peptide 13 (C). In all complexes, C, N, Cα, and O atoms were considered as the protein backbone atoms.(A, B, C) The BACH2 BTB domain-Peptide 13 complex (A), along with its potential negative control BTB domain monomer complexes, BACH1 (D) and BCL6 (E), exhibited fluctuations below 3 Å after the first 20 ns. Throughout the final 480 ns of all four 500 ns simulation replicates, these complexes remained conformationally stable.

Supplementary Table 2: Carbon-carbon contacts with the highest occupancy rates observed in the last 250 ns of simulations for the BACH2 BTB domain homodimer PC complex, BACH2 BTB domain-control peptide 1 (CP1), and BACH2 BTB domain-peptide 13 complex (P13).

**PC-1**		**Occ. (%)**	**CP1-1**		**Occ. (%)**	**P13-1**		**Occ. (%)**
**Y12**	**L101**	**100**	**M11**	**R10**	**54.2**	**N120**	**R13**	**100**
**Y14**	**K98**	**95.3**	Y12	R10	7.8	**Y12**	**R9**	**100**
**K98**	**Y14**	**93.4**	Y14	R10	7.8	**N120**	**V16**	**99.4**
**N120**	**Y14**	**85.7**	V13	R10	0.2	**K98**	**V16**	**90.8**
**PC-2**		**Occ. (%)**	**CP1-2**		**Occ. (%)**	**P13-2**		**Occ. (%)**
**Y12**	**L101**	**99.9**	**K98**	**M14**	**90.6**	**K98**	**V16**	**99.4**
**N120**	**Y14**	**98.8**	M11	M14	4.9	**N120**	**R13**	**83.1**
**Y14**	**K98**	**95.8**	ND	ND	ND	N120	V16	21.8
**K98**	**Y14**	**93.9**	ND	ND	ND	Y12	V16	2.9
**PC-3**		**Occ. (%)**	**CP1-3**		**Occ. (%)**	**P13-3**		**Occ. (%)**
**Y12**	**L101**	**99.8**	**V13**	**R10**	**99.4**	**N120**	**V16**	**93.1**
**Y14**	**K98**	**97.1**	N120	M14	39.8	**K98**	**V16**	**79.3**
**N120**	**Y14**	**97.1**	K98	M14	5.2	Y12	R9	25
**K98**	**Y14**	**89.4**	Y12	R10	2.2	N120	R13	7.1
**PC-4**		**Occ. (%)**	**CP1-4**		**Occ. (%)**	**P13-4**		**Occ. (%)**
**K98**	**Y14**	**93**	**M11**	**M14**	**56**	**N120**	**R13**	**99.7**
**Y12**	**L101**	**99.7**	Y14	M14	43.5	**N120**	**V16**	**99**
**N120**	**Y14**	**95.5**	K98	L27	30.3	**K98**	**V16**	**96.5**
**Y14**	**K98**	**94.9**	N120	M14	8.7	Y14	R9	0.5

For PC, carbon-carbon contacts were searched in between MM-PBSA defined residues between each monomer. For peptide containing complexes, MM-PBSA defined residues of peptides were searched against MM-PBSA defined residues of chain A target monomer of PC due to the fact that control peptide 1 containing complex lacks target MM-PBSA defined amino acids. A minimum occupancy rate of 50% was used as the criterion for selecting carbon-carbon contacts for analysis (Highlighted in bold). The analyses were performed using the `newcontact.tcl` script. Carbon to carbon contact selection criteria was based on capability of showing distances under 5 Å (The first four highest occupancy carbon-carbon contacts were selected for the analysis. Except from occupacy columns, first letter stands for one letter amino acid abbreviation, numerical values indicates residue number). ND: No data available.

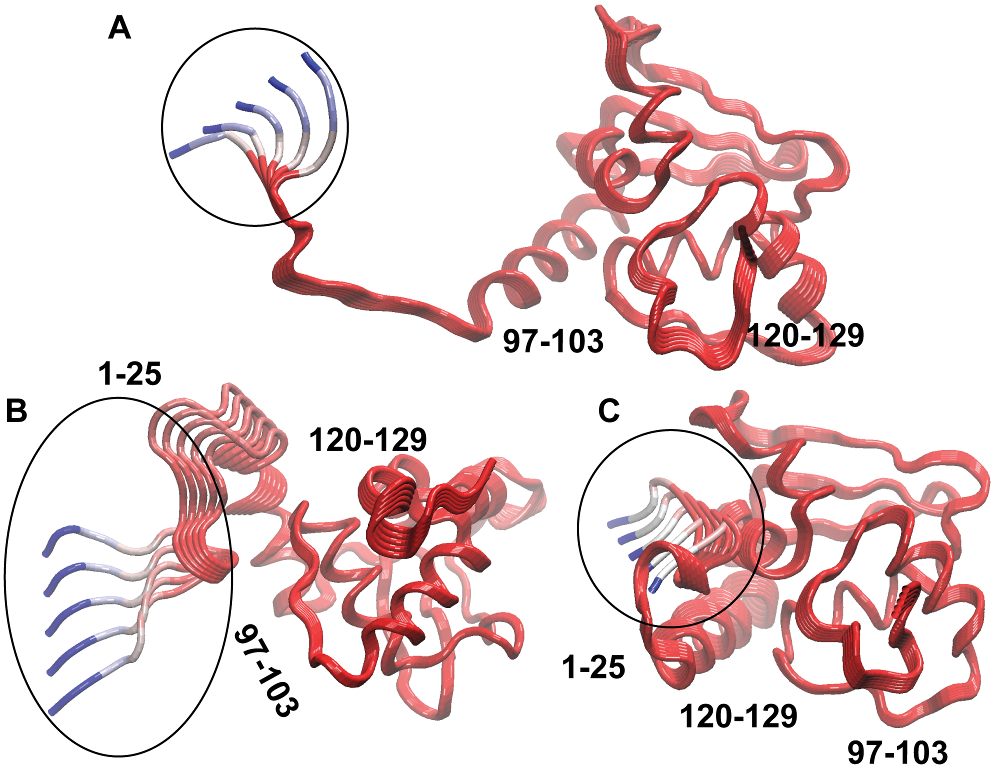

Supplementary Figure 6: Shortened trajectories along the first principal component from the essential dynamics (PCA) analysis of the third replicate simulations for the positive control BACH2 BTB domain homodimer, the BACH2 BTB domain monomer–control peptide 1 complex, and the BACH2 BTB domain monomer–peptide 13 complexes. Cα atom displacements were colored from red to white to blue, indicating increasing mobility, based on projections along the first principal component from the third replicate of each simulation group. A) BACH2 BTB domain monomer from the positive control complex, B) BACH2 BTB domain monomer in the control peptide 1-containing complex, C) BACH2 BTB domain monomer in the peptide 13-containing complex. In each structure, the N-terminal region (residues 1–25), the segment corresponding to residues 97–103 (region interacting with the predicted wild-type peptide), and the C-terminal region (residues 120–129), which includes residues contributing to the Peptide 13 interaction, were shown via residue numbers. In the positive control complex, N-terminal stability was maintained through interactions with the C-terminal region of the second monomer of the BACH2 BTB homodimer. As a result, despite appearing spatially distant from the protein core, the N-terminus remained conformationally restricted. In the Peptide 13 complex, the reduction in N-terminal mobility was associated with its spatial proximity to the C-terminal region. In contrast, in the Peptide 1 complex, the N-terminal region remained relatively more flexible and did not approach the functionally relevant C-terminal region as closely as in the Peptide 13 complex. Since similar motion patterns were observed across other simulation replicas and eigenvectors, only the first eigenvector from the third replicate simulations was presented here.

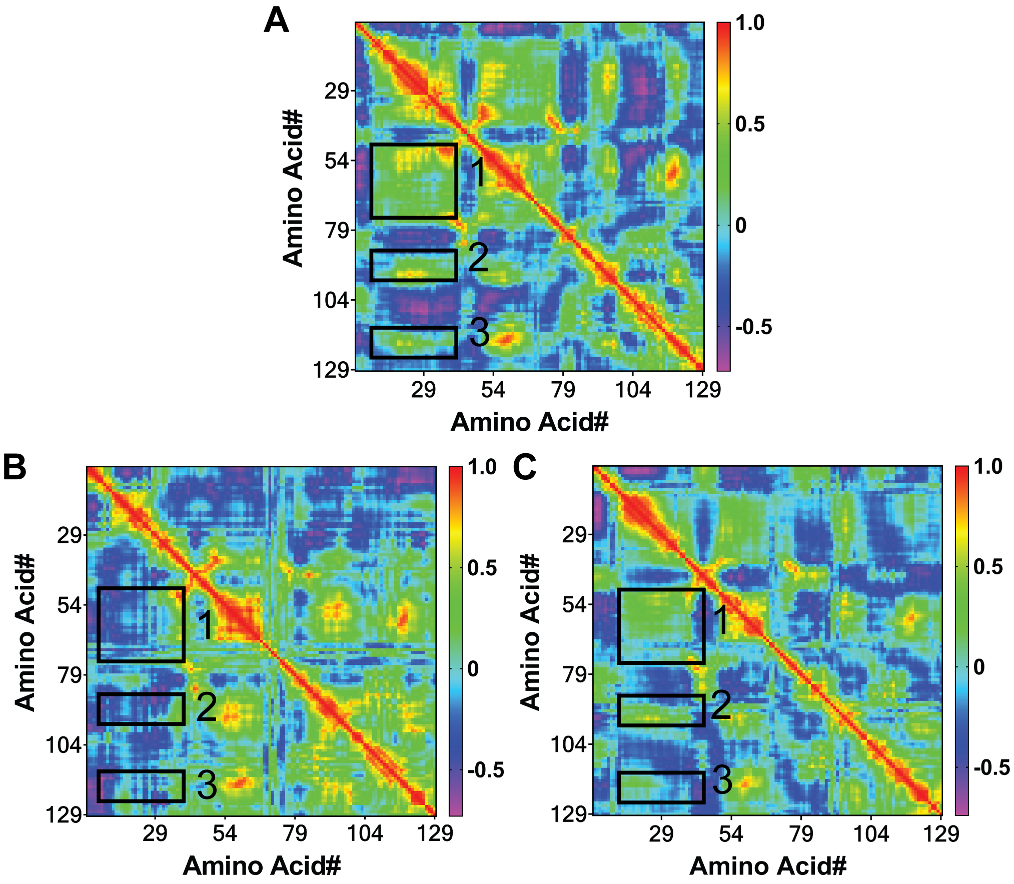

Supplementary Figure 7: Cross-correlation analysis of BACH BTB monomer retrieved from the third replicate simulations for the positive control BACH2 BTB domain homodimer, the BACH2 BTB domain monomer–control peptide 1 and the BACH2 BTB domain monomer–peptide 13 complexes. The final 250 ns of the third replicate from each simulation was used in the analysis. A) BACH2 BTB domain monomer from the positive control complex, B) BACH2 BTB domain monomer from the control peptide 1-containing complex, C) BACH2 BTB domain monomer from the peptide 13-containing complex. Similar to the positive control, the N-terminal region of the BACH2 BTB domain monomer in the peptide 13-containing complex exhibited positive correlations, particularly in the boxed regions. In contrast, the same regions in the control peptide 1-containing complex mostly showed a shift toward negative correlations. This observation suggested that the BACH2 BTB domain–peptide 13 interaction contributed to maintaining the N-terminal dynamic profile of the BACH2 monomer in a manner similar to that observed in the positive control complex. In the correlation heatmap, red indicates stronger positive correlation, while blue represents negative correlation.

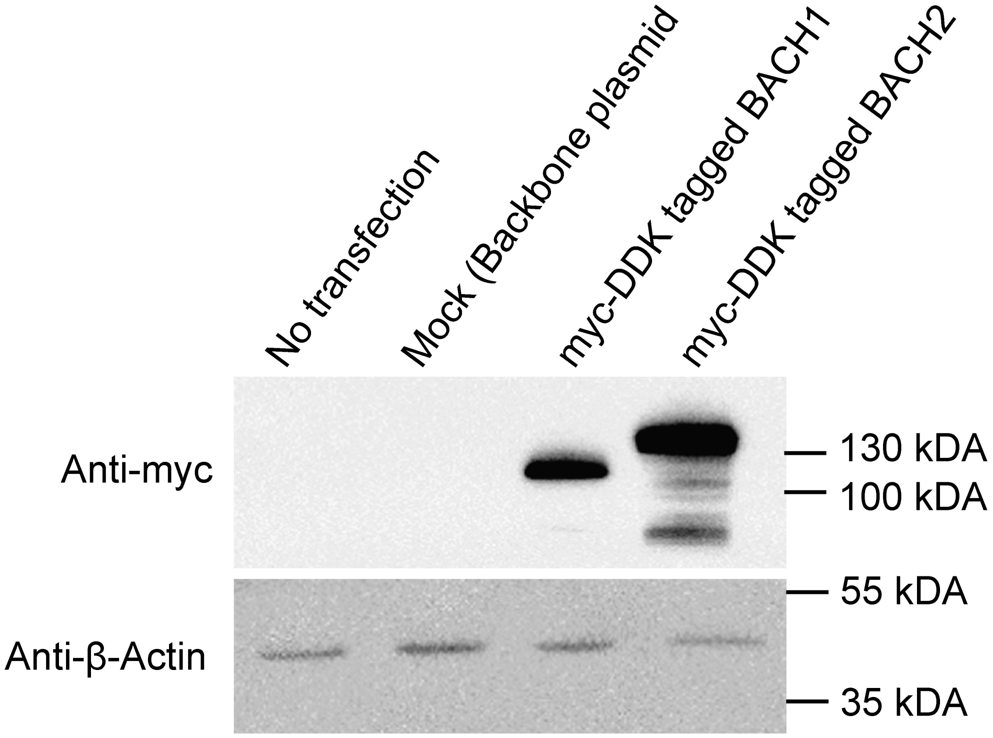

Supplementary Figure 8: Verification of myc-DDK-tagged BACH1 and BACH2 overexpression in HEK293T cells via immunoblotting. To confirm the overexpression of myc-DDK-tagged BACH1 and BACH2 in HEK293T cells and the subsequent preparation of supernatants, an immunoblotting analysis was performed. A total of 20 µg of protein was used from each experimental group for this analysis. Overexpression was assessed using an anti-myc antibody directed against the myc epitope tag. As expected, no anti-myc signal was detected at the corresponding molecular weight in lysates from non-transfected HEK293T cells (no transfection) or those transfected with only the backbone plasmid (Mock). In contrast, clear anti-myc signals were observed at the expected molecular weights in lysates from cells overexpressing full-length myc-DDK-tagged BACH1 and myc-DDK-tagged BACH2, confirming successful expression of both constructs. To ensure equal protein loading across samples, membranes were also probed with an anti-β-Actin antibody as a loading control. These results collectively confirmed that the overexpression experiments were successfully carried out.

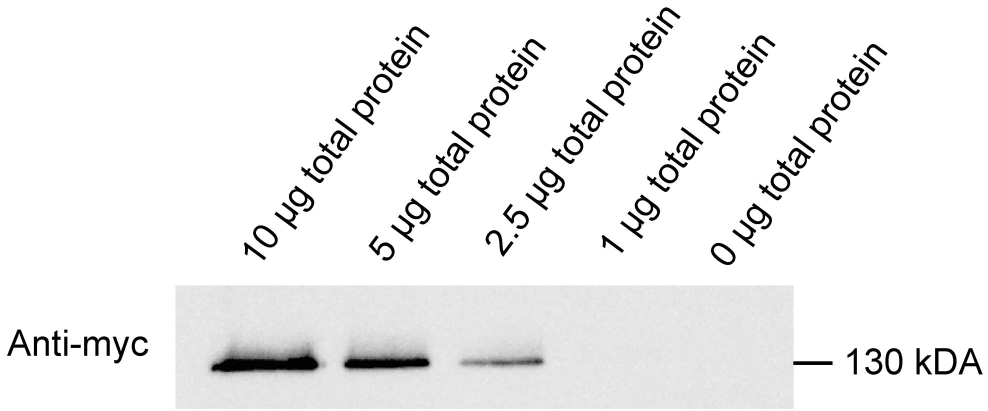

Supplementary Figure 9: Optimization experiment to determine the minimum protein amount required for detectable gel bands following immunoblotting of overexpressed myc-DDK–tagged BACH2. Total protein containing myc-DDK–tagged BACH2 was subjected to immunoblotting with an anti-myc antibody. Bands were clearly detectable on the gel with a minimum of approximately 2.5 μg protein. This optimization was used as the basis for assessing the reduction of myc signal in the peptide-bound supernatant. Accordingly, after execution of supernatant depletion assay, ~2.3 μg of protein from each sample was loaded per well to maximize the resolution of the peptide's effect.

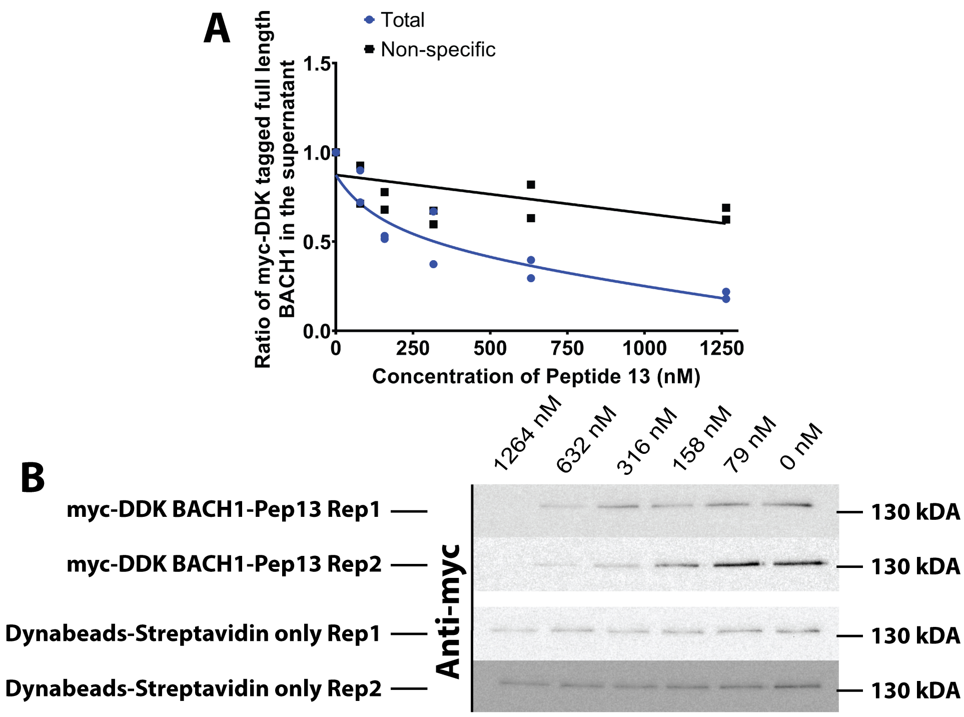

Supplementary Figure 10: Determination of the dissociation constant of peptide 13 against Myc-DDK tagged BACH1 by supernatant depletion analysis. In each Eppendorf tube, a total of 7.5 μg of total protein extract containing Myc-DDK tagged BACH1 overexpression was subjected to Dynabeads-streptavidin beads containing varying concentrations of peptide 13 with a C-terminal biotin tag for 30 minutes (A,B). Following this incubation, the beads were separated from the supernatant using a magnet and 2.27 μg samples from each Eppendorf tube were analyzed by immunoblotting for remaining Myc-DDK tagged BACH1 in the supernatant. Additionally, Dynabeads-streptavidin without peptide was used as a negative control according to the amount of Dynabeads used in each Eppendorf tube, thereby analyzing background non-specific binding. All results were quantified by densitometric analysis. Densitometric data were normalized such that the Myc signal for the peptide tube containing 0 nM peptide was set to 100%, and the remaining amounts in other tubes were calculated as ratios relative to this amount. All these ratios and corresponding peptide concentrations were analyzed for dissociation constant estimation. Each groups was analyzed in duplicates. A) According to this analysis, the dissociation constant of peptide 13 for Myc-DDK tagged BACH1 was determined to be 187.8 nM (95% CI: 12.43-178 nM). This determined dissociation constant remained higher compared to the dissociation constant of peptide 13 against BACH2 (148.4 nM, 95% CI: 35.2-463.1 nM). This comparison indicated that peptide 13 may be a more BACH2-specific binder than BACH1. Furthermore, obtaining similar results in computational data supported our experimental findings. After weighing equal amount of from the peptide, concentration value in part B was calculated based on molecular weight and purity reported by the peptide manufacturer. CI: Confidence Interval, Rep: Replicate.

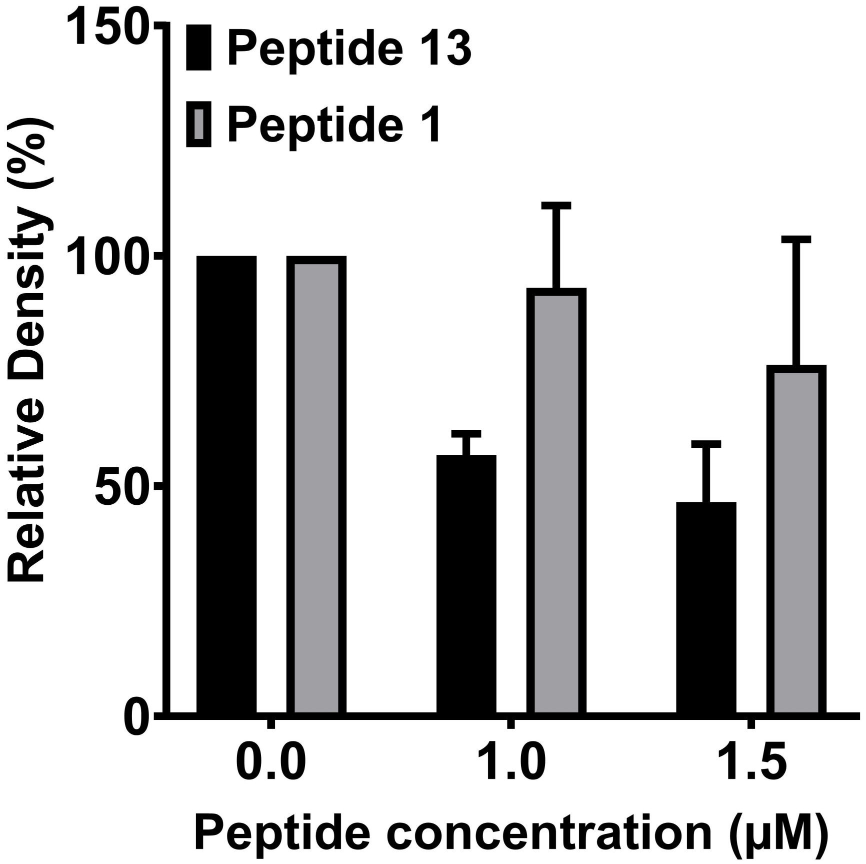

Supplementary Figure 11: Densitometric analysis of co-immunoprecipitation assay. Peptide 13 at concentrations of 1 μM and 1.5 μM demonstrated approximately 50% reduction compared to the control co-immunoprecipitation band, while the impact of the control peptide at identical concentrations on control immunoprecipitation remained more limited. Although no statistically significant difference was observed between groups, the 1 µM peptide 13 group approached the threshold of marginal statistical significance ($p < 0.05$) with a p-value of 0.068248 compared with the peptide 13 control (0 µM). This finding indicated that probable superiority in binding properties of peptide 13 relative to control peptide 1 that could be more profound observed at concentrations around 1 µM.

References

[1] Laskowski, R. A.; Swindells, M. B. LigPlot+: Multiple Ligand–Protein Interaction Diagrams for Drug Discovery. *Journal of Chemical Information and Modeling* **2011**, *51*, 2778–2786.

[2] Wallace, A. C.; Laskowski, R. A.; Thornton, J. M. LIGPLOT: a program to generate schematic diagrams of protein-ligand interactions. *Protein Engineering, Design and Selection* **1995**, *8*, 127–134.

[3] Adasme, M. F.; Linnemann, K. L.; Bolz, S. N.; Kaiser, F.; Salentin, S.; Haupt, V.; Schroeder, M. PLIP 2021: expanding the scope of the protein–ligand interaction profiler to DNA and RNA. *Nucleic Acids Research* **2021**, *49*, W530–W534.
